# Genomic diversity of bacteriophages infecting *Microbacterium* spp

**DOI:** 10.1371/journal.pone.0234636

**Published:** 2020-06-18

**Authors:** Deborah Jacobs-Sera, Lawrence A. Abad, Richard M. Alvey, Kirk R. Anders, Haley G. Aull, Suparna S. Bhalla, Lawrence S. Blumer, David W. Bollivar, J. Alfred Bonilla, Kristen A. Butela, Roy J. Coomans, Steven G. Cresawn, Tom D'Elia, Arturo Diaz, Ashley M. Divens, Nicholas P. Edgington, Gregory D. Frederick, Maria D. Gainey, Rebecca A. Garlena, Kenneth W. Grant, Susan M. R. Gurney, Heather L. Hendrickson, Lee E. Hughes, Margaret A. Kenna, Karen K. Klyczek, Hari Kotturi, Travis N. Mavrich, Angela L. McKinney, Evan C. Merkhofer, Jordan Moberg Parker, Sally D. Molloy, Denise L. Monti, Dana A. Pape-Zambito, Richard S. Pollenz, Welkin H. Pope, Nathan S. Reyna, Claire A. Rinehart, Daniel A. Russell, Christopher D. Shaffer, Viknesh Sivanathan, Ty H. Stoner, Joseph Stukey, C. Nicole Sunnen, Sara S. Tolsma, Philippos K. Tsourkas, Jamie R. Wallen, Vassie C. Ware, Marcie H. Warner, Jacqueline M. Washington, Kristi M. Westover, JoAnn L. Whitefleet-Smith, Helen I. Wiersma-Koch, Daniel C. Williams, Kira M. Zack, Graham F. Hatfull

**Affiliations:** 1 Department of Biological Sciences, University of Pittsburgh, Pittsburgh, Pennsylvania, United States of America; 2 Department of Biology, Illinois Wesleyan University, Bloomington, Illinois, United States of America; 3 Department of Biology, Gonzaga University, Spokane, Washington, United States of America; 4 Department of Natural Sciences, Mount Saint Mary College, Newburgh, New York, United States of America; 5 Department of Biology, Morehouse College, Atlanta, Georgia, United States of America; 6 Department of Biology, Illinois Wesleyan University, Bloomington, Illinois, United States of America; 7 Department of Biology, University of Wisconsin-River Falls, River Falls, Wisconsin, United States of America; 8 Department of Biology, North Carolina A&T State University, Greensboro, North Carolina, United States of America; 9 Department of Biology, James Madison University, Harrisonburg, Virginia, United States of America; 10 Department of Biological Sciences, Indian River State College, Fort Pierce, Florida, United States of America; 11 Department of Biology, La Sierra University, Riverside, California, United States of America; 12 Department of Biology, Southern Connecticut State University, New Haven, Connecticut, United States of America; 13 Department of Biology and Kinesiology, LeTourneau University, Longview, Texas, United States of America; 14 Department of Chemistry & Physics, Western Carolina University, Cullowhee, North Carolina, United States of America; 15 Department of Pathology, Wake Forest Baptist Health, Winston-Salem, North Carolina, United States of America; 16 Department of Biology, Drexel University, Philadelphia, Pennsylvania, United States of America; 17 School of Natural and Computational Sciences, Massey University, Auckland, New Zealand; 18 Department of Biological Sciences, University of North Texas, Denton, Texas, United States of America; 19 Department of Biological Sciences, Lehigh University, Bethlehem, Pennsylvania, United States of America; 20 Department of Biology, University of Central Oklahoma, Edmond, Oklahoma, United States of America; 21 Department of Biology, Nebraska Wesleyan University, Lincoln, Nebraska, United States of America; 22 Department of Microbiology, Immunology, & Molecular Genetics, University of California, Los Angeles, California, United States of America; 23 Department of Molecular and Biomedical Sciences, University of Maine, Orono, Maine, United States of America; 24 Department of Biology, University of Alabama at Birmingham, Birmingham, Alabama, United States of America; 25 Department of Biological Sciences, University of the Sciences in Philadelphia, Philadelphia, Pennsylvania, United States of America; 26 Department Cell Biology, Microbiology and Molecular Biology, University of South Florida, Tampa, Florida, United States of America; 27 Department of Biology, Ouachita Baptist University, Arkadelphia, Arkansas, United States of America; 28 Department of Biology, Western Kentucky University, Bowling Green, Kentucky, United States of America; 29 Department of Biology, University of Washington in St. Louis, St. Louis, Missouri, United States of America; 30 Howard Hughes Medical Institute, Chevy Chase, Maryland, United States of America; 31 Biology Department, Hope College, Holland, Michigan, United States of America; 32 Department of Biological Sciences, University of the Sciences, Philadelphia, Pennsylvania, United States of America; 33 Biology Department, Northwestern College, Orange City, Iowa, United States of America; 34 School of Life Sciences, University of Nevada, Las Vegas, Las Vegas, Nevada, United States of America; 35 Department of Natural Sciences, Nyack College, Nyack, New York, United States of America; 36 Department of Biology, Winthrop University, Rock Hill, South Carolina, United States of America; 37 Department of Biology & Biotechnology, Worcester Polytechnic Institute, Worcester, Massachusetts, United States of America; 38 Department of Biology, Coastal Carolina University, Conway, South Carolina, United States of America; Institut National de la Recherche Agronomique, FRANCE

## Abstract

The bacteriophage population is vast, dynamic, old, and genetically diverse. The genomics of phages that infect bacterial hosts in the phylum Actinobacteria show them to not only be diverse but also pervasively mosaic, and replete with genes of unknown function. To further explore this broad group of bacteriophages, we describe here the isolation and genomic characterization of 116 phages that infect *Microbacterium* spp. Most of the phages are lytic, and can be grouped into twelve clusters according to their overall relatedness; seven of the phages are singletons with no close relatives. Genome sizes vary from 17.3 kbp to 97.7 kbp, and their G+C% content ranges from 51.4% to 71.4%, compared to ~67% for their *Microbacterium* hosts. The phages were isolated on five different *Microbacterium* species, but typically do not efficiently infect strains beyond the one on which they were isolated. These *Microbacterium* phages contain many novel features, including very large viral genes (13.5 kbp) and unusual fusions of structural proteins, including a fusion of VIP2 toxin and a MuF-like protein into a single gene. These phages and their genetic components such as integration systems, recombineering tools, and phage-mediated delivery systems, will be useful resources for advancing *Microbacterium* genetics.

## Introduction

Genomic analyses of bacteriophages infecting bacterial hosts within the phylum Actinobacteria shows them to be genetically highly diverse, although the extent of diversity varies for phages infecting different hosts [1–4]. Of the more than 3,000 completely sequenced actinobacteriophage genomes [5], almost 1,800 were isolated on a single host strain, *Mycobacterium smegmatis* mc^2^155 [5]; smaller collections of phages isolated on *Gordonia* spp. [2, 6], *Cutibacterium* spp. (formerly *Propionibacterium* spp.) [4, 7], and *Arthrobacter* spp. [3] have also been described. Of these, the *Cutibacterium acnes* phages are the least diverse, and the others all demonstrate substantial genomic variation.

Nucleotide sequence similarity and shared gene content analyses provide an overview of the diversity of a group of phages [2, 8]. For example, the current collection of *Mycobacterium* phages can be grouped into 29 ‘clusters’ (e.g. Clusters A, B, C, …) and ten ‘singletons’, each of which has no close relatives. Twelve of those clusters have subgroups of phages distinct enough to be divided into ‘subclusters’, and there often is substantial sequence divergence within both subclusters and non-divided clusters. Moreover, the numbers of members in clusters and subclusters varies greatly; for example there are more than 600 members of Cluster A, divided into at least 18 subclusters (http://phagesdb.org), in marked contrast to the ten singleton phages. The groupings into cluster and subcluster likely reflect heterogenous sampling from a population with a continuum of diversity, which becomes more evident from larger sample sizes [2, 8]. The threshold values we have adopted for cluster inclusion–nucleotide sequence similarity spanning 50% genome lengths [9, 10], subsequently adjusted to 35% shared gene content [2]–are essentially arbitrary parameters, although they are useful for framing overall genomic diversity.

The expansive diversity of the actinobacteriophages–including a span of GC% content of ~40–70%–can be accommodated by a model in which rapid changes in host preferences facilitate escape from ongoing phage resistance of the bacterial hosts [11]. As phages migrate across a landscape of divergent bacterial hosts, they can access and acquire different genes from a large common gene pool. However, sampling of phages and genomic analyses are currently constrained to small numbers of bacterial species, and it is not yet possible to reconstruct these evolutionary pathways [11]. Isolation and characterization of phages using a greater variety of hosts within the Actinobacteria, should help to illuminate these models.

The *Microbacterium* spp. are high G+C% rod-shaped aerobes in the order Actinomycetales, which also contains the genera *Arthrobacter*, *Gordonia*, *Mycobacteria*, and *Streptomyces*. The *Microbacterium* spp. are in the family, *Microbacteriaceae*, whereas the other four genera are in the families *Micrococcaceae*, *Gordoniaceae*, *Mycobacteriaceae* and *Streptomycetaceae*, respectively. *Microbacterium* spp. are prevalent throughout the environment, having been isolated from soil, plants, and food [12–14]. *Microbacterium* spp. have been shown to benefit some plants by increasing drought resistance [15], have been associated with bacteremia in patients [16–19], and have been isolated from a cystic fibrosis patient [20, 21]. Most *Microbacterium* strains do not carry CRISPR-Cas systems [22, 23]; some *Microbacterium* restriction-modification systems have been reported [24, 25]. *Microbacterium* spp. do not contain mycolic acids in their cell walls [26–28]. Only two *Microbacterium* phages have been described previously. One, Min1, was isolated using *Microbacterium nematophilum*–a nematode pathogen–as a host [29]. Min1 is reported to have siphoviral morphology, and is temperate, integrated into a stable plasmid, pMN1[29]. The other, vB_MoxS-ISF9, is a lytic phage with siphoviral morphology, isolated from sewage on *Microbacterium oxydans* [30].

Here, we report the comparative genomic analyses of 116 individual bacteriophages isolated on several different strains and species of *Microbacterium*. They are genetically diverse and can be grouped into 12 clusters and seven singletons; they also vary considerably in G+C% content, ranging from 51.4% to 71.4% (*Microbacterium* genomes are ~67% G+C%). Most of the phages are predicted to be strictly lytic—only three are predicted to be temperate—in contrast to the *Mycobacterium* phages in which over 50% of sequenced genomes are temperate [1].

## Results and discussion

### Microbacterium phage isolation

**Fig 1:**
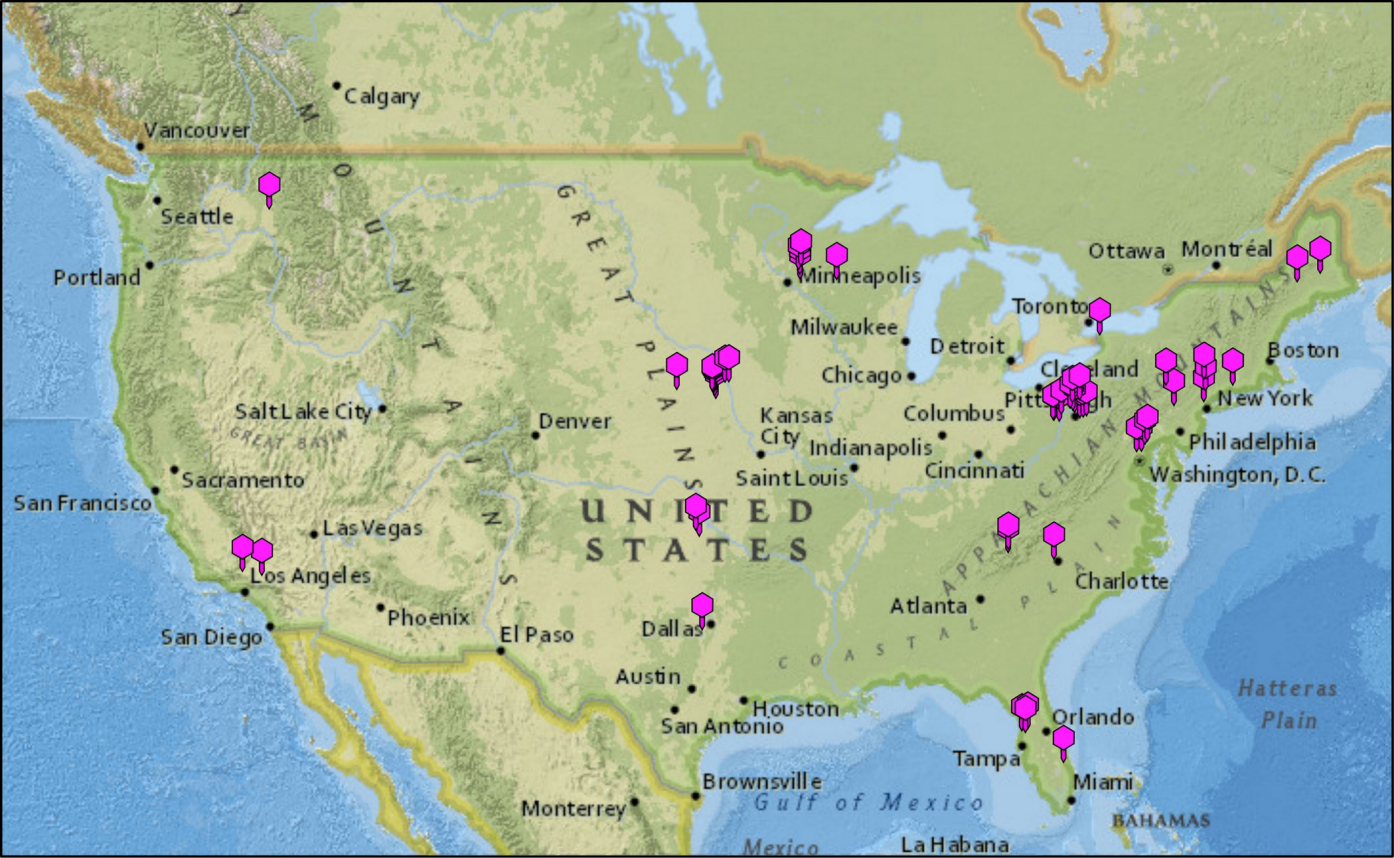
Geographical distribution of *Microbacterium* phages. Geographic distribution of the isolation sites of *Microbacterium* phages with completely sequenced genomes.

One hundred and sixteen phages were isolated from environmental samples (mostly soil and compost) using five different *Microbacterium* spp. hosts, either by direct plating or by enrichment (Table 1; https://phagesdb.org) [31]. The majority of these phages (95) were isolated using *Microbacterium foliorum* NRRL B-24224 with smaller numbers on *Microbacterium paraoxydans* NWU1 (14), *Microbacterium paraoxydans* NRRL B-148343 (4), *Microbacterium aerolatum* NRRL B-24288 (2), and *Microbacterium natoriense* ATCC BAA-1032 (1). The phages were isolated by students in the Phage Hunters Integrating Research and Education (PHIRE) at University of Pittsburgh and Science Education Alliance Phage Hunters Advancing Genomics and Evolutionary Science (SEA-PHAGES) programs at Carnegie Mellon University, Howard Hughes Medical Institute, Indian River State College, La Sierra University, Lehigh University, Mount Saint Mary College, Nyack College, Nebraska Wesleyan University, Seton Hill University, Southern Connecticut State University, University of Central Oklahoma, University of Maine Honors College, University of Pittsburgh, University of South Florida, University of West Florida, University of Wisconsin-River Falls, Western Carolina University, and Winthrop University. The isolation locations are broadly but unevenly distributed geographically across the United States (Fig 1). The phages were identified as plaque forming units on lawns of the bacterial hosts, purified, and amplified. Genomic DNA was extracted as described previously [32]. Most of the phages form clear plaques on their isolation host and are presumed to be obligatorily lytic, with the exceptions of phages Floof and Percival (Cluster EH), as well as Zeta1847, which form turbid plaques and may be temperate.

**Table 1:** Genometrics of *Microbacterium* phages.

Phage Name	Cluster	Host^1^	Length^2^	GC%	Termini^3^	Morphology^4^	ORFs	tRNAs	lytic/temp^5^	Accession #^6^
AlexAdler	EA1	Mfo NRRL B-24224	41834	63.4	Cir per	Siphoviridae	63	0	lytic	MG962360
Antoinette	EA1	Mfo NRRL B-24224	41858	63.4	Cir per	Siphoviridae	63	0	lytic	MH045565
Aubergine	EA1	Mfo NRRL B-24224	41555	63.4	Cir per	Siphoviridae	62	0	lytic	MG839015
AxiPup	EA1	Mfo NRRL B-24224	41770	63.3	Cir per	Siphoviridae	63	0	lytic	MG839016
Baines	EA1	Mfo NRRL B-24224	41555	63.4	Cir per	Siphoviridae	62	0	lytic	MG839017
Balsa	EA1	Mfo NRRL B-24224	41862	63.4	Cir per	Siphoviridae	62	0	lytic	MG839030
Bandik	EA1	Mfo NRRL B-24224	41804	63.5	Cir per	Siphoviridae	63	0	lytic	MH045554
BeeBee8	EA1	Mfo NRRL B-24224	42027	63.3	Cir per	Siphoviridae	64	0	lytic	MH045555
Bonino	EA1	Mfo NRRL B-24224	41534	63.4	Cir per	Siphoviridae	62	0	lytic	MG920061
Dave	EA1	Mfo NRRL B-24224	41858	63.4	Cir per	Siphoviridae	63	0	lytic	MH045558
Espinosa	EA1	Mfo NRRL B-24224	41553	63.4	Cir per	Siphoviridae	62	0	lytic	MG839018
Etna	EA1	Mfo NRRL B-24224	41908	63.4	Cir per	Siphoviridae	63	0	lytic	MH045559
Gargoyle	EA1	Mfo NRRL B-24224	41803	63.5	Cir per	Siphoviridae	63	0	lytic	MH153802
Gelo	EA1	Mfo NRRL B-24224	41562	63.4	Cir per	Siphoviridae	62	0	lytic	MG962367
Hamlet	EA1	Mfo NRRL B-24224	41934	63.4	Cir per	Siphoviridae	63	0	lytic	MG839019
Ilzat	EA1	Mfo NRRL B-24224	41525	63.5	Cir per	Siphoviridae	62	0	lytic	MG839029
Kale	EA1	Mfo NRRL B-24224	41558	63.4	Cir per	Siphoviridae	62	0	lytic	MG839020
Knox	EA1	Mfo NRRL B-24224	41797	63.3	Cir per	Siphoviridae	63	0	lytic	MG839021
Ludgate	EA1	Mfo NRRL B-24224	41799	63.5	Cir per	Siphoviridae	63	0	lytic	MG839022
Martin	EA1	Mfo NRRL B-24224	41812	63.5	Cir per	Siphoviridae	63	0	lytic	MH153805
Nagem	EA1	Mfo NRRL B-24224	41846	63.5	Cir per	Siphoviridae	63	0	lytic	MH045560
Nattles	EA1	Mfo NRRL B-24224	41544	63.4	Cir per	Siphoviridae	62	0	lytic	MG925352
Oats	EA1	Mfo NRRL B-24224	41555	63.4	Cir per	Siphoviridae	62	0	lytic	MH153806
Papafritta	EA1	Mfo NRRL B-24224	41573	63.4	Cir per	Siphoviridae	62	0	lytic	MH513981
Peep	EA1	Mfo NRRL B-24224	41856	63.4	Cir per	Siphoviridae	63	0	lytic	MG839023
Peppino	EA1	Mfo NRRL B-24224	41932	63.3	Cir per	Siphoviridae	63	0	lytic	MG839024
PuppyEggo	EA1	Mfo NRRL B-24224	41803	63.3	Cir per	Siphoviridae	63	0	lytic	MG944219
Raccoon	EA1	Mfo NRRL B-24224	41894	63.4	Cir per	Siphoviridae	63	0	lytic	MG839025
Raptor	EA1	Mfo NRRL B-24224	41801	63.4	Cir per	Siphoviridae	63	0	lytic	MH045562
Redfield	EA1	Mfo NRRL B-24224	41930	63.3	Cir per	Siphoviridae	63	0	lytic	MH479922
Robinson	EA1	Mfo NRRL B-24224	41874	63.5	Cir per	Siphoviridae	63	0	lytic	MH045563
Schnapsidee	EA1	Mfo NRRL B-24224	41872	63.4	Cir per	Siphoviridae	63	0	lytic	MH590590
StingRay	EA1	Mfo NRRL B-24224	41597	63.5	Cir per	Siphoviridae	62	0	lytic	MG944222
Superfresh	EA1	Mfo NRRL B-24224	41860	63.4	Cir per	Siphoviridae	63	0	lytic	MG839026
Teagan	EA1	Mfo NRRL B-24224	41793	63.5	Cir per	Siphoviridae	63	0	lytic	MH153811
TeddyBear	EA1	Mfo NRRL B-24224	41555	63.4	Cir per	Siphoviridae	62	0	lytic	MH045564
Tenda	EA1	Mfo NRRL B-24224	41553	63.4	Cir per	Siphoviridae	62	0	lytic	MG839028
Andromedas	EA2	Mfo NRRL B-24224	40494	62	Cir per	Siphoviridae	63	1	lytic	MH590606
ColaCorta	EA2	Mfo NRRL B-24224	40494	62	Cir per	Siphoviridae	63	1	lytic	MH590604
Eleri	EA2	Mfo NRRL B-24224	40366	62	Cir per	Siphoviridae	63	0	lytic	MG839027
Sansa	EA2	Mfo NRRL B-24224	40306	61.8	Cir per	Siphoviridae	62	1	lytic	MH513982
Casey	EA3	Mfo NRRL B-24224	39307	61.3	Cir per	Siphoviridae	59	1	lytic	MG944226
Pajaza	EA3	Mfo NRRL B-24224	39307	61.2	Cir per	Siphoviridae	59	1	lytic	MG944216
Pikmin	EA3	Mfo NRRL B-24224	39307	61.3	Cir per	Siphoviridae	59	1	lytic	MG944218
Golden	EA4	Mfo NRRL B-24224	39640	64.1	Cir per	Siphoviridae	58	1	lytic	MG925343
Koji	EA4	Mfo NRRL B-24224	39403	64.2	Cir per	Siphoviridae	56	1	lytic	MG925345
Lucky3	EA4	Mfo NRRL B-24224	39640	64.1	Cir per	Siphoviridae	58	1	lytic	MG925347
Sinatra	EA4	Mfo NRRL B-24224	39183	64.4	Cir per	Siphoviridae	57	1	lytic	MK937602
Neferthena	EA5	Mfo NRRL B-24224	41706	64.4	Cir per	Siphoviridae	62	1	lytic	MH697589
Chepli	EA6	Mfo NRRL B-24224	40332	63	Cir per	Siphoviridae	64	0	lytic	MK875794
Theresita	EA7	Mna ATCC BAA1032	40234	65.9	Cir per	Siphoviridae	57	0	lytic	MK660713
Schubert	EA8	Mfo NRRL B-24224	38820	61.4	Cir per	Siphoviridae	55	1	lytic	MK308637
Armstrong	EB	Mfo NRRL B-24224	39928	67.1	3’ 10-b ext	Siphoviridae	68	2	lytic	MH834596
Arroyo	EB	Mfo NRRL B-24224	42129	66.6	3’ 10-b ext	Siphoviridae	69	3	lytic	MK937610
Bernstein	EB	Mfo NRRL B-24224	39926	67.1	3’ 10-b ext	Siphoviridae	68	2	lytic	MH834599
Brahms	EB	Mfo NRRL B-24224	39828	67.1	3’ 10-b ext	Siphoviridae	68	2	lytic	MH834602
Coltrane	EB	Mfo NRRL B-24224	39828	67.1	3’ 10-b ext	Siphoviridae	68	2	lytic	MH834604
Didgeridoo	EB	Mfo NRRL B-24224	42655	66.1	3’ 10-b ext	Siphoviridae	74	1	lytic	MH045566
Dismas	EB	Mfo NRRL B-24224	41593	69.6	3’ 10-b ext	Siphoviridae	66	1	lytic	MG670586
Eden	EB	Mfo NRRL B-24224	40833	66.3	3’ 10-b ext	Siphoviridae	69	3	lytic	MH509447
Elva	EB	Mfo NRRL B-24224	42139	68.2	3’ 10-b ext	Siphoviridae	71	3	lytic	MH045567
Kieran	EB	Mfo NRRL B-24224	41417	69.7	3’ 10-b ext	Siphoviridae	64	1	lytic	MH045568
Rollins	EB	Mfo NRRL B-24224	38926	67.1	3’ 10-b ext	Siphoviridae	68	2	lytic	MH834626
Fireman	EC	Mpa NWU1	54579	68.6	Cir per	Siphoviridae	96	0	lytic	MK524510
KaiHaiDragon	EC	Mfo NRRL B-24224	52992	68.9	Cir per	Siphoviridae	92	0	lytic	MH590600
Metamorphoo	EC	Mpa NWU1	54148	68.5	Cir per	Siphoviridae	93	0	lytic	MH271304
Paschalis	EC	Mfo NRRL B-24224	52935	68.8	Cir per	Siphoviridae	91	0	lytic	MH155873
Quhwah	EC	Mfo NRRL B-24224	53549	68.8	Cir per	Siphoviridae	95	0	lytic	MH271321
RobsFeet	EC	Mpa NWU1	54189	68.6	Cir per	Siphoviridae	99	0	lytic	MH271312
Alleb	ED1	Mpa NWU1	62642	64.5	3205bp DR	Siphoviridae	115	4	lytic	MK376963
Hortus1	ED1	Mpa NWU1	63119	64.6	3159bp DR	Siphoviridae	114	4	lytic	MH271300
Jacko	ED1	Mpa NRRL B-14843	61421	64.9	3028bp DR	Siphoviridae	115	2	lytic	MH399779
OlinDD	ED1	Mpa NWU1	63123	64.6	3158bp DR	Siphoviridae	114	4	lytic	MH271307
Pioneer3	ED1	Mpa NWU1	62954	64.6	3191bp DR	Siphoviridae	114	4	lytic	MH271310
Tandem	ED1	Mpa NWU1	63128	64.7	3285bp DR	Siphoviridae	114	4	lytic	MH271316
Fork	ED2	Mfo NRRL B-24224	62090	61.8	3099bp DR	Siphoviridae	117	3	lytic	MH371108
Lyell	ED2	Mfo NRRL B-24224	62716	61.6	3549bp DR	Siphoviridae	122	3	lytic	MH371109
Musetta	ED2	Mfo NRRL B-24224	63604	61.7	3809bp DR	Siphoviridae	119	4	lytic	MH536823
BonaeVitae	EE	Mpa NWU1	17451	68.2	3’ 9-b ext	Siphoviridae	26	0	lytic	MH045556
BurtonThePup	EE	Mfo NRRL B-24224	17445	68.8	3’ 9-b ext	Siphoviridae	25	0	lytic	MH045557
Dongwon	EE	Mfo NRRL B-24224	17362	68.5	3’ 9-b ext	Siphoviridae	25	0	lytic	MH744416
Efeko	EE	Mpa NRRL B-14843	17491	68.6	3’ 9-b ext	Siphoviridae	28	0	lytic	MH825700
KayPaulus	EE	Mfo NRRL B-24224	17455	68.5	3’ 9-b ext	Siphoviridae	25	0	lytic	MH371118
Miaurora	EE	Mfo NRRL B-24224	17032	69	3’ 9-b ext	Siphoviridae	25	0	lytic	MH779512
Minima	EE	Mfo NRRL B-24224	17362	68.5	3’ 9-b ext	Siphoviridae	25	0	lytic	MH651181
Noelani	EE	Mfo NRRL B-24224	17349	68.2	3’ 9-b ext	Siphoviridae	25	0	lytic	MH399783
PaoPu	EE	Mfo NRRL B-24224	17362	68.5	3’ 9-b ext	Siphoviridae	25	0	lytic	MH045561
Quaker	EE	Mfo NRRL B-24224	17450	68.6	3’ 9-b ext	Siphoviridae	25	0	lytic	MH371111
Scamander	EE	Mfo NRRL B-24224	17452	68.7	3’ 9-b ext	Siphoviridae	25	0	lytic	MH576963
TimoTea	EE	Mfo NRRL B-24224	17427	68.7	3’ 9-b ext	Siphoviridae	25	0	lytic	MK524502
VitulaEligans	EE	Mfo NRRL B-24224	17534	68.8	3’ 9-b ext	Siphoviridae	25	0	lytic	MH371124
AnnaSerena	EF	Mfo NRRL B-24224	56707	63.8	Cir per	Siphoviridae	83	0	lytic	MH271292
Krampus	EF	Mfo NRRL B-24224	56708	63.8	Cir per	Siphoviridae	83	0	lytic	MH271301
Hyperion	EG	Mfo NRRL B-24224	61769	67	203bp DR	Siphoviridae	104	0	lytic	MH153803
OneinaGillian	EG	Mfo NRRL B-24224	61703	67.1	203bp DR	Siphoviridae	103	0	lytic	MH727556
Squash	EG	Mfo NRRL B-24224	62312	66.8	204bp DR	Siphoviridae	108	0	lytic	MH153813
Floof	EH	Mfo NRRL B-24224	48500	69	3’ 11-b ext	Siphoviridae	80	1	temperate	MH271298
Percival	EH	Mfo NRRL B-24224	47364	69.6	3’ 11-b ext	Siphoviridae	74	1	temperate	MH271308
Cinna	EI	Mpa NWU1	55366	70	Cir per	Siphoviridae	93	1	lytic	MK937591
Cressida	EI	Mpa NWU1	55957	70.3	Cir per	Siphoviridae	95	1	lytic	MK937608
Margaery	EI	Mpa NWU1	56033	69.5	Cir per	Siphoviridae	96	1	lytic	MK937606
MementoMori	EI	Mpa NWU1	55572	70	Cir per	Siphoviridae	96	1	lytic	MH271303
Goodman	EJ	Mfo NRRL B-24224	42363	66.3	Cir per	Siphoviridae	62	0	lytic	MK016495
Johann	EJ	Mfo NRRL B-24224	42363	66.3	Cir per	Siphoviridae	62	0	lytic	MK016497
ArMaWen	EK1	Mfo NRRL B-24224	53939	59.9	Cir per	Podoviridae	54	0	lytic	MK308638
TinyTimothy	EK1	Mfo NRRL B-24224	53932	56.8	Cir per	Podoviridae	53	0	lytic	MK878904
Akoni	EK2	Mfo NRRL B-24224	54307	60.1	Cir per	Podoviridae	55	0	lytic	MK757449
Camille	EL	Mae NRRL B-24228	53097	56.3	3’ 9-b ext	Siphoviridae	81	0	lytic	MH153800
Count	EL	Mae NRRL B-24228	78922	51.4	3’ 9-b ext	Siphoviridae	131	2	lytic	MH153801
Appa	Singleton	Mfo NRRL B-24224	38684	68	Cir per	Siphoviridae	65	1	lytic	MH153799
Burro	Singleton	Mpa NRRL B-14843	54473	64.3	Cir per	Podoviridae	49	0	lytic	MH825698
Hendrix	Singleton	Mfo NRRL B-24224	97757	66.1	Cir per	Siphoviridae	155	4	lytic	MH183162
Min1	Singleton	Mne CBX102	46365	68.3	Cir per	Unknown	77	0	lytic	EF579802
Triscuit	Singleton	Mfo NRRL B-24224	67539	58.3	3795bp DR	Siphoviridae	112	1	lytic	MH047631
ValentiniPuff	Singleton	Mpa NRRL B-14843	62517	67.4	Cir per	Siphoviridae	112	0	lytic	MH825712
Zeta1847	Singleton	Mpa NWU1	47921	71.4	3’ 11-b ext	Siphoviridae	75	1	temperate	MH271320

^1^Mfo NRRL B-24224: *Microbacterium foliorum* NRRL B-24224; Mna ATCC BAA1032; *Microbacterium natoriense* ATCC BAA-1032; Mpa NWU1: *Microbacterium paraoxydans* NWU1; Mpa NRRL B14843: *Microbacterium paraoxydans* NRRL B-14843; Mae NRRL B-24228: *Microbacterium aerolatum* NRRL B-24228; Mne CBX102: *Microbacterium nematophilum* CBX102.

^2^Genome length in base pairs

^3^Genome termini. Cir per: circularly permuted; ext: extension; DR: Direct Repeat

^4^Virion morphology, determined by electron microscopy

^5^Lytic or temperate life style, as predicted bioinformatically

^6^GenBank Accession number

**Fig 2:**
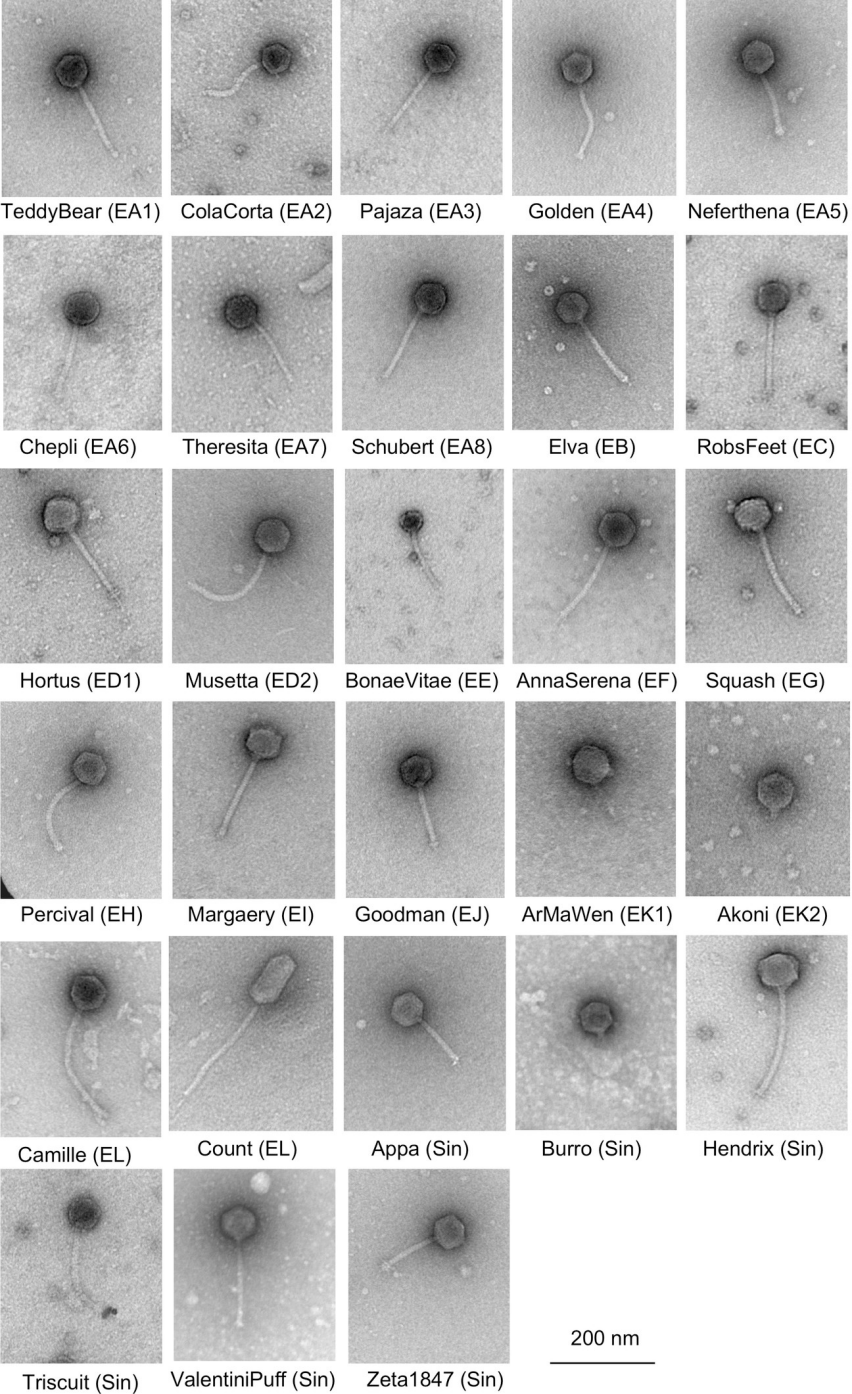
*Microbacterium* phage virion morphologies. Representative virion particles of each *Microbacterium* phage cluster show a predominance of *siphoviridae* morphologies; Cluster EK and singleton Burro are *podoviridae*. All have isometric capsids with the exception of Count, which has a prolate capsid.

All of the phages isolated on *Microbacterium* spp. are part of the *Caudovirales* with dsDNA genomes and tailed phage morphologies (Fig 2). Phage particles for a representative subset of the phages (see below) were examined by transmission electron microscopy; most have siphoviral morphologies with flexible non-contractile tails, although a few (e.g. Burro, ArMaWen, Akoni) are podoviruses with very short tails (Fig 2). With the exceptions of the prolate-headed phage Count, all have isometric capsids; no myoviruses were found. The finding of *Microbacterium* podoviruses is of interest as these are quite rare among actinobacteriophages, and have been reported for *Arthrobacter* phages but not for mycobacteriophages [5].

### Microbacterium phage sequencing and genometrics

With the exception of the previously published Min1 [29], all phages were sequenced at either Western Carolina University or the Pittsburgh Bacteriophage Institute. Genomes were annotated using a previously reported pipeline involving automated gene predictions followed by careful manual inspection and revision [33]. GenBank accession numbers are shown in Table 1.

*Microbacterium* phage genome sizes range from 17,032 bp (Miaurora) to 97,757 bp (Hendrix), and the viral genomes have a variety of types of termini, including defined cohesive ends (with 8–11 base single-stranded 3’ DNA extensions), direct terminal repeats (DTRs) ranging from 203 bp (Hyperion) to 3,809 bp (Musetta), and circularly permuted headful-packed genomes (Table 1). The G+C% contents vary from 51.4% (Count) to 71.4% (Zeta1847); the host G+C% is 67–68%. The numbers of predicted open reading frames (ORFs) range from as few as 25 (BurtonThePup) to as many as 155 (Hendrix) (Table 1). The predicted gene products were assorted into phamilies (phams) using similar metrics to those described previously [8, 10, 34], and genome maps displayed using a browser-accessible version of Phamerator [34]. Approximately one-third of the phages code for at least one tRNA, but none have more than four tRNA genes (Table 1).

### Microbacterium cluster assignments

**Fig 3:**
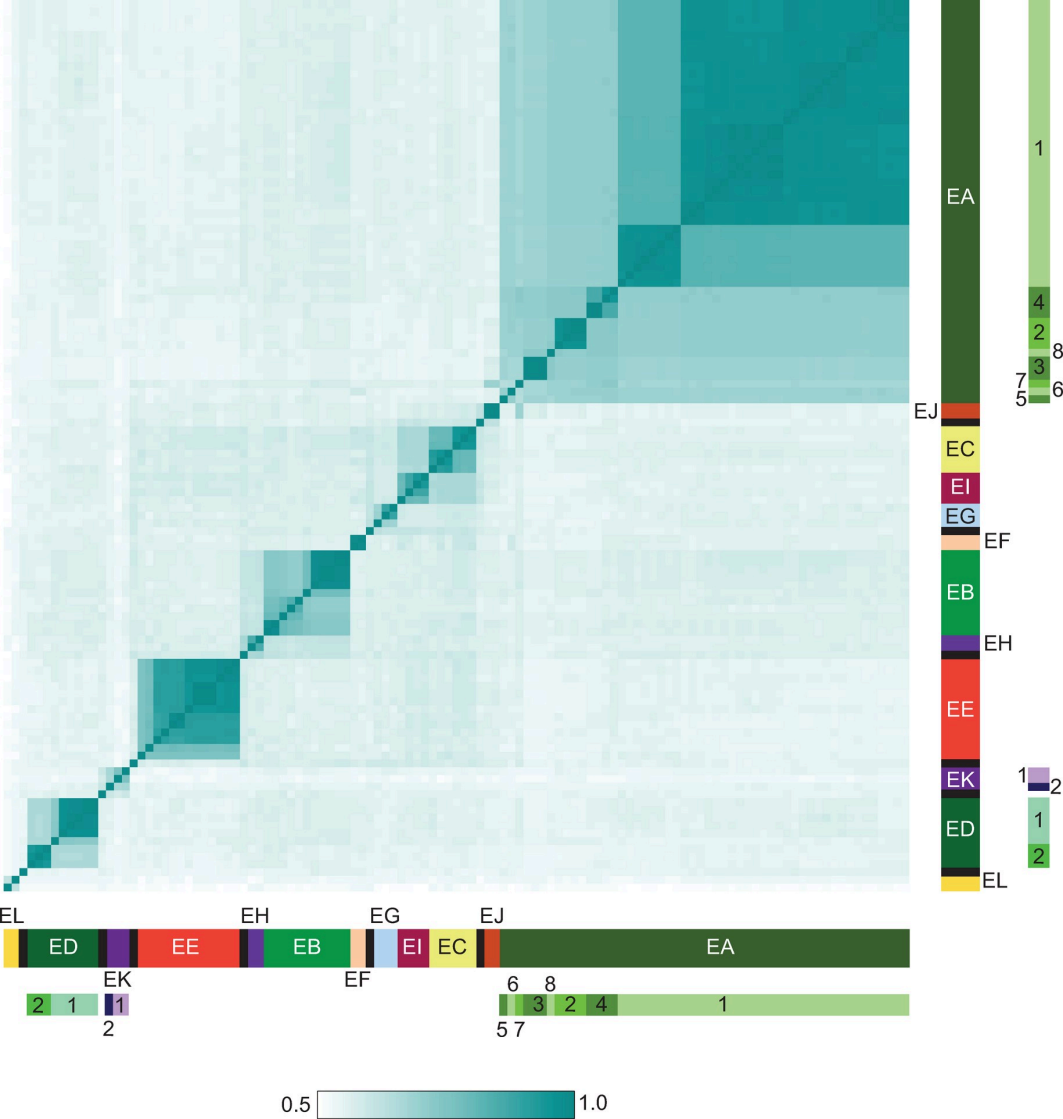
Heatmap of *Microbacterium* phage average nucleotide identities (ANIs). Pairwise average nucleotide identities were calculated for 116 *Microbacterium* phages using DNA Master with default settings. The heatmap was generated using R and the ‘heatmap2’ function, which determines distances between each genome and calculates the optimal genome order for representation, using distance parameter and clustering methods of ‘maximum’ and ‘single’, respectively. Genome clusters are shown on the axes, colored according to cluster, with singletons show in black; subclusters are indicated with numbers alongside their cluster designations. Phage vB_MosX-ISF9 genome is not included in these analyses [30].

**Fig 4:**
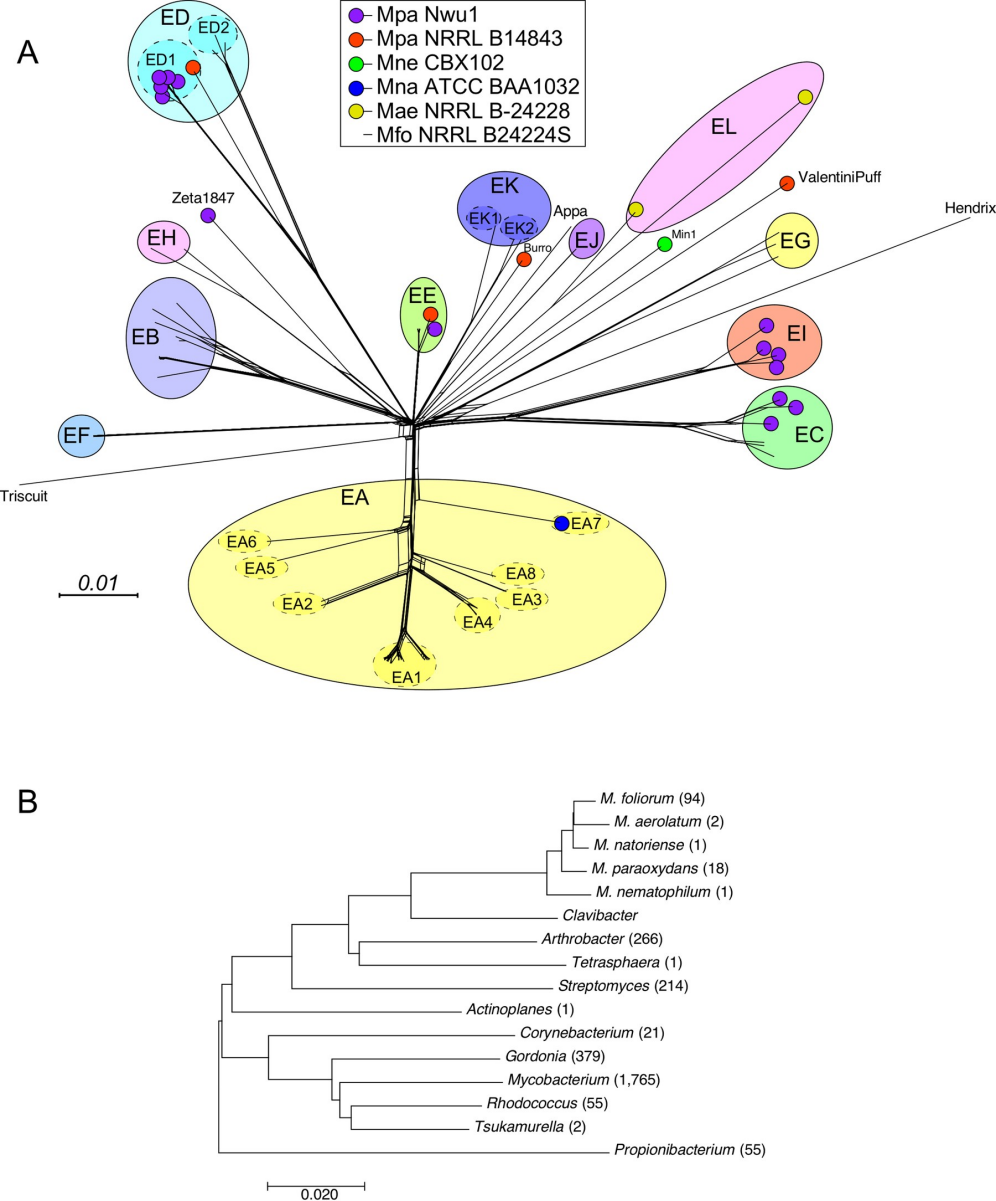
Relationships among *Microbacterium* phages and their bacterial hosts. **A.** A network phylogeny of *Microbacterium* phages. The predicted proteins of all 116 *Microbacterium* phage were sorted into 1975 phamilies (phams) according to shared amino acid sequence similarities using Phamerator [34]. Each genome was then assigned a value reflecting the presence or absence of a pham member; the genomes were compared and displayed using Splitstree [35]. The clusters and subclusters derived from dotplot and ANI comparisons are indicated with larger colored circles. Smaller colored circles at the nodes indicate the bacterium host used for isolation, as noted in the key. Phages isolated on *M*. *foliorum* NRRL B-24224 have no small colored circle at the node. The scale bar indicates 0.01 substitutions/site. **B**. Evolutionary relationships of bacterial host taxa, using MEGA7 [36–38]. The number of phages isolated on each host is shown in parentheses.

Nucleotide sequence comparisons of the *Microbacterium* phages reveals that there are distinct groups that share little sequence similarity with each other (Fig 3). The phages were grouped into clusters if they shared 35% or more of their genes [2, 8]. These can be visually represented by a gene content-based network phylogeny (Fig 4A). The phages form 12 clusters (Clusters EA-EL) and there are seven singletons (including the previously reported Min1), each with no close relative (Table 1). Using average nucleotide identity (ANI) comparisons, three clusters (EA, ED, and EK) were divided into several subclusters (Tables 1 and S1). The distribution of individual phages across cluster/singleton types is heterogenous, with almost 45% of phages grouped in Cluster EA (Table 1).

The network phylogeny based on shared gene content (Fig 4A) illustrates the relationships among these phages. Most clusters/singletons share few if any genes with each other, and for some groups the intra-cluster diversity is reflected in relatively deep branches (e.g. within Clusters EG and ED). There are also examples of clusters (e.g. EC and EI) that share both some nucleotide sequence similarity and related gene products, but fall below the current thresholds for grouping together (Fig 4A and S2 Table). There is also unequal distribution of phage types (i.e. cluster, subcluster, singleton designations) in regards to the host used for isolation (Fig 4B). For example, although Cluster EA phages are over-represented, all but one were isolated on *M*. *foliorum* (Fig 4A and Table 1), raising the question as to whether Cluster EA phages can infect other *Microbacterium* strains. Conversely, Cluster ED phages were isolated on three different hosts and may have somewhat broader host ranges (Fig 4A and Table 1).

### Genomic characteristics of microbacterium phages

#### General genomic features

The *Microbacterium* phage genomes vary in length (17.3 kbp to 97.7 kbp), and their G+C% contents (51.4% to 71.4%) and the linear viral genomes have a variety of termini reflecting different DNA packaging mechanisms (Table 1). Clusters EA, EC, EF, EI, EJ, EK and the singletons Appa, Burro, and Hendrix are all circularly permuted and (presumably) terminally redundant, consistent with headful packaging systems. Clusters EB, EE, EH, EL, and the singleton Zeta1847 all have defined ends with short single-strand 3’ DNA extensions reflecting cos-type packaging; Clusters ED, EG, and the singleton Triscuit all have DTRs. Most of the phages form clear plaques and are presumably obligatorily lytic, with the exceptions of Zeta1847 and the Cluster EH phages (Floof and Percival), which may be temperate. Only Zeta1847 and the Cluster EH phages code for integrases (serine-family Int’s), although repressor genes have not been identified and it is not clear if these are true temperate phages. None of the other phages have any genomic characteristics indicative of a temperate lifestyle.

The *Microbacterium* phages have several types of genomic architecture. The most common is in Clusters EB, EC, EE, EF, EH, EI, EJ, El, and the singletons Appa, Burro, Hendrix, ValentiniPuff, and Zeta1847, in which most or all of the genes are rightwards-transcribed, with the virion structure and assembly genes located in the leftmost parts of their genomes; at most, only 1–3 genes are leftwards-transcribed. In contrast, phages in Clusters EA, ED, and EG have two large sets of genes, with those in the left and right halves of the genomes rightwards- and leftwards-transcribed, respectively. However, in Cluster ED there are seven leftwards-transcribed genes in the DTR, and in Cluster EG there is a set of leftwards-transcribed genes between the left genome end and the structural genes. In the Cluster EK phages and the singleton Burro, the leftwards and rightwards-transcribed genes occupy the left and right parts of the genome respectively. In the singleton Triscuit, genes in the left two-thirds of the genome are all rightwards-transcribed, and in the rightmost one-third, sets of genes alternate the direction of transcription. In most of the genomes the lysis cassette is located immediately downstream from the tail genes, with the notable exceptions of Cluster EL and singleton Triscuit, where it is to the left of the structural genes. The lysis cassettes typically contain an endolysin gene and one or more membrane protein genes coding for the holin. None of the *Microbacterium* phages contain a lysin B gene that is common in mycobacteriophages, an unsurprising result given the lack of a mycolic acid outer layer in these hosts.

#### Cluster EA

**Fig 5:**
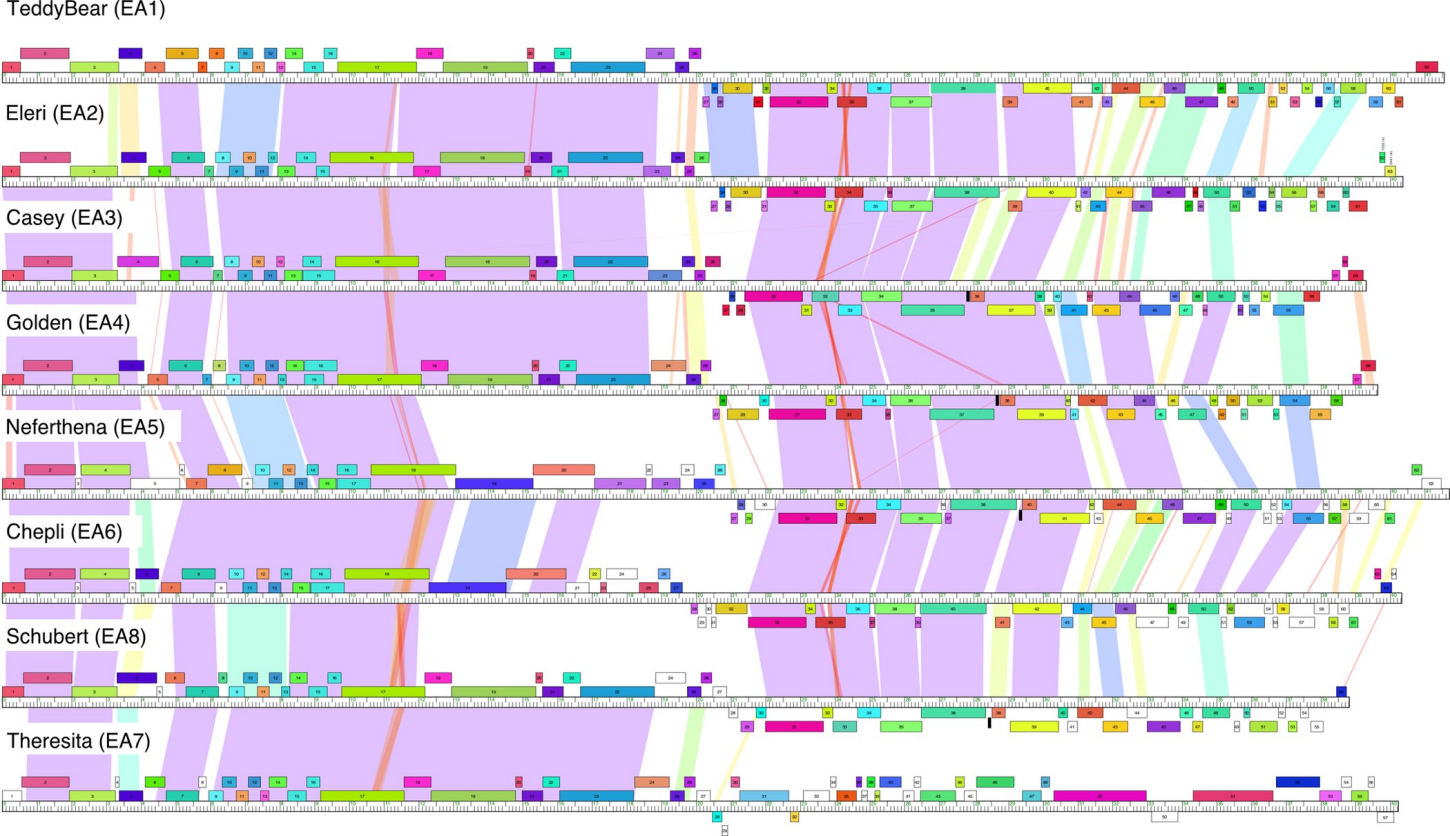
Pairwise alignment of *Microbacterium* phage Cluster EA genomes. A representative genome from each EA subcluster phage is shown. The sole Subcluster EA7 phage Theresita is shown at the bottom as it is substantially different from the others in the right halves of the genomes. Pairwise nucleotide sequence similarities are displayed with spectrum-coloring between genomes, with violet representing greatest similarity and red the least similar, above a threshold E value of 10^−5^. Genes are represented as boxes above or below the genomes reflecting rightwards- and leftwards-transcription respectively. Genes are colored according to their phamily designations using Phamerator [34] and database Actinobacteriophage_2422. White boxes represent ‘orphams’, genes with no close relatives in this dataset.

**Fig 6:**
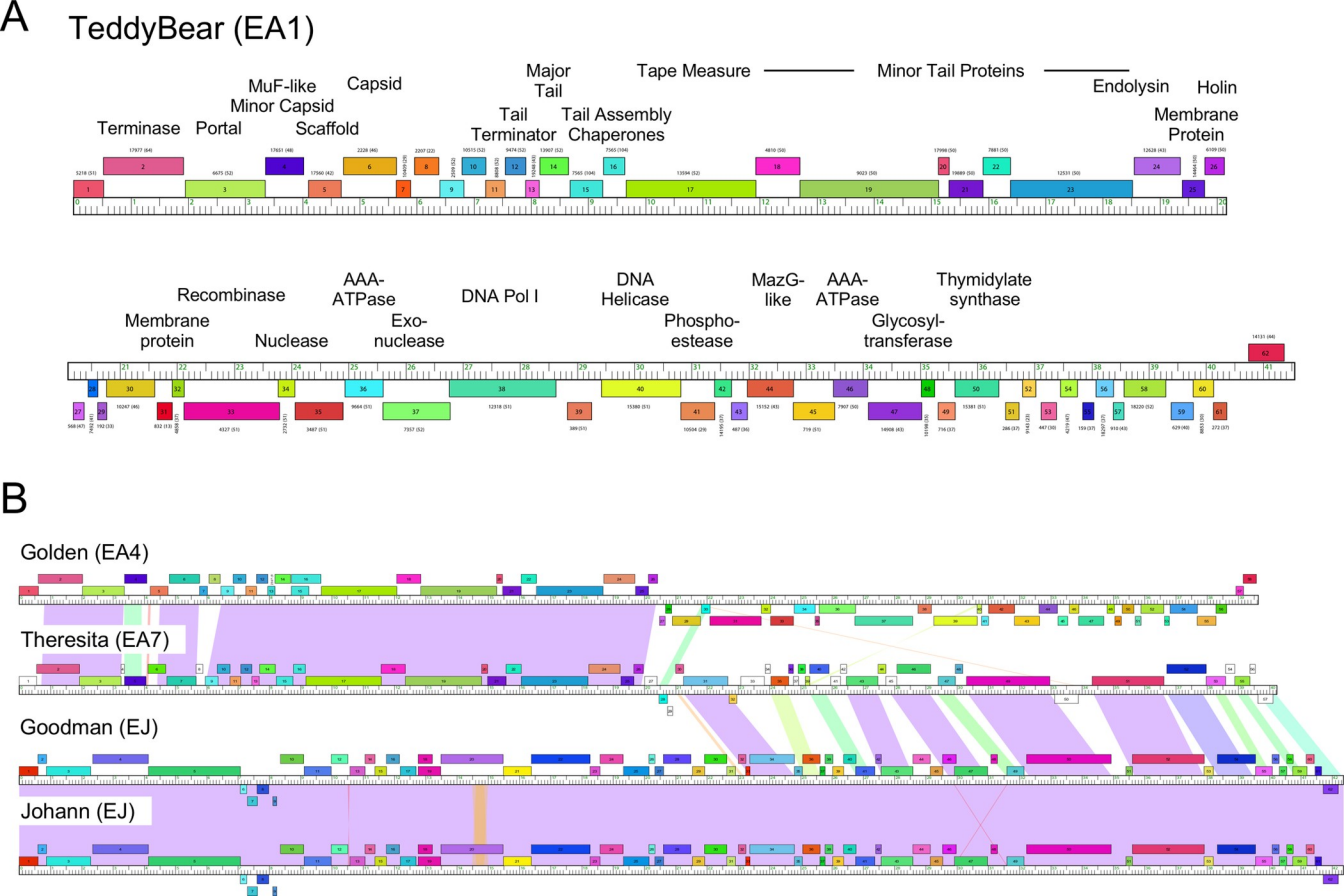
Genome organization of *Microbacterium* Subcluster EA1 phage TeddyBear. **A**. The genome of *Microbacterium* phage TeddyBear is shown with predicted genes represented as boxes above or below the genome reflecting rightwards- and leftwards-transcription respectively. Genes are colored according to their phamily designations using Phamerator [34] and database Actinobacteriophage_2422. The phamily numbers shown above each gene with the number of phamily members in parentheses. **B. Pairwise alignment of *Microbacterium* phages Golden, Theresita, Goodman, and Johann genomes.** See Fig 5 for map details. Theresita shares 45.2% and 23.6% average gene content with Golden and Goodman, respectively.

Almost half of the *Microbacterium* phages isolated here group into Cluster EA (52 out of 116). These have been divided into eight subclusters (EA1 –EA8) with EA1 being the largest (37 members; Table 1). Alignment of genome maps (Fig 5) shows that Subclusters EA1- EA8 have common architectures with rightwards- and leftwards-transcribed genes in the left and right halves of the genomes, respectively. The Subcluster EA7 phage Theresita is an exception. Theresita barely surpasses the threshold for inclusion in Cluster EA (it shares 37.5% shared gene content with Schubert) and nearly all of the genes are rightwards transcribed (Fig 5). All of the Cluster EA phages were isolated on *M*. *foliorum* except for Theresita, which was isolated on M. *natoriense* (Table 1). A genome map of a representative EA1 phage, TeddyBear, is shown in Fig 6A, and maps of other subcluster representatives are shown in S1–S7 Figs.

The Cluster EA phages have a canonical virion structure and assembly gene order common to phages with siphoviral morphologies (Figs 2, 5 and 6). There is variation among the tail genes and in the minor capsid MuF-like genes, the latter of which are unusually fused to the portal gene in Subclusters EA3, EA5, and EA8 (Figs 5 and S2, S4 and S7). All of the Cluster EA phages lack a capsid maturation protease gene, typically located between the portal and capsid subunit genes (Figs 5 and 6). Subcluster EA3, EA4, EA5, and EA6 phages all have a pair of tail assembly chaperone genes predicted to be expressed via a programmed translational frameshift, similar to lambda gpG and gpG-T [39], but the other Subcluster EA phages are atypical and the two ORFs appear to be separately expressed without an evident frameshift. Subclusters EA2 (except for Eleri), EA3, EA4, EA5, and EA8 encode a single tRNA-Ala (Figs 5 and 6 and S1–S7). Overall, the Cluster EA genome architectures (except for Subcluster EA7) are similar to those for Cluster A *Mycobacterium* and *Gordonia* phages, and *Rhodococcus* Cluster CA phages; the leftwards-transcribed right-arm genes include DNA Pol I, phosphoesterase, MazG-like protein, and thymidylate synthase, but the Cluster EA genes are very distantly related to those in Clusters A and CA. (Fig 5). In the Cluster EA phages the lysis cassette is downstream of the tail genes and includes a holin and an endolysin (Fig 6). Interestingly, the rightwards-transcribed right arm of Theresita is not closely related to the other Cluster EA phages (Fig 5), although the Theresita right arm has substantial similarity to Cluster EJ phages Goodman and Johann (Fig 6B). Theresita is thus an unusual hybrid of EA and EJ phages.

#### Cluster EB

**Fig 7:**
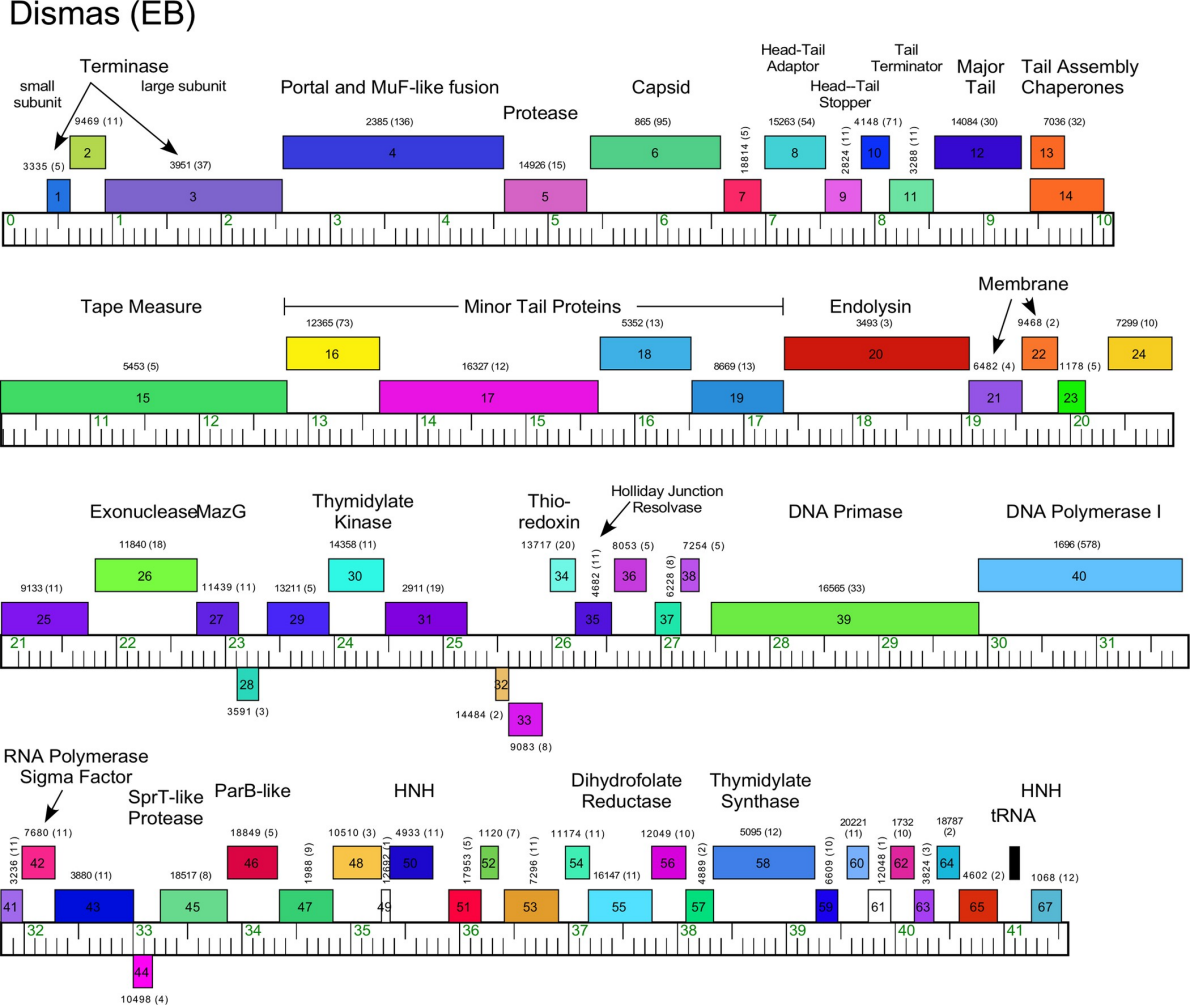
Genome organization of *Microbacterium* Cluster EB phage Dismas. See Fig 6A for details.

There are eleven Cluster EB phages who share 44–100% of their genes and whose pairwise ANI varies from 71–99%, and (S1 and S2 Tables and Figs 7 and S8). All Cluster EB phages were isolated on *M*. *foliorum*. With the exception of just 2–3 ORFs, all the genes are rightwards-transcribed and the virion structural genes are canonically organized—though all have fused MuF-like and portal function (Figs 7 and S8), like some Cluster EA phages. They also have one of two distinct tape measure protein genes whose lengths correlate with virion tail lengths as predicted (Fig 2) [40]. Curiously, there are short blocks of non-structural genes (e.g. Dismas *39*–*43*) that are conserved in all Cluster EB genomes, interspersed with highly variable regions (S8 Fig). The genomes have 1–3 tRNA genes at their extreme right ends (Table 1 and Figs 7 and S8). The lysis cassette is positioned downstream of the tail genes, and no integrase or repressor genes were identified (Figs 7 and S8), consistent with their lytic presentation.

#### Cluster EC

**Fig 8:**
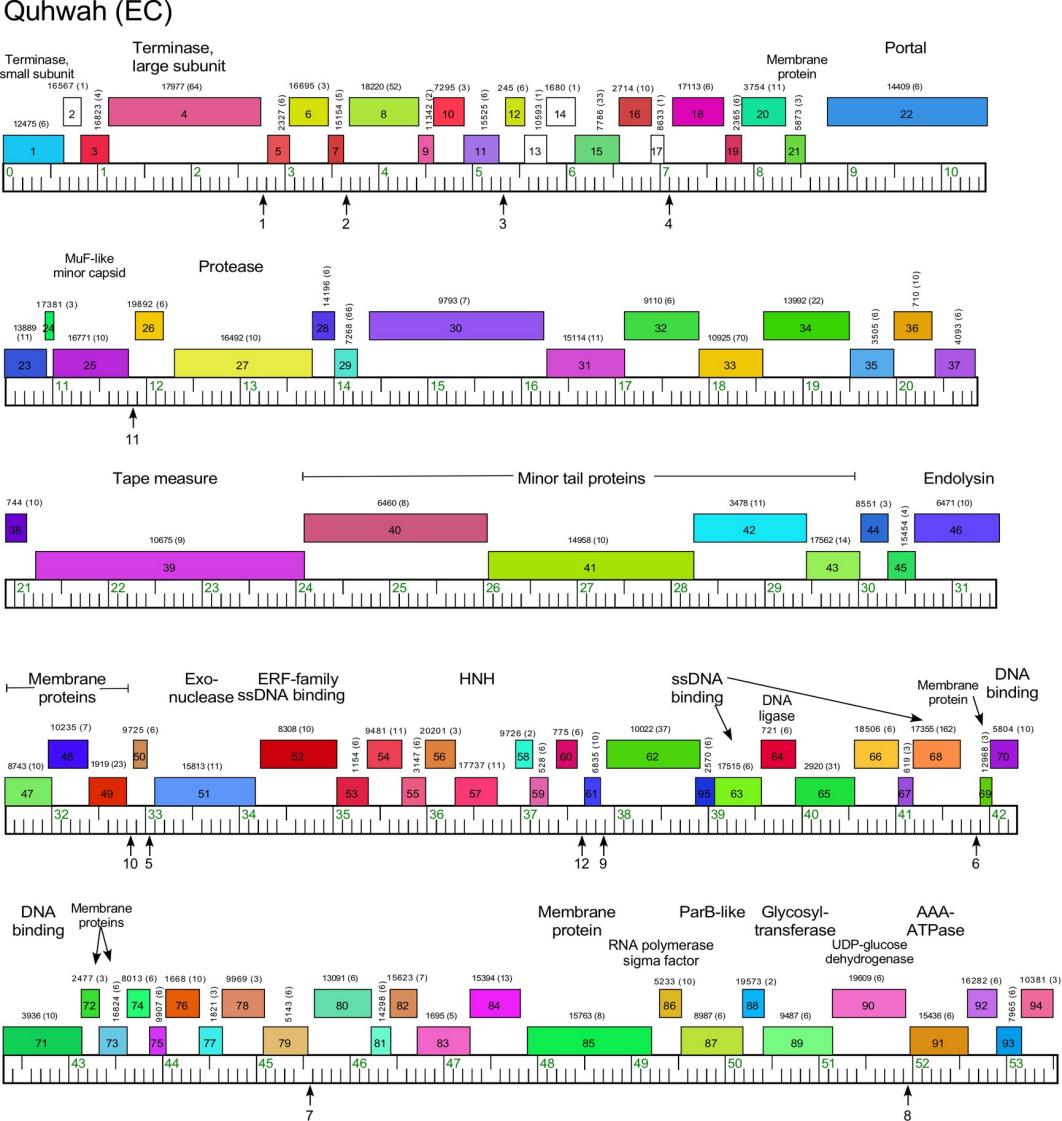
Genome organization of *Microbacterium* cluster EC phage Quahwah. See Fig 6A for details. Vertical arrows show the positions of 18 bp repeated motifs corresponding to the consensus 5’-TAGaCTCTaGGTgTaAgC, where upper case letters indicate complete conservation, and lower case letters are those appearing in at least 9 of the 12 instances. Each instance of the motif has no more than eight departures from the 18 bp consensus sequence and they are numbered as listed in S10A Fig.

The six Cluster EC phages were isolated on either *M*. *foliorum* or *M*. *paraoxydans* (Table 1) and share high pairwise ANI values (S1 Table). We note, however, that KaiHaiDragon, Paschalis and Quhwah, which were isolated on *M*. *foliorum* NRRL B-24224, and Fireman, Metamorphoo and RobsFeet isolated on *M*. *paraoxydans* NWU1, differ in a minor tail protein gene (e.g. Quhwah *40* and RobsFeet *38*, Figs 8 and S9), which may play a role in their host preferences (Figs 8 and S9). Consistent with this, phage RobsFeet, which was isolated on *M*. *paraoxydans* NWU1, does not efficiently infect *M*. *paraoxydans* NRRL B-14843 at the same efficiency (EOP of 10^−2^) (Table 2). All of the genes are rightwards-transcribed, and although the virion structure and assembly genes are canonically arranged, there are multiple small genes of unknown function interspersed between them. For example, in phage Quhwah, there are 17 contiguous ORFs between the terminase large subunit gene (*4*) and the portal gene (*22*), which are more typically adjacent to each other (Fig 8). The lysis cassette is positioned downstream of the tail genes, and the right arm genes include a RecET-like system (Quhwah *51* and *52*; Fig 8) and an RNA polymerase sigma factor that is likely involved in control of phage gene expression. There are also two glycosyltransferase genes and a UDP-glucose dehydrogenase gene (Fig 8). There are no repressor or integrase genes, consistent with their lytic properties.

**Table 2:** *Microbacterium* phage host ranges.

Phage	Cluster	Isolation host	M. fol^1^	M. nat^2^	M. aer^3^	M. tes^4^	M. hom^5^	M. par NRRL^6^	M. par NWU^7^	M. terrae
TeddyBear	EA1	M. fol NRRL B-24224	1	<10^−8^	10^−6^	10^−4^	<10^−8^	<10^−8^	<10^−8^	10^−2^
ColaCorta	EA2	M. fol NRRL B-24224	1	NT	<10^−7^	<10^−7^	<10^−7^	<10^−7^	<10^−7^	<10^−7^
Pajaza	EA3	M. fol NRRL B-24224	1	<10^−6^	<10^−6^	10^−3^	<10^−6^	10^−4^	<10^−6^	<10^−6^
Koji	EA4	M. fol NRRL B-24224	1	10^−7^	<10^−8^	10^−1^	<10^−8^	10^−4^	10^−2^	10^−3^
Neferthena	EA5	M. fol NRRL B-24224	1	NT	<10^−5^	<10^−5^	<10^−5^	<10^−5^	<10^−5^	<10^−5^
Chepli	EA6	M. fol NRRL B-24224	1	NT	<10^−5^	<10^−5^	<10^−5^	<10^−5^	<10^−5^	<10^−5^
Schubert	EA8	M. fol NRRL B-24224	1	<10^−6^	10^−3^	10^−3^	<10^−6^	<10^−6^	<10^−6^	10^−3^
Brahms	EB	M. fol NRRL B-24224	1	<10^−7^	10^−7^	<10^−7^	<10^−7^	<10^−7^	<10^−7^	<10^−7^
Musetta	ED2	M. fol NRRL B-24224	1	NT	<10^−9^	10^−6^	<10^−9^	<10^−9^	10^−8^	10^−3^
AnnaSerena	EF	M. fol NRRL B-24224	1	<10^−8^	<10^−8^	<10^−8^	<10^−8^	<10^−8^	<10^−8^	<10^−8^
Hyperion	EG	M. fol NRRL B-24224	1	<10^−8^	<10^−8^	<10^−9^	<10^−9^	<10^−9^	<10^−9^	<10^−9^
Percival	EH	M. fol NRRL B-24224	1	10^−5^	<10^−9^	<10^−9^	<10^−9^	10^−3^	10^−1^	10^−4^
Goodman	EJ	M. fol NRRL B-24224	1	<10^−9^	<10^−9^	10^−9^	<10^−9^	<10^−9^	<10^−9^	10^−3^
ArMaWen	EK1	M. fol NRRL B-24224	1	<10^−6^	<10^−6^	<10^−6^	<10^−6^	<10^−6^	<10^−6^	10^−4^
Akoni	EK2	M. fol NRRL B-24224	1	NT	10^−2^	<10^−5^	<10^−5^	<10^−5^	<10^−5^	<10^−5^
Appa	Sin	M. fol NRRL B-24224	1	<10^−8^	<10^−8^	<10^−8^	<10^−8^	<10^−8^	<10^−8^	<10^−8^
Hendrix	Sin	M. fol NRRL B-24224	1	<10^−5^	<10^−5^	<10^−5^	<10^−5^	<10^−5^	<10^−5^	<10^−5^
Triscuit	Sin	M. fol NRRL B-24224	1	<10^−7^	<10^−7^	<10^−7^	<10^−7^	<10^−7^	<10^−7^	<10^−7^
Camille	EL	M. aer NRRL B-24228	<10^−7^	<10^−7^	1	<10^−7^	<10^−7^	<10^−7^	<10^−7^	<10^−7^
Count	EL	M. aer NRRL B-24228	<10^−8^	<10^−8^	1	<10^−8^	<10^−8^	1	<10^−8^	10^−4^
Theresita	EA7	M. nat ATCC BAA1032	NT	1	NT	NT	NT	NT	NT	NT
RobsFeet	EC	M. par NWU1	<10^−9^	<10^−9^	10^−6^	<10^−9^	<10^−9^	10^−2^	1	<10^−9^
Hortus1	ED1	M. par NWU1	<10^−9^	<10^−9^	<10^−9^	<10^−9^	<10^−9^	<10^−9^	1	<10^−9^
MementoMori	EI	M. par NWU1	<10^−11^	<10^−11^	<10^−11^	<10^−11^	<10^−11^	<10^−11^	1	<10^−11^
BonaVitae	EE	M. par NWU1	<10^−9^	NT	10^−4^	<10^−9^	<10^−9^	<10^−9^	1	<10^−9^
Zeta1847	Sin	M. par NWU1	<10^−8^	<10^−8^	<10^−8^	<10^−8^	<10^−8^	<10^−8^	1	<10^−8^
Efeko^2^	EE	M. par NRRL B-14843	<10^−8^	NT	NT	NT	NT	1	NT	NT
Burro^2^	Sin	M. par NRRL B-14843	<10−^6^	<10−^6^	10^−5^	<10−^6^	<10−^6^	1	<10−^6^	<10−^6^
ValentiniPuff	Sin	M. par NRRL B-14843	<10^−7^	<10^−7^	10^−5^	<10^−7^	<10^−7^	1	<10^−7^	<10^−7^

Table shows efficiencies of plating relative to infection of the host used for isolation

NT: Not Tested

^1^M. fol: *Microbacterium foliorum* NRRL B-24224

^2^M. nat: *Microbacterium natoriense* ATCC BAA-1032

^3^M. aer: *Microbacterium aerolatum* NRRL B-24228

^4^M. tes.: *M*. *testaceum*

^5^M. hom: *M*. *hominis*

^6^Mpa NRRL B-14843: *Microbacterium paraoxydans* NRRL B-14843

^7^Mpa NWU1: *Microbacterium paraoxydans* NWU1

An intriguing feature of the Cluster EC genomes is an 18 bp asymmetric sequence motif (5’-TAGaCTATaGGTgTaAgC; see S10A Fig) repeated 12 times in each genome positioned in small intergenic regions (Fig 8). Seven of these are located among the non-structural genes in the right part of the genome, four are among the set of genes present between the terminase and portal genes, and one is located upstream of *26*, a gene of unknown function inserted between the MuF-like minor capsid (*25*) and the capsid maturation protease gene (*27*, Fig 8). All are oriented similarly and positioned 21–30 bp upstream of a predicted translation initiation codon, and upstream of the putative ribosome binding sequence. It is unclear if these motifs are involved in regulation of gene expression or another aspect of lytic growth.

### Cluster ED

**Fig 9:**
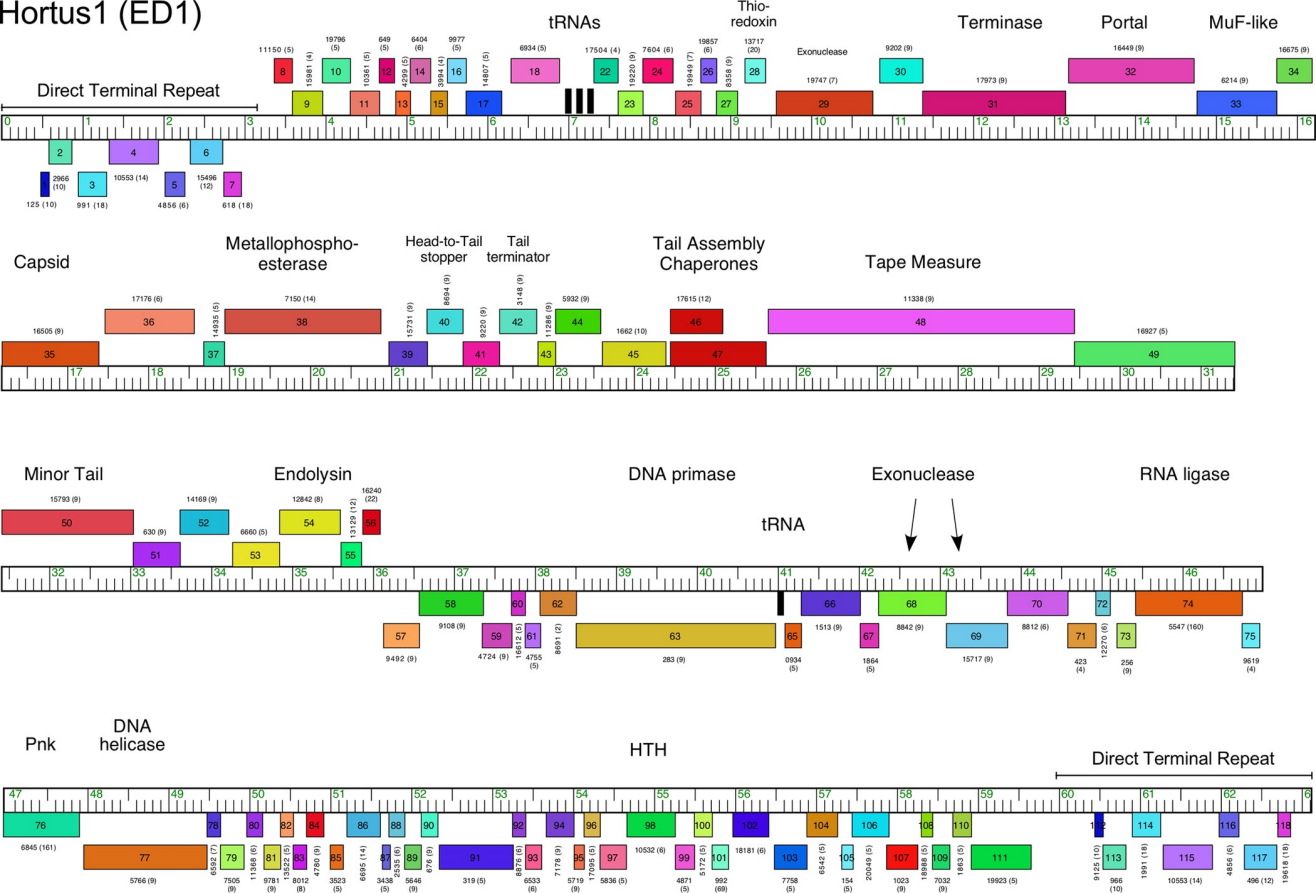
Genome organization of *Microbacterium* Subcluster ED1 phage Hortus1. See Fig 6A for details.

The nine Cluster ED genomes are organized into two subclusters (ED1 and ED2; Table 1 and Figs 9 and S11). The three Subcluster ED2 phages were isolated on *M*. *foliorum* NRRL B-24224, whereas five of the Subcluster ED1 phages were isolated on *M*. *paraoxydans* NWU1 and one (Jacko) was isolated on *M*. *paraoxydans* NRRL B-14843 (Table 1). Most of the Subcluster ED1 phages are closely related (88–94% pairwise shared gene content), although Jacko is more distantly related (52–54% shared gene content with the other phages). The Subcluster ED2 phages share 82–86% gene content with each other, and 33–36% with the ED1 phages.

The genome architectures of these phages are reminiscent of *Arthrobacter* Cluster AQ phages [3], although they share no similarity at the nucleotide or protein sequence levels. They have long DTRs (3,159 bp) encoding seven leftwards-transcribed genes, and a set of ~20 rightwards-transcribed genes (including 2–4 tRNA genes) between the DTR and the rightwards-transcribed virion structural genes (Figs 9 and S11). There are also several genes inserted between the capsid subunit gene (e.g. Hortus1 *35*) and the head-to-tail connector genes (Figs 9 and S11), interrupting the canonical siphoviral genome organization. The non-structural genes in the right part of the genome are all leftwards-transcribed (Figs 9 and S11), and include RNA ligase (e.g. Hortus1 *74*) and polynucleotide kinase (e.g. Hortus1 *76*) genes which may be involved in countering RNA cleavage-mediated host defense systems (Figs 9 and S11) [41]. No repressor or integrase genes were identified.

#### Cluster EE

**Fig 10:**
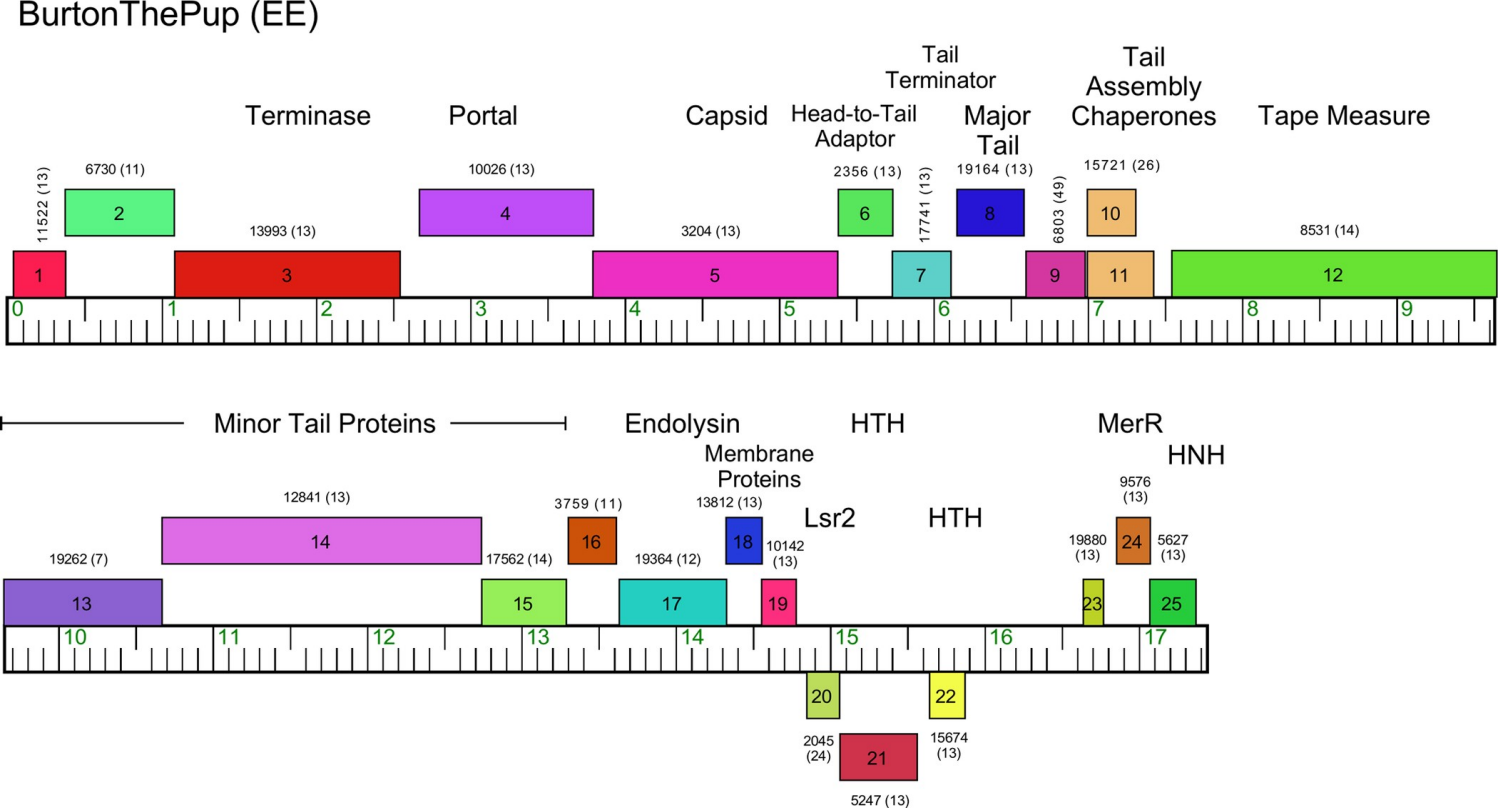
Genome organization of *Microbacterium* Cluster EE phage BurtonThePup. See Fig 6A for details.

The thirteen Cluster EE phages are among the smallest actinobacteriophage genomes (17,032 to 17,534 bp; the actinobacteriophage average is 61.6 kbp) (Table 1 and Figs 10 and S12). They are very closely related to each other, with some variation in the rightmost parts of their genomes (S12 Fig), but lack many of the non-structural genes found in larger genomes. A particularly unusual feature of the virion structural genes is the fusion of the capsid maturation protease, scaffolding, and the HK97-like capsid subunit into a single gene (e.g. BurtonThePup *5*; Fig 10). Phages Efeko and BonaeVitae were isolated on *M*. *paraoxydans* NRRL B-14843 and NWU1, respectively, and have substitutions for some tail genes relative to the others that were isolated on *M*. *foliorum* NRRL B-24224 (Table 1); these phages show strong preferences for infection of the strains on which they were isolated (Table 2). Overall, the virion structure and assembly genes are compacted into less than 14 kbp of the genomes, and the remaining 4 kbp of the genomes contains a lysis cassette, several putative transcriptional regulators (including *lsr2*), and an HNH nuclease (Figs 10 and S12). *lsr2* is also found in a number of mycobacteriophage genomes [42]. The genomes lack integrase or repressor genes, which is consistent with their lytic properties.

Cluster EE genome architecture is similar to that of the small-genome phages isolated on *Arthrobacter* (Clusters AN, AX, and FE), *Gordonia* (Clusters CW and DM), and *Rhodococcus* (singleton phage RRH1) [2, 3, 43], although they share little sequence similarity to one another. Interestingly, all of these also have a protease-scaffold-capsid fusion, an apparent common feature of these siphoviruses with uncommonly small genomes.

#### Cluster EF

**Fig 11:**
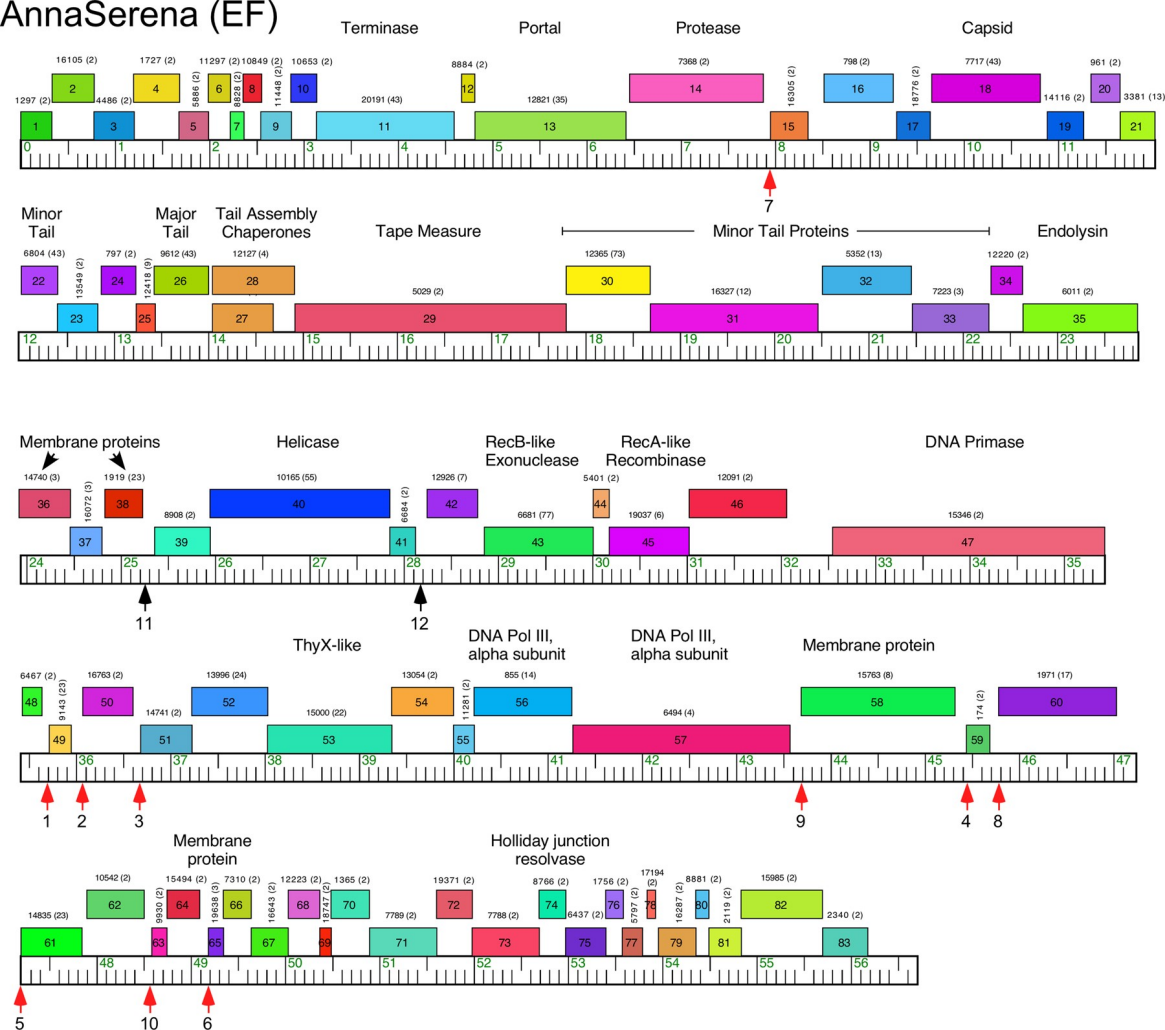
Genome organization of *Microbacterium* cluster EF phage AnnaSerena, See Fig 6A for details. The small red vertical arrows indicate the locations containing both conserved SAS and ESAS sequences; black arrows indicate the locations with an ESAS motif that lack an associated SAS. The SAS motifs are numbered as listed in S10B and S10C Fig.

There are two Cluster EF phages—AnnaSerena and Krampus—and they are very closely related, differing by only ~500 single nucleotide polymorphisms (SNPs) and short insertion/deletions. They share the same gene content. All genes are rightwards-transcribed, and the virion structure and assembly genes are canonically organized in the left parts of the genomes, albeit with some additional gene(s) inserted between the terminase (*11*) and portal (*13*) genes, as well as the protease (*14*) and capsid subunit genes (*18*) (Fig 11). The lysis cassette is located downstream of the tail genes. Non-structural genes in the right arms include a DNA Primase, a RecA-like protein, ThyX, and a Holliday Junction Resolvase. They also have two genes related to a DNA Polymerase III alpha subunit, which are typically expressed from a single gene. No repressor or integrase genes were identified.

The Cluster EF genomes contain ten copies of a 13 bp sequence motif (with no more than one mismatch; consensus, 5’-GGGAAAGGACCCC) positioned upstream of some predicted translational start codons (Figs 11 and S10B). The motifs are located at the positions of the ribosome binding sites but are unusually well-conserved, reminiscent of the Start Associated Sequences (SAS) in Cluster K mycobacteriophages [44]. Moreover, all of these are linked to a weakly conserved sequence immediately upstream in the non-coding intergenic gaps, mirroring the Extended Start Associated Sequences (ESAS) in the Cluster K phages [44]. There is little sequence similarity between the Cluster K and EF genomes, or the conserved motifs, and it is unclear what roles these play in the regulation of gene expression, although their conservation within clusters suggests they are functionally important.

#### Cluster EG

**Fig 12:**
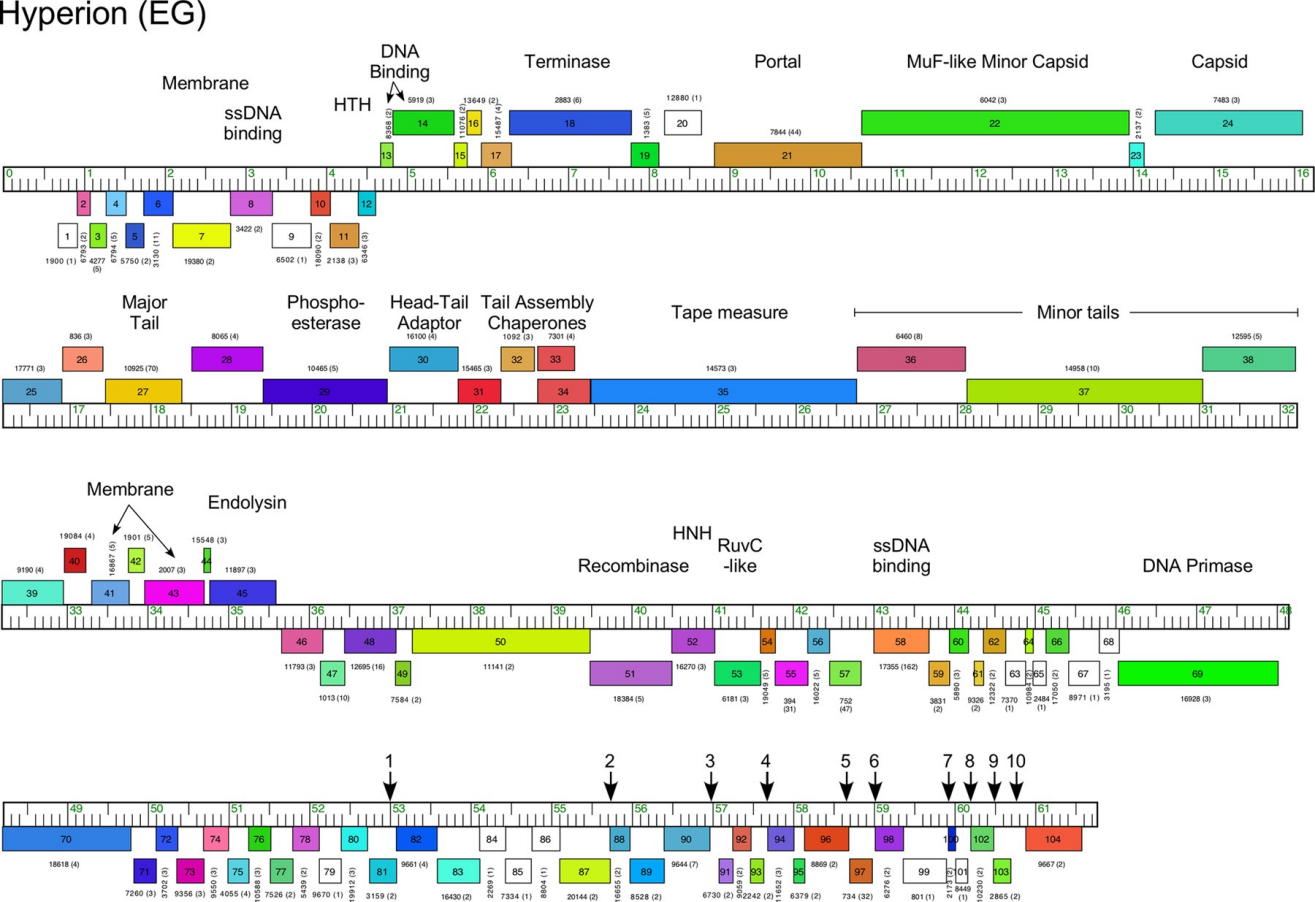
Genome organization of *Microbacterium* cluster EG phage hyperion. See Fig 6A for details. Vertical arrows indicate the positions of conserved short inverted repeat sequences and numbered as listed in S10D Fig.

The three Cluster EG phages are quite diverse with 50–80% pairwise average gene content (Figs 12 and S13A), and the right parts of the genomes are the most varied (S13 Fig). The genomes have short (200bp) DTRs, with the virion structure and assembly genes rightwards-transcribed in the left parts of the genomes, and non-structural genes are leftwards-transcribed in the right part of the genome as well as the left end of genome (between the DTR and the terminase gene) (Figs 12 and S13A). The structural genes are mostly canonically organized, but the minor capsid MuF-like protein is fused to the capsid maturation protease in a single gene, and the major tail protein gene is atypically located upstream of some of the head-to-tail connector genes. The lysis cassette is located downstream of the tail genes (Fig 12). No repressor or integrase genes were identified.

The Cluster EG phages have 8–10 instances of an 18-bp inverted repeat located in short intergenic regions, typically 14–22 bp upstream of translation initiation codons of genes at the right ends of the genomes (Figs 12 and S10D and S13B). These are similar in organization to the 17 bp inverted repeats in the Cluster O mycobacteriophages [45], although the sequences are different. Interestingly, these are conserved in the three Cluster EG phages, even though the region at the right end of the OneinaGillian genome containing these motifs is quite different from Hyperion and Squash (S13B Fig).

#### Cluster EH

**Fig 13:**
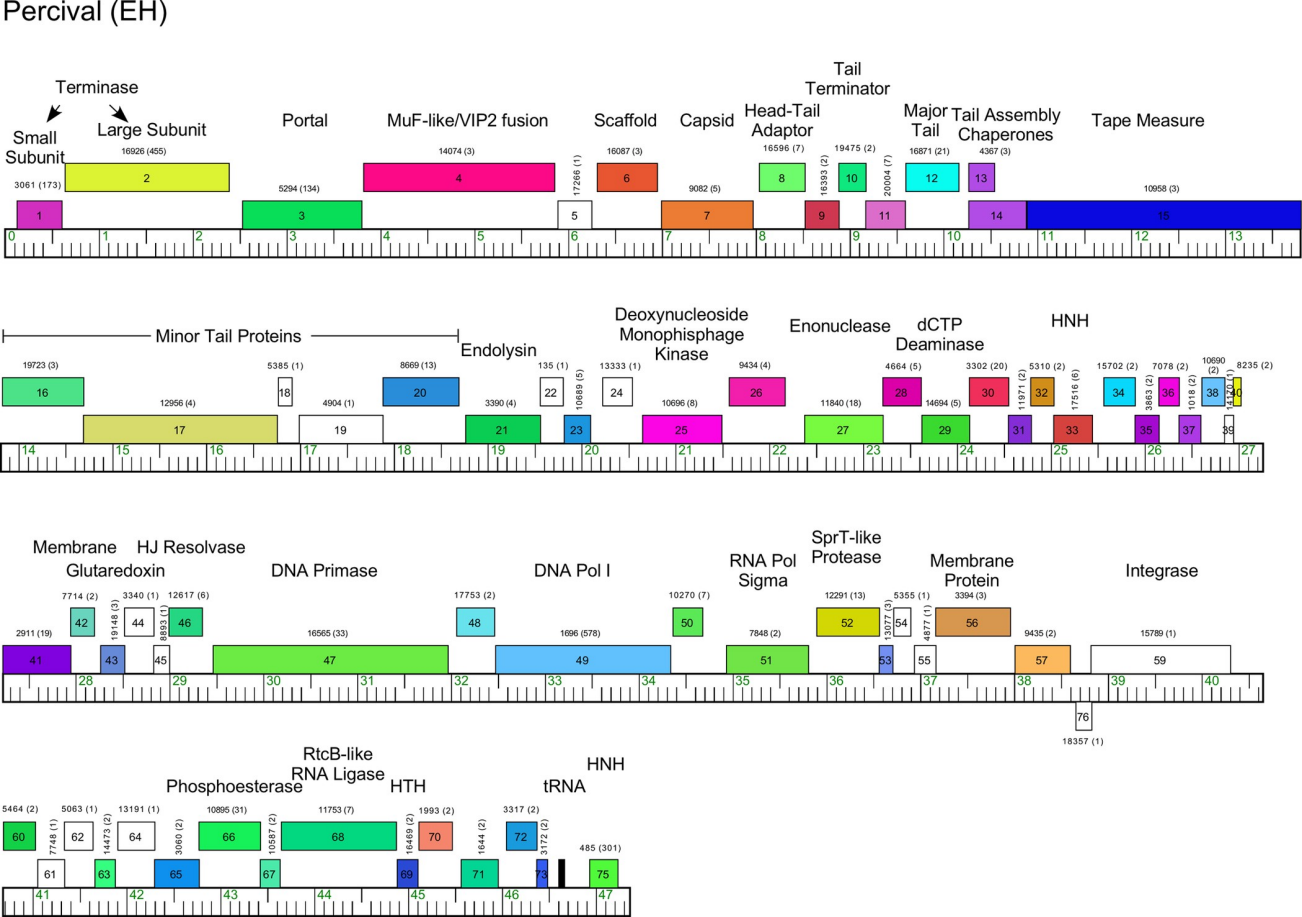
Genome organization of *Microbacterium* cluster EH phage percival. See Fig 6A for details.

The two Cluster EH phages, Percival and Floof, share 69% of their gene content and all of the genes are rightwards-transcribed, with the exception of Percival *76* (Figs 13 and S14). The virion structure and assembly genes are canonically organized, but Percival gene *4* codes for a fusion of a MuF-like minor capsid protein with a VIP2-like ADP-ribosyltransferase toxin; the Floof homologue lacks the VIP2 function (S14 Fig). VIP2-like toxin genes are encoded by some other actinobacteriophages including phages in Clusters A (Subclusters A2 and A15) and D. The role of this VIP2 toxin is unclear, but it is notable that in Percival it is predicted to be a component of the virion. The lysis cassette follows the tail genes, and the non-structural genes include DNA primase, DNA polymerase, RtcB-like RNA ligase, and a Holliday Junction Resolvase (Figs 13 and S14). The Cluster EH phages do not have a repressor gene and the organization is distinct from many temperate phages. Percival and Floof do, however, have distantly-related serine-integrase genes located near the right genome ends (Figs 13 and S14). It is unclear if these reflect a temperate nature of the phages, or if they play an alternative role in lytic growth.

#### Cluster EI

**Fig 14:**
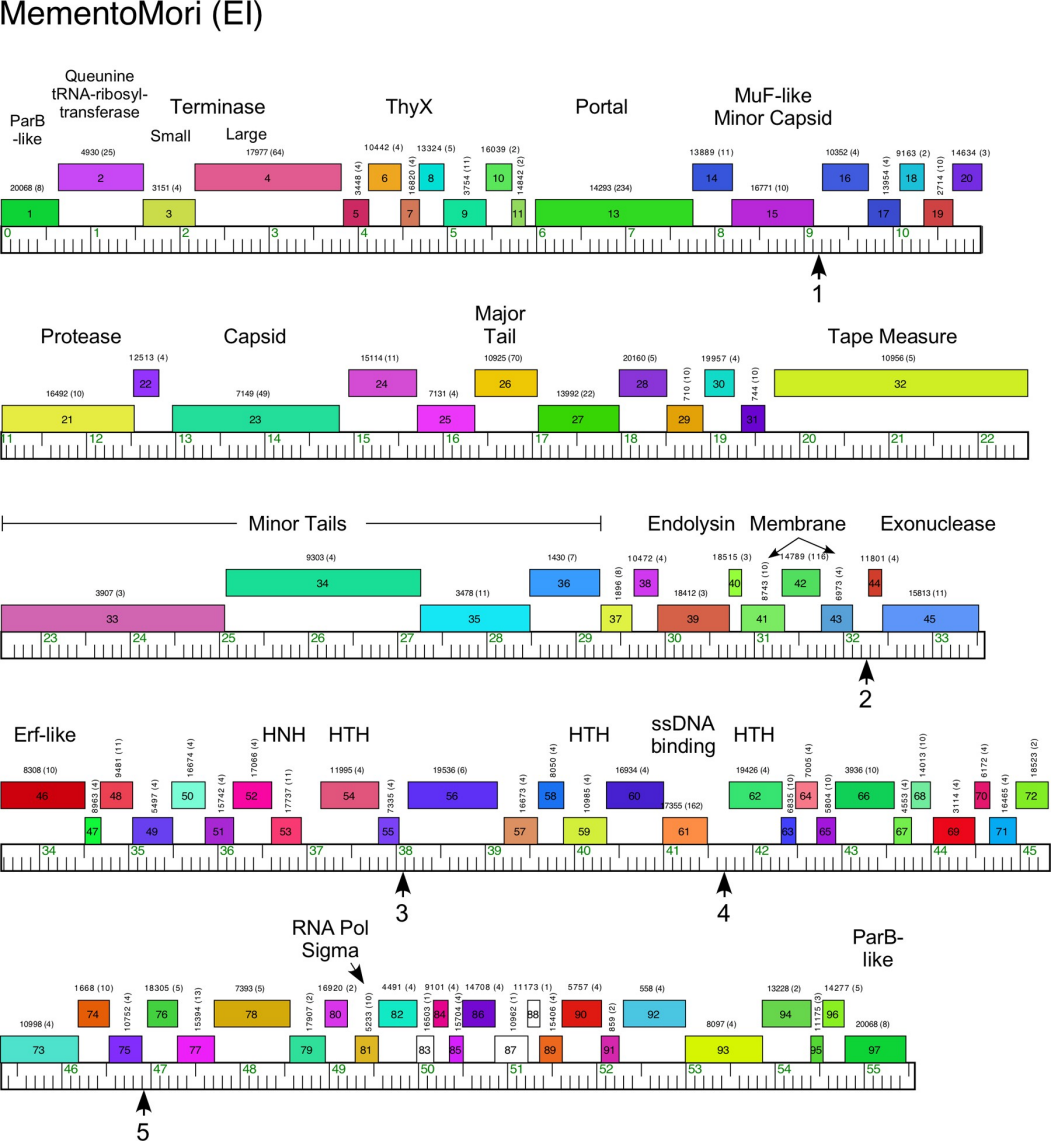
Genome organization of *Microbacterium* cluster EI phage MementoMori. See Fig 6A for details. Vertical arrows indicate the locations of 30 bp repeated sequences in intergenic regions, numbered as shown in S10E.

The four Cluster EI phages are closely related to each other (80–92% shared gene content) and are composed solely of rightwards-transcribed genes (Figs 14 and S15). They are also related to Cluster EC phages with relatively high shared gene contents (24–28%, S2 Table) but below the threshold for inclusion in the same cluster (35%). The virion structure and assembly operons have several unusual gene insertions including the regions between the terminase subunit and portal genes, between the protease and capsid genes, and between the major tail subunit and tape measure protein genes (Figs 14 and S15). There are two adjacent genes (*1*, *97*) coding for ParB-like proteins but with only 40% aa identity to each other (although displayed at the ends of the circularly permuted genomes (Table 1) when linearized for visualization purposes, Figs 14 and S15). The non-structural genes include several putative DNA-binding proteins (e.g. MementoMori *54*, *59*, *62*, Fig 14), an RNA polymerase sigma factor gene and an Erf-family recombination system (*45*, *46*). There are five copies of a repeated sequence motif in short intergenic regions similar to those described for Cluster EC phages above, although their functions are not known (Figs 14 and S10). No repressor or integrase genes were identified.

#### Cluster EJ

**Fig 15:**
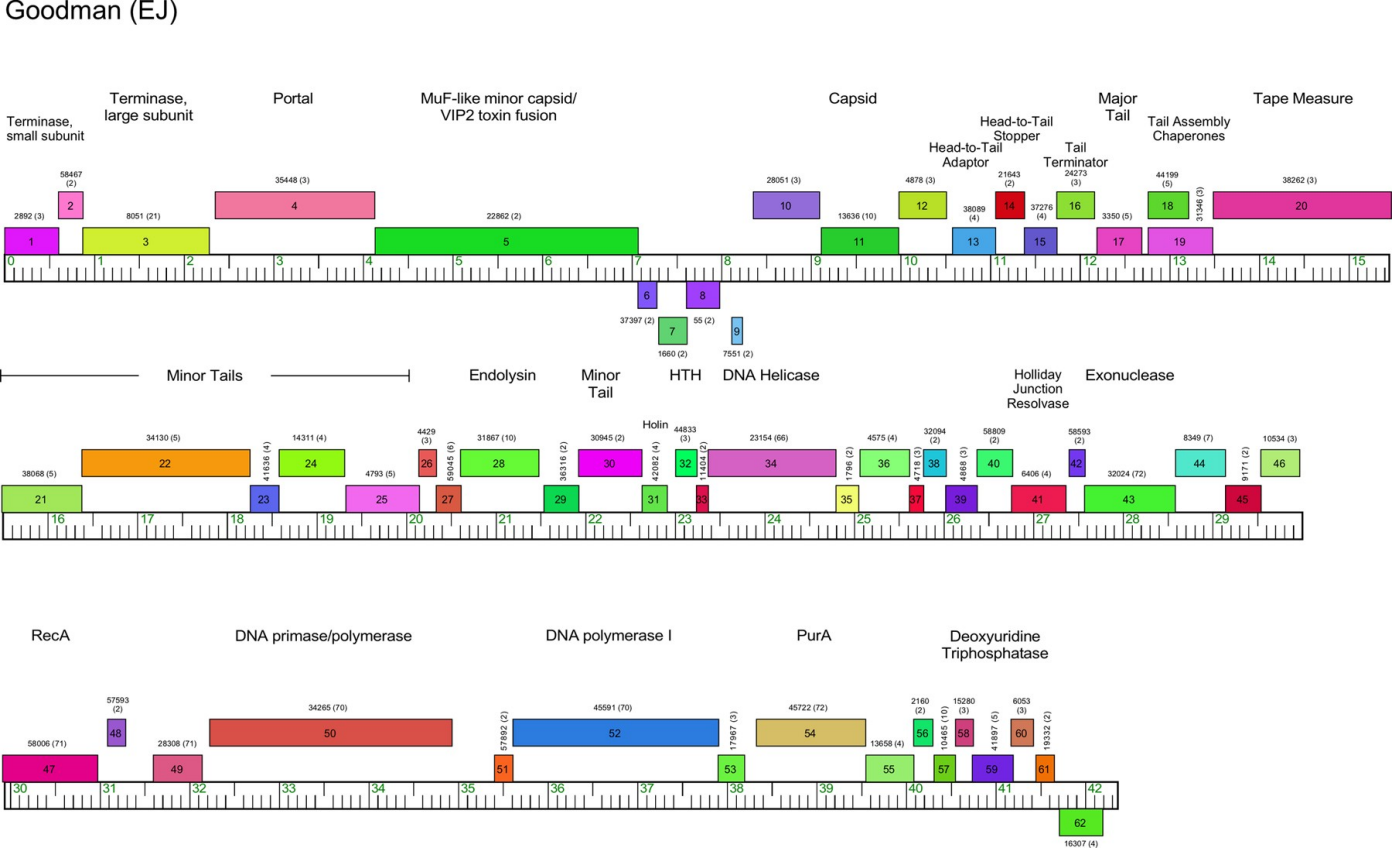
Genome organization of *Microbacterium* Cluster EJ phage Goodman. See Fig 6A for details.

The two Cluster EJ phages, Goodman and Johann, are very closely related with the same gene content (Figs 15 and S16). Most of the genes are rightwards-transcribed, although there is a group of four leftwards-transcribed genes inside the structural gene operon. Both phages contain a gene that fuses a MuF-like protein and VIP2 ADP-ribosyltransferase toxin function, as described above for Cluster EH phages (Figs 15 and S16). The Cluster EJ phages code for both a DNA Polymerase I and a DNA primase/polymerase as well as RecA. There are no features of temperate phages, consistent with their lytic properties.

#### Cluster EK and the singleton phage Burro

**Fig 16:**
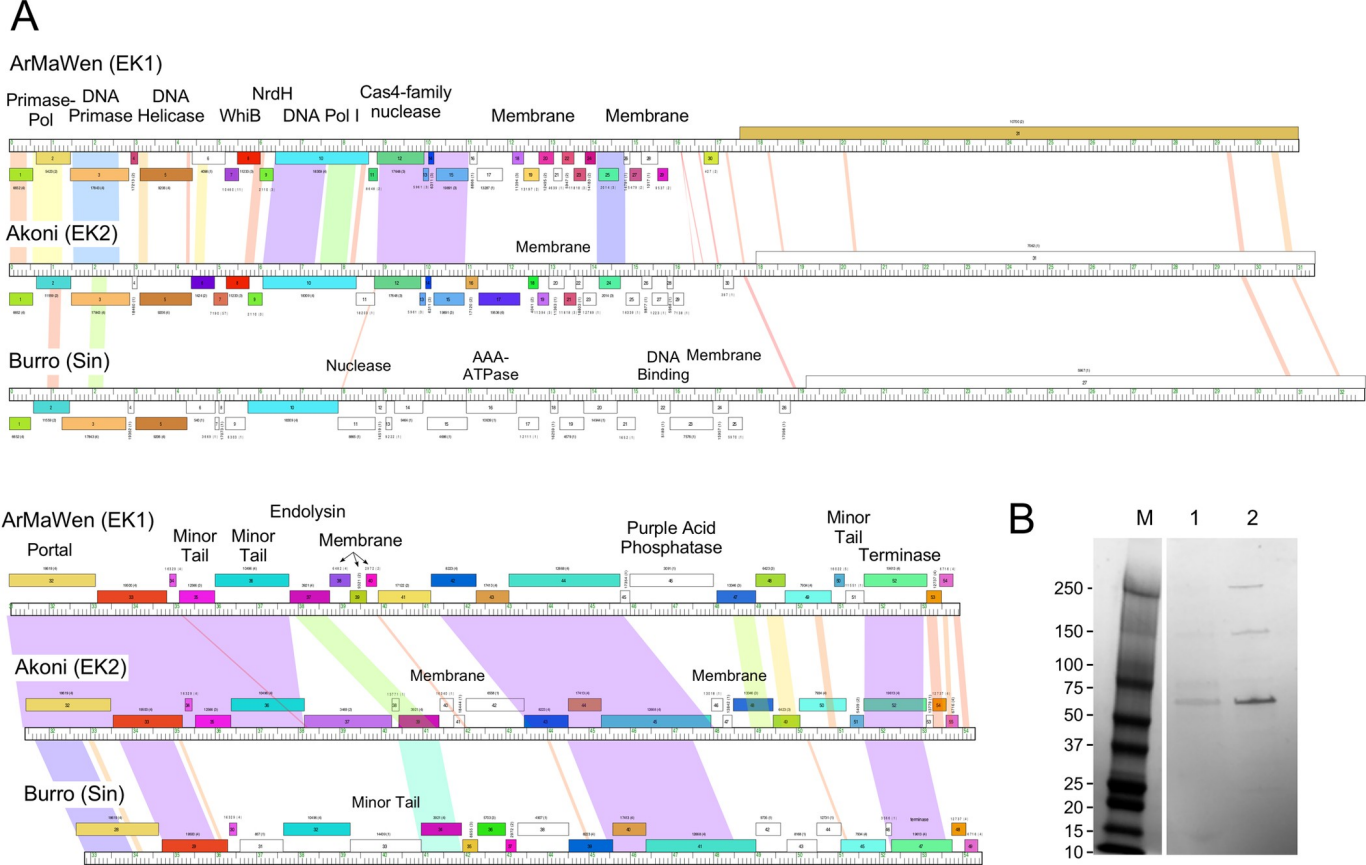
*Microbacterium* cluster EK phages and singleton burro. **A.** Genome organization of phages ArMaWen, Akoni and Burro, clusters EK1, EK2, and a singleton, respectively. See Fig 5 for details. **B**. **SDS-PAGE of Burro virions.** Lanes 1 and 2 correspond to Burro virions purified through one and two CsCl density gradients, respectively. M, marker with protein sizes indicated in kDa.

The three Cluster EK phages, ArMaWen, TinyTimothy, and Akoni, are grouped into two subclusters EK1 and EK2 (Table 1 and Fig 16A). Singleton Burro does not meet the threshold of similarity for inclusion in Cluster EK but shares several features and we will discuss them together. All have podoviral morphologies (Fig 2) and similar genome architectures. The genes are organized into leftwards- and rightwards-transcribed groups (e.g. ArMaWen *1*–*30*, and *31*–*54*, respectively) with virion structure and assembly genes in the rightwards-transcribed group. No repressor or integrase genes were identified. The portal protein gene is strongly predicted to be Burro *28* (and its homologues), but the location of the capsid subunit gene is unclear. The most obvious candidate is the adjacent gene (e.g. Burro *29* and its relatives) although it has no discernible bioinformatic features of capsid proteins. However, purified Burro virions contain an abundant protein of ~57 kDa (Fig 16B), consistent with the capsid subunit being Burro gp29 (predicted to be 57.9 kDa).

The most striking feature of these phages is a very large gene spanning more than 13 kbp, representing almost 25% of the entire genome. The predicted gene products are more than 4,400 residues long and are the largest in any actinobacteriophage genome, and among the largest in any viral genome described to date. Moreover, they are highly divergent in sequence, and Burro gp27 and Akoni gp31 share less than 30% amino acid identity with each other, or with either ArMaWen (Fig 16) or TinyTimothy gp31. However, the roles of the proteins are unclear. They contain few informative conserved domains, although Burro gp27 has two predicted transmembrane domains near its N-terminus while the other phage’s proteins do not. Notably, they do not show significant sequence similarity or share motifs with the viral RNA polymerases of *Enterobacteriaceae* phage N4 and its relatives, which are also very large (3,500-residues) [46]. Burro virions contain a large protein (~250 kDa), which is bigger than any gene product predicted in the Burro genome, and presumably corresponds to gp27 or a processed part of it. Burro gp27 is thus virion associated, although its specific role is not known.

#### Cluster EL

**Fig 17:**
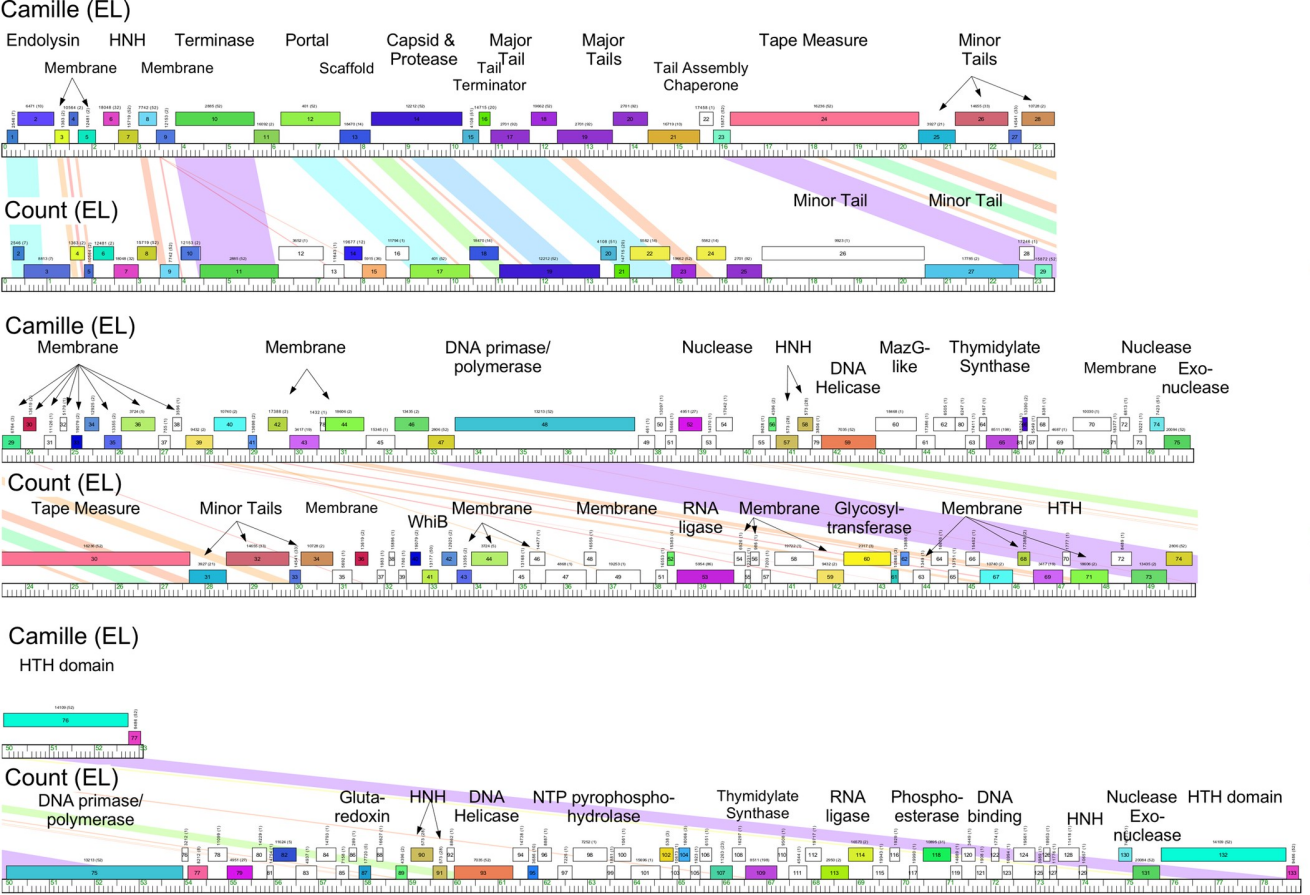
Genome organization of *Microbacterium* Cluster EL phages, Camille and Count. See Fig 5 for details.

The two Cluster EL phages, Count and Camille, have 41% shared gene content and differ substantially in genome length (78,922 bp and 53,097 bp, respectively). Many of the shared genes are located in the left arm and code for virion structure and assembly functions, including a fusion of the capsid maturation protease and the capsid subunit into a single protein (Fig 17). These proteins differ somewhat between the two genomes (~50% aa identity) but are of interest as the two phages have different virion morphologies, with Camille having an isometric capsid, and Count having a prolate capsid (Fig 2). It is plausible that the Count protease/capsids diverged with distinct morphologies specifically to accommodate different genome sizes. Count contains a high proportion of orpham genes (those with no close relatives), which largely account for the difference in genome lengths. All genes are rightwards-transcribed in both phages. No repressor or integrase genes were identified. These phages have the lowest G+C% contents of the *Microbacterium* phages (51.4% and 56.3% for Count and Camille, respectively), and for both the right one-third of the genomes has a modestly lower G+C% content than the left two-thirds (e.g. Count 1–49.5 kbp, 52.6%; 49.5–78.9 kbp, 49.3%). The G+C% contents are substantially lower than their *M*. *aerolatum* host (69.3%) [47]; this mismatch suggests they may have acquired the ability to infect the host relatively recently in their evolutionary history, as proposed for mycobacteriophage Patience [48].

#### Singletons Appa, Hendrix, Triscuit, ValentiniPuff, and Zeta1847

**Fig 18:**
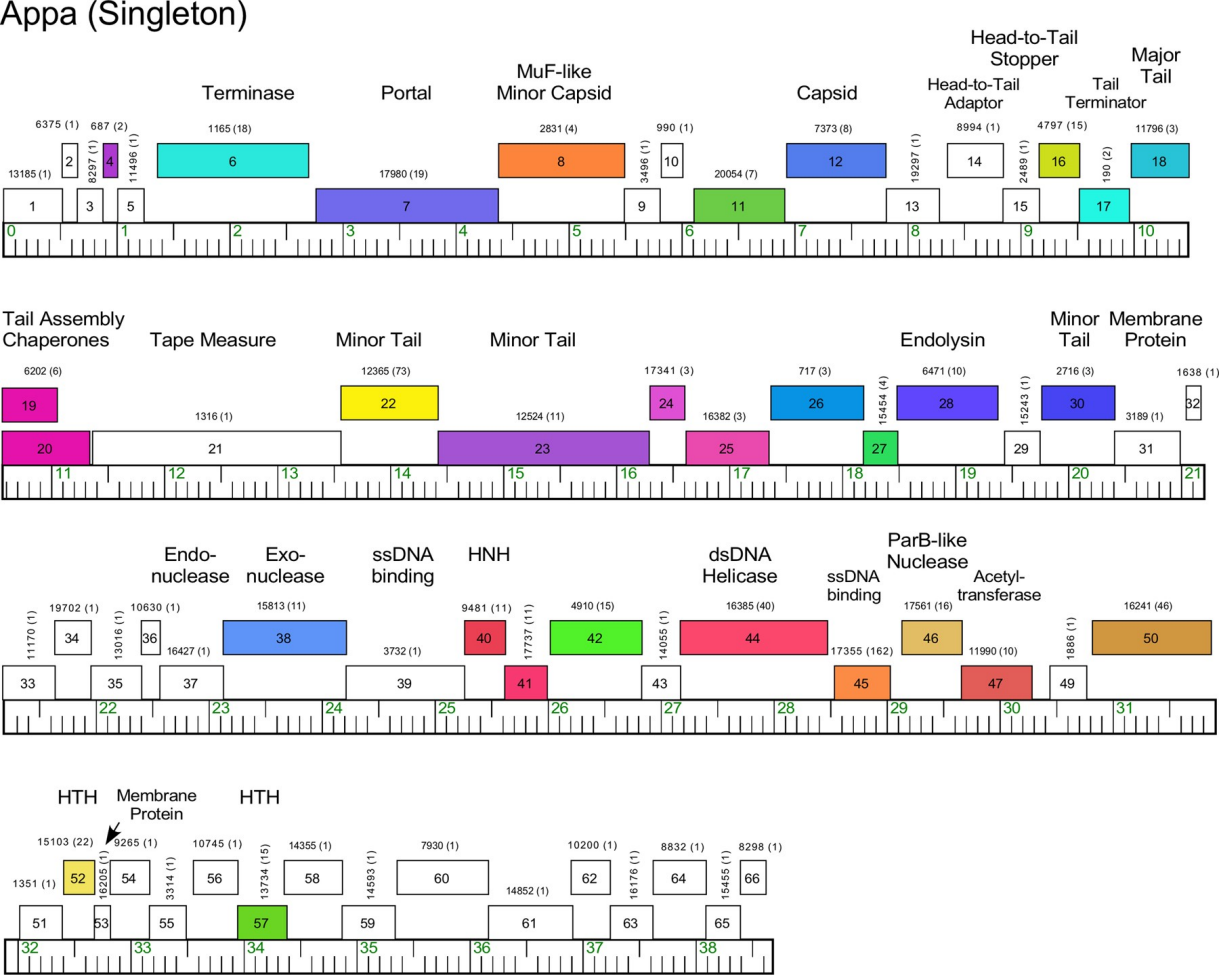
Genome organization of *Microbacterium* Singleton phage Appa. See Fig 6A for details.

**Fig 19:**
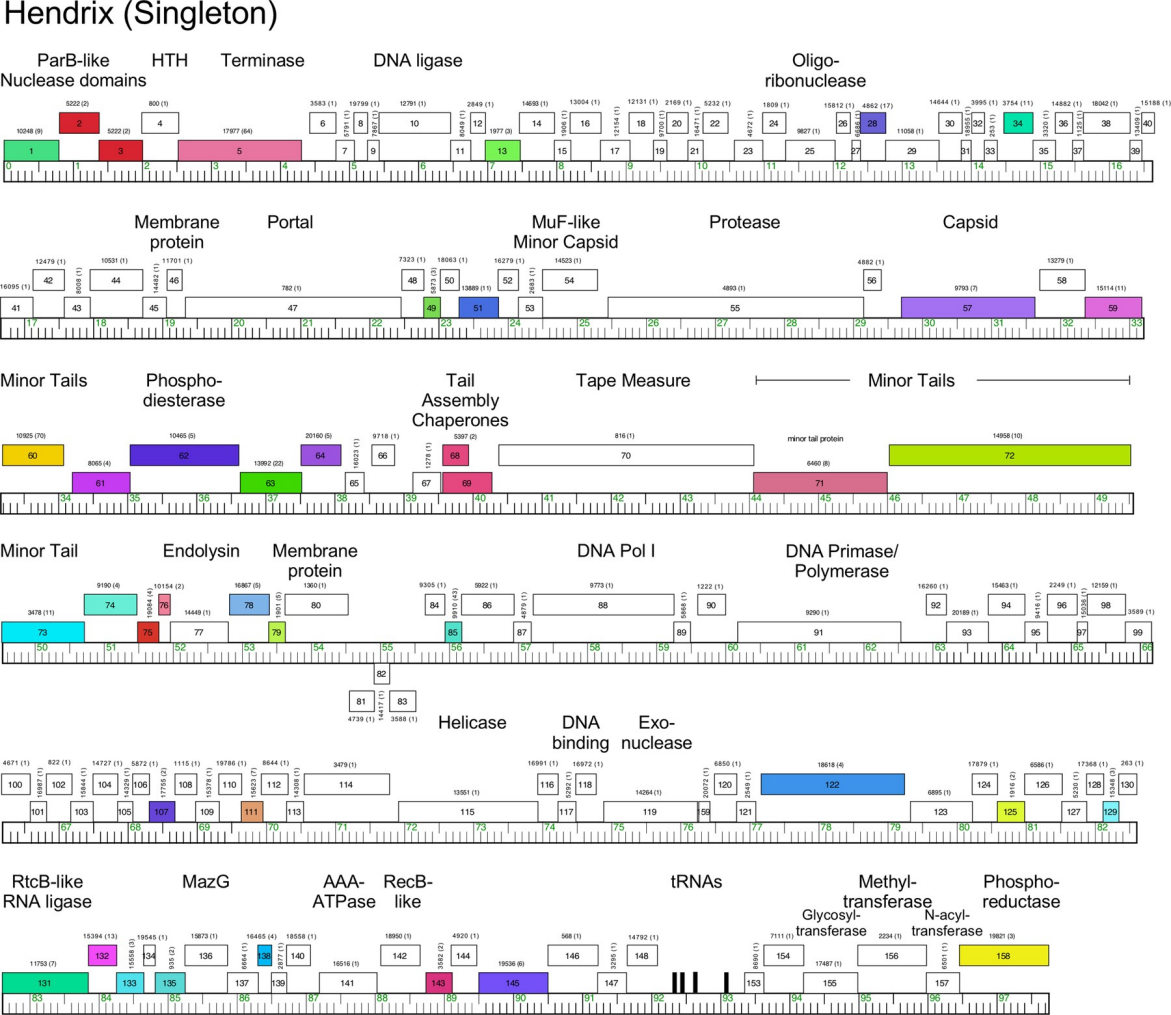
Genome organization of *Microbacterium* Singleton phage Hendrix. See Fig 6A for details.

**Fig 20:**
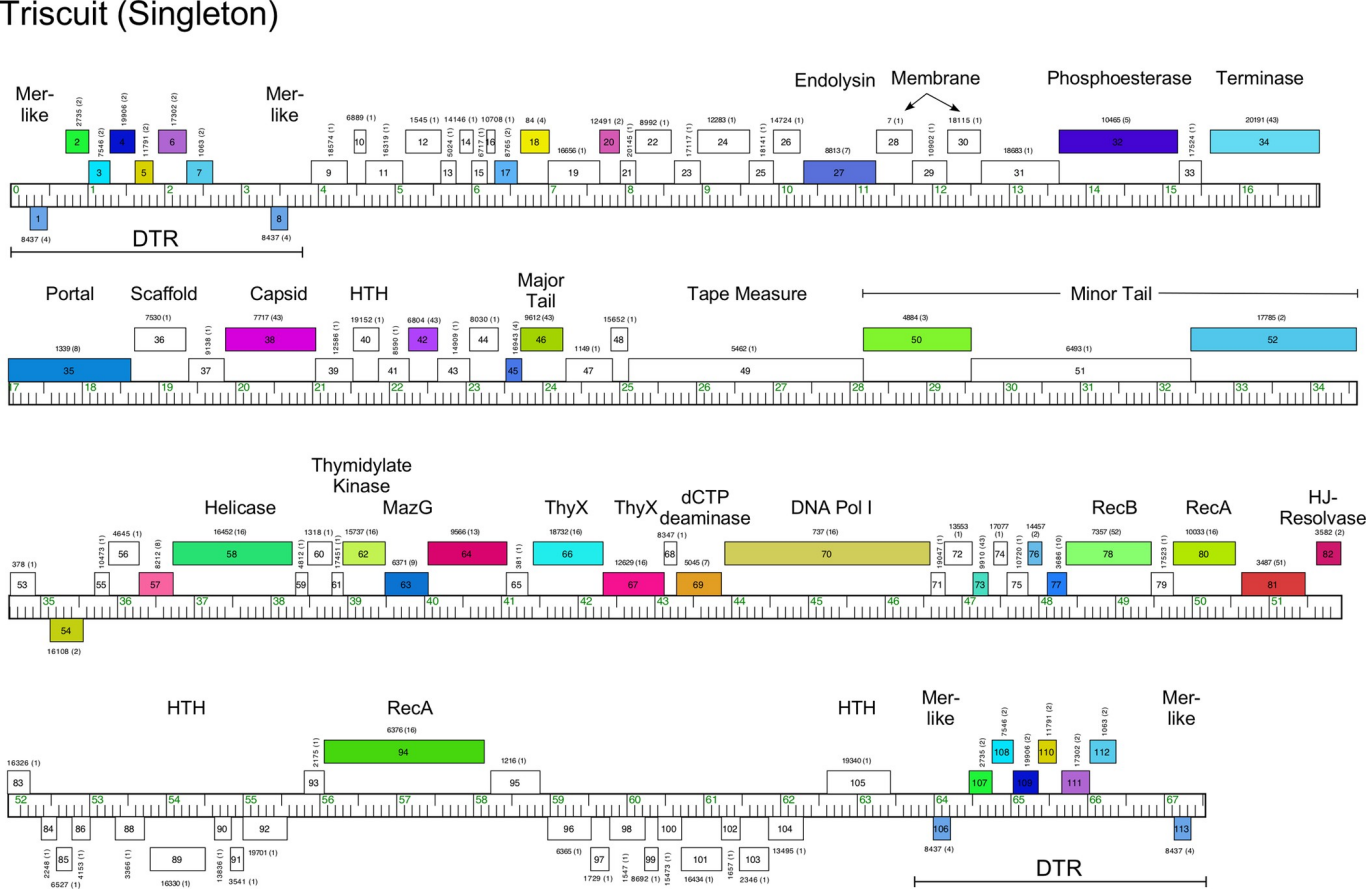
Genome organization of *Microbacterium* Singleton phage Triscuit. See Fig 6A for details.

**Fig 21:**
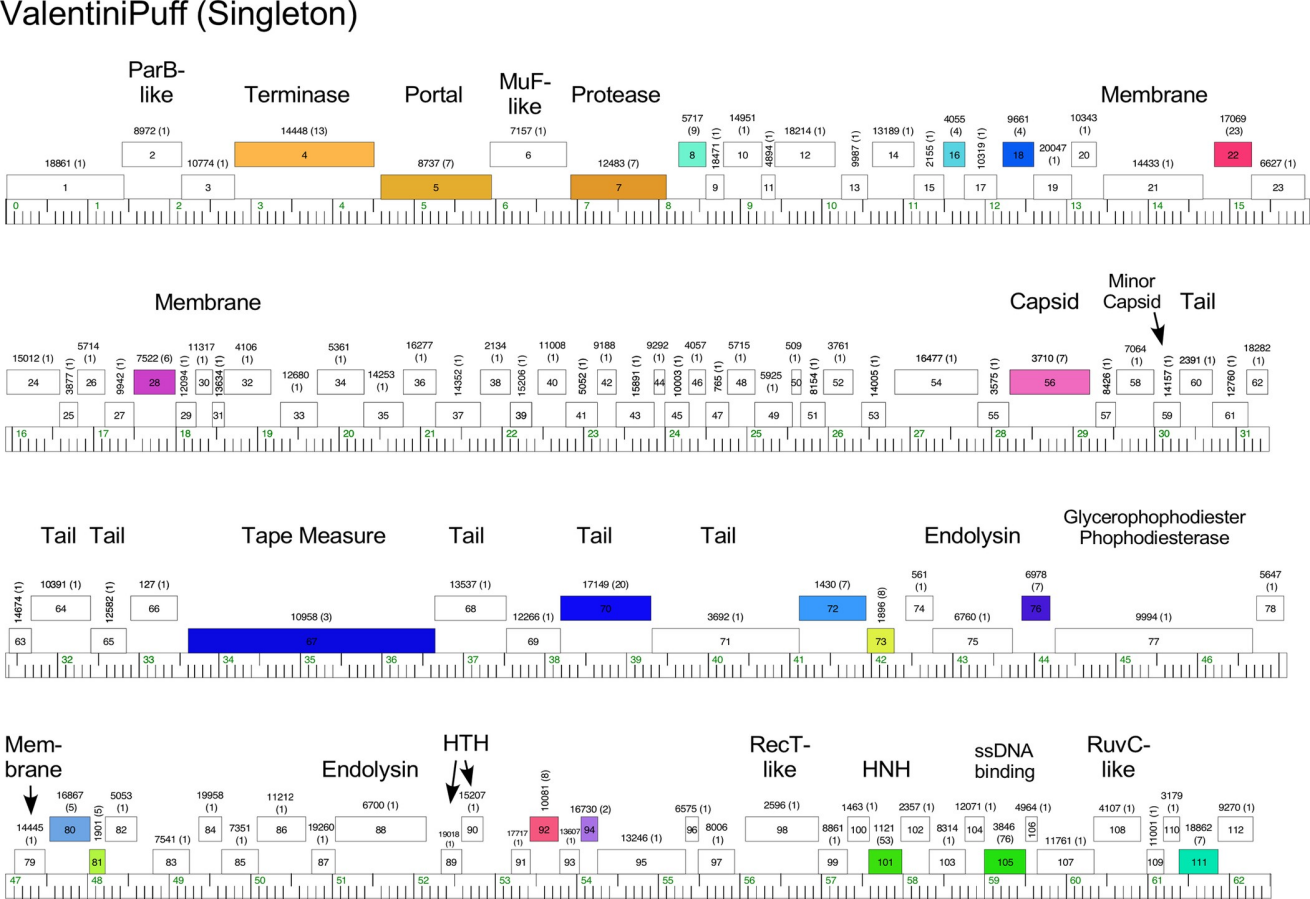
Genome organization of *Microbacterium* Singleton phage ValentiniPuff. See Fig 6A for details.

**Fig 22:**
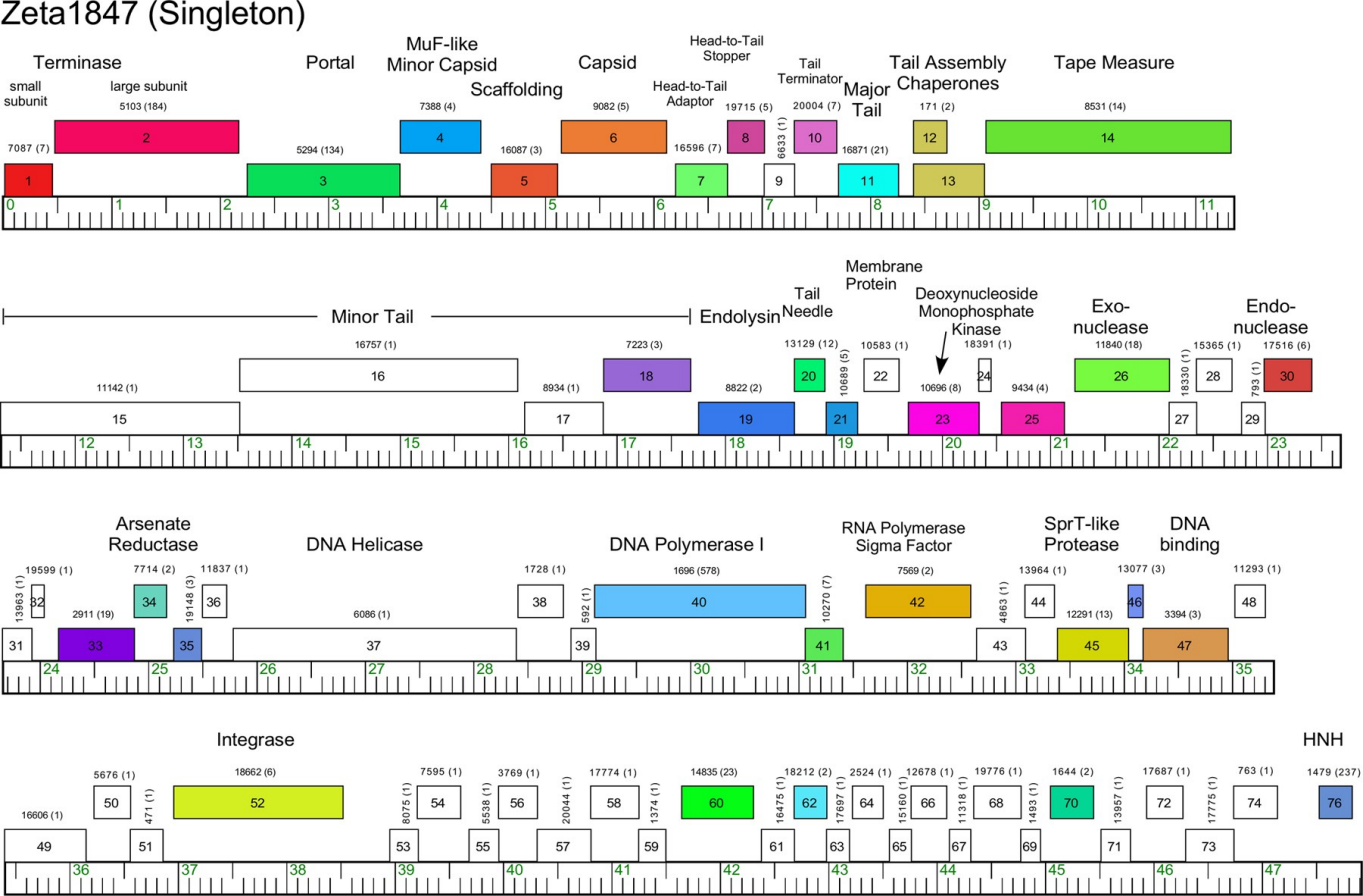
Genome organization of *Microbacterium* Singleton phage Zeta1847. See Fig 6A for details.

Phages Appa, Hendrix, Triscuit, ValentiniPuff, and Zeta1847 are singletons with no close relatives. Appa has a modest-sized genome (38.6 kbp) and all genes are rightwards-transcribed (Fig 18). The virion structure and assembly genes are canonically organized, and the lysis cassette is located downstream of the tail genes. Hendrix has the largest *Microbacterium* phage genome (97.7 kbp) and orphams constitute 75% of the genes (Fig 19). It has a number of unusual genomic features including 40 ORFs located between the terminase and portal protein genes, most of unknown function (Fig 19). It codes for four tRNAs, and also has a RtcB-like RNA ligase gene. Phage Triscuit has a 67.5 kbp genome including a 3,759 bp DTR (Fig 20) with the virion structure and assembly genes transcribed rightwards but displaced about ~15 kbp from the left DTR by 33 ORFs, mostly of unknown function. However, this region includes the lysis cassette which in all other *Microbacterium* phages is located downstream of the tail genes. Although many genes are orphams with no close relatives, several structural genes have homologues in mycobacteriophages, including those in Clusters D, H, R and U (Fig 20). The genes in the right part of the genome are organized into alternately leftwards- and rightwards-transcribed blocks of genes (Fig 20). ValentiniPuff has a 62.5 kbp genome and all of its genes are rightwards-transcribed (Fig 21). Over 82% of its predicted genes are orphams, including an impressive orpham array between the protease and capsid genes (Fig 21). Because of these interruptions the virion structure and assembly gene operon spans nearly 40 kbp. The lysis cassette is located immediately downstream of the tail genes, although there is a second putative endolysin gene (*86*) further downstream (Fig 21), which appears to be a fusion of an N-acetylmuramoyl-L-Alanine amidase domain and an adenosylhomocysteinase domain. Zeta1847 has a 47.9 kbp genome with all of its genes rightwards-transcribed (Fig 22). It has a canonically arranged virion structure and assembly gene operon containing genes related to a variety of other *Microbacterium* phages–particularly Cluster EH, to which it has ~20% shared gene content (S2 Table)–and some mycobacteriophages. Zeta1847 codes for a serine-integrase, which is weakly related to the Int-S of Cluster EH phage Floof (37% aa identity) and is located ~10 kbp from the right genome end. No putative repressor gene was identified.

### Host species specificities of Microbacterium phages

Because the *Microbacterium* phages described here were isolated using a variety of host species and strains, we surveyed the phages to investigate their specificities for different host species. A total of 29 phages representing much of the diversity were tested for infection on the five *Microbacterium* strains used for their isolation as well as *M*. *testaceum*, *M*. *hominis*, and *M*. *terrae* (Table 2). In general, the host strain preferences are specific to the host isolation strain, and the efficiencies of plaquing on strains other that the one used for isolation are typically reduced by many orders of magnitude. The notable exception is Cluster EL phage Count, which infects *M*. *paraoxydans* NRRL B-14843 as efficiently as it infects *M*. *aerolatum* NRRL B-24228 (Table 2). However, there are several instances where efficiency of plaquing is only modestly reduced (1–5 orders of magnitude), which may reflect the ability of the phage to overcome the host range barriers. However, there is no evident pattern indicating that overcoming these barriers is more efficient between any particular pair of strains (Table 2), although we note that only phages isolated on *M*. *foliorum* NRRL B-24224 are able to infect that strain. We know little about the phage preferences for different strains within the species.

### Evolutionary relationships among actinobacteriophages

**Fig 23:**
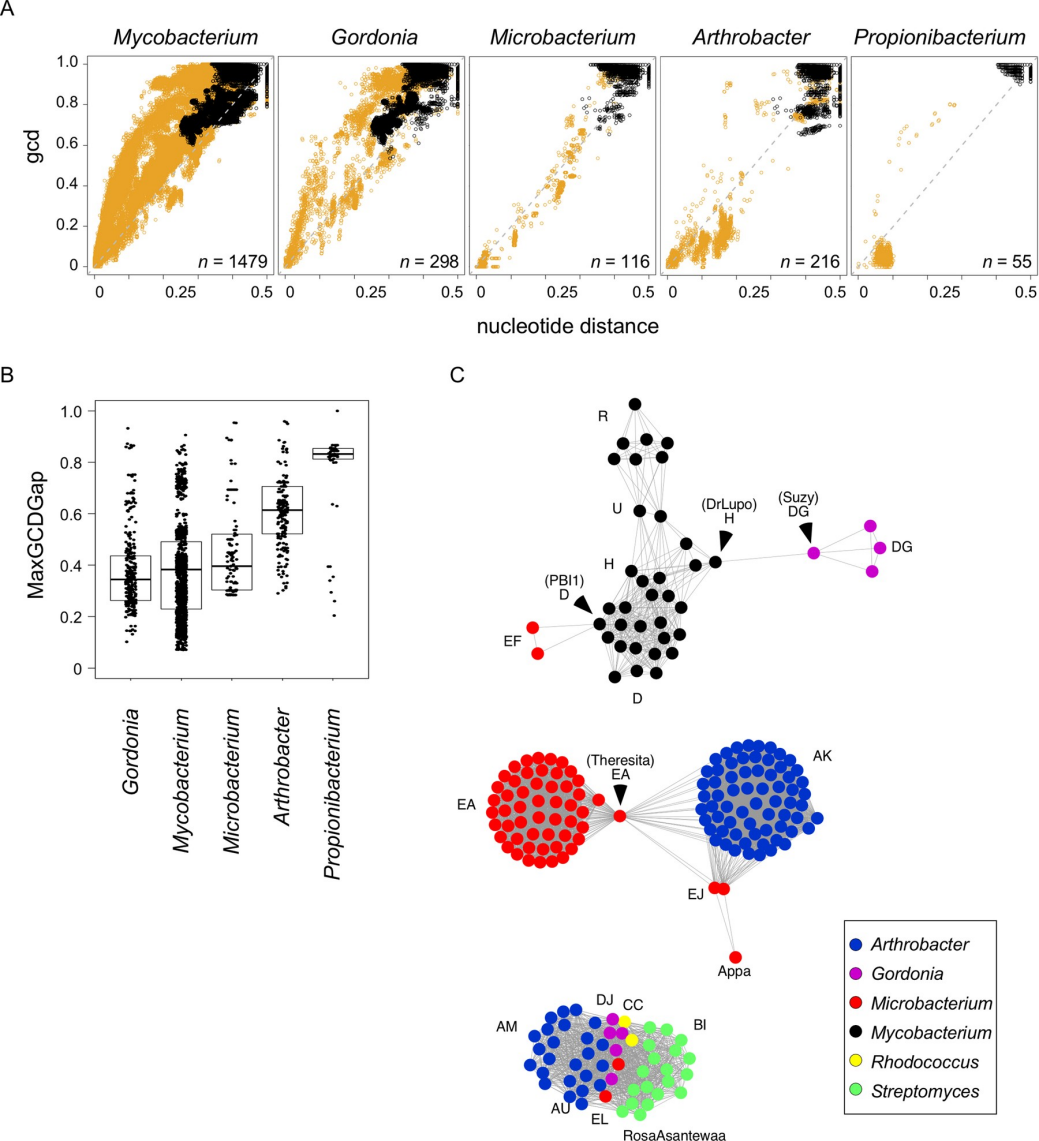
Comparison of phage genomic diversity by host genera. **A.** Genomic similarity plot comparing gene content dissimilarity (gcd) to whole genome nucleotide distance, as previously reported [49]. Each data point reflects a genome comparison involving one phage that infects the indicated host genus and (orange) a second phage that infects the indicated host genus or (black) one phage that infects a different host genus. *n* = number of phages that infect the indicated host genus. **B**. Box plot comparing the MaxGCDGap of phages from different host genera. Each data point is a phage genome, the box depicts the middle 50% of data, and the black bar represents the median. Number of phages per host genus as in panel A. **C**. Three representative genome networks highlight genomic relationships of *Microbacterium* phages to phages of other host genera. Each node represents a phage genome and is colored according to host genus. Edges between nodes represent pairs of phages that exhibit ‘intra-cluster’ genomic similarities (as measured by gene content dissimilarity and nucleotide distance from panel A). Selected phage names or cluster designations are highlighted for reference.

Phages exhibit two evolutionary modes, reflecting different rates of horizontal gene transfer [49]. Evolutionary modes can be impacted by several factors, including phage lifestyle and host preference, which can be evaluated with genomic similarity plots that compare changes in gene content [gene content flux (GCF)] relative to changes in nucleotide sequence (Fig 23A). The evolutionary patterns of *Microbacterium* phages are distinct from phages of several other actinobacterial host genera and exhibit only low GCF, consistent with their obligate lytic lifestyle. The spectrum of genetic diversity within phages of different hosts can be compared using MaxGCDGap [2]. For each phage, the largest gap between two pairwise comparisons in the genomic similarity plot (Fig 23A) is a measure of the phage’s genetic isolation from other sequenced phages in this data set. Unlike phages of *Arthrobacter* and *Propionibacterium*, which exhibit large average MaxGCDGaps, *Microbacterium* phages have an average MaxGCDGap comparable to phages of *Gordonia* and *Mycobacterium* hosts (Fig 23B), reflecting a continuum of diversity rather than well-delineated and clearly separable groups.

The genetic relationships of *Microbacterium* phages to other actinobacteriophages can also be evaluated using genome networks, similar to previous studies [50]. The networks highlight heterogeneous genetic relationships spanning multiple clusters and host genera. *Microbacterium* Cluster EL phages (Camille and Count) form a network with phages from four other host genera and five clusters (Fig 23C). They share 19–32% of their genes with *Streptomyces* Cluster BI phages, the *Streptomyces* singleton RosaAsantewaa, *Arthrobacter* Clusters AM and AU, *Rhodococcus* phages CC, and *Gordonia* Cluster DJ phages (Figs 23C and S17). Even though the proportion of shared genes with these phages is relatively small (40%), they are more closely related than the Cluster EL phages are to any other *Microbacterium* phages, and phages of this type may have relatively mutable host preferences. Consistent with this, we note that Count represents a rare example of a phage that efficiently infects two different *Microbacterium* species (Table 2). The *Microbacterium* phages most closely related to the *Mycobacterium* phages are in Cluster EF. These phages form a genomic network with *Mycobacterium* phages in Cluster D, H, R, and U, as well as *Gordonia* cluster DG phages (Fig 23C). In contrast, *Microbacterium* phages in Clusters EA and EJ, as well as Singleton Appa, form a network with *Arthrobacter* phages in Cluster AK (Figs 23C and S18).

### Concluding remarks

We have described here a large set of newly isolated phages that infect *Microbacterium* bacterial hosts. These span considerable diversity, at a scale similar to phages of *Mycobacterium* and *Gordonia*. However, whereas temperate lifestyles are common among *Mycobacterium* and *Gordonia*, the *Microbacterium* phages are mostly obligatorily lytic, which is reflected both in their genomes’ contents and in the gene content flux analysis (Fig 23). The only *Microbacterium* phages containing integrase genes are the two Cluster EH phages and the singleton Zeta1847, although it is unclear if these make stable lysogens in *Microbacterium* spp. Why phages of host such as *Microbacterium* are predominantly lytic whereas temperate phages are common for *Mycobacterium* and *Gordonia* is unclear, but perhaps is related to the abundance and ecology of the bacteria [51]. The availability of a large diverse collection of sequenced *Microbacterium* phages could potentially be useful for therapeutic applications, as *Microbacterium* infections have been reported in cystic fibrosis patients [20, 21] and other human infections [17, 52].

The *Microbacterium* phage genomes are replete with interesting and novel variations in their genomes and gene organizations. For example, the variations in virion structural genes are striking, including several fusions of functionalities normally encoded in separate genes (e.g. capsid maturation protease and the capsid subunit), and fusion of the VIP2 toxin to the MuF-like protein in the Cluster EH and EJ genomes. The extremely large 13 kbp gene in the Cluster EK phages and the singleton Burro codes for a huge predicted >4,400 amino acid protein, which is likely processed, and virion associated. These are among the largest viral genes described to date.

These *Microbacterium* phages should be a rich source of tools for dissecting and manipulating *Microbacterium* strains. In addition to involvement in human infections, *Microbacterium* strains have been implicated in nitrogen fixation [53] and have potential biotechnological applications [54]. *Microbacterium* phages could be exploited to develop integration-proficient plasmid vectors using the integrase systems in the Cluster EH and Singleton Zeta1847 phages, for recombineering systems using the Exo/Recombinase systems such as those in the Cluster EC, EI, and singleton Appa phages, and for phage-delivery of transposons, allelic exchange substrates, and reporter genes; this mirrors the utilities derived from mycobacteriophages for mycobacterial genetics [1].

Finally, the diversity of the *Microbacterium* phage population appears to be considerable, with the 116 sequenced phages forming 12 clusters and seven singletons. This is a similar diversity profile to those observed when equivalent numbers of *Arthrobacter*, *Mycobacterium*, and *Gordonia* phages were reported [2, 3, 55]. For all three groups the genomic diversity expanded greatly as additional phages were sequenced, and we anticipate similar expansion with an even greater number of sequenced *Microbacterium* phages.

## Materials and methods

### Bacterial strains

The following *Microbacterium* strains for phage isolation were obtained from the American Research Service Culture Collection—Northern Regional Research Laboratory (NRRL) repository: *M*. *aerolatum* NRRL B-24228, *M*. *foliorum* NRRL B-24224, and *M*. *paraoxydans* NRRL B-14843 (deposited as *Kocuria kristinae*; 16S rRNA sequence accession MH368497), *M*. *testaceum* NRRL B-24232, *M*. *hominis* NRRL B-24220, and *M*. *terrae* NRRL B-24214. *M*. *paraoxydans* NWU1 is an environmental isolate from Nebraska Wesleyan University. *M*. *natoriense* ATCC BAA-1032 was obtained from the American Type Culture Collection.

### *Microbacterium* phage isolation, propagation, and virion analysis

Phages were isolated from soil, using either enrichment culture or direct plating as described previously [2, 3, 32]. PYCa media (containing per 1 liter volume: 1.0 g yeast extract, 15 g peptone, 2.5 mL 40% dextrose, and 4.5 ml 1M CaCl_2_) was used for phage isolation and amplification, and cultures were maintained at 25–30°C. For electron microscopy, phage particles were spotted onto formvar and carbon-coated 400 mesh copper grids, rinsed with distilled water and stained with 1% uranyl acetate.

### Genome sequencing and annotation

Sequencing libraries were prepared from double-stranded phage genomic DNA using NEB Ultra II FS Kits and were run on an Illumina MiSeq using 150-cycle v3 Reagent Cartridges yielding 150-base single-end reads representing between 40- and 9,800-fold coverage of each genome. Reads were assembled using Newbler (version 2.9) and quality-controlled using Consed (version 29). Assemblies were checked for completeness, accuracy, orientation, and genomic termini as previously described [56]. Phage genomes were annotated as described previously [33] using DNA Master (http://cobamide2.bio.pitt.edu), GLIMMER [57], GeneMark [58], BLAST [59], Aragorn [60], tRNA-Scan [61], HHPred [62], TMHMM (http://www.cbs.dtu.dk/services/TMHMM/), SOSUI [63], DotPlot [64], Splitstree [35], kAlign [65], and MEME [66] and Phamerator [34] using database Actinobacteriophage_2422.

### Database construction

The Phamerator database ‘Actinobacteriophage_2422’ contains 2,422 phages that infect hosts in the phylum Actinobacteria, derived from the SEA-PHAGES program [67] and from GenBank, and includes Min1 [29]. This MySQL database is publicly available (http://phamerator.webfactional.com/databases_Hatfull) [5].

### Genomic similarity plots and MaxGCDGap

Comparisons of gene content dissimilarity to nucleotide distance between phages based on their host genus were performed as previously described [49]. Identification and analysis of MaxGCDGap values between phages based on their host genus was performed as previously described [2].

### Genome network construction

For network comparisons, all phage pairwise comparisons with ‘intra-cluster’ distances (gene content dissimilarity < 0.89 and nucleotide distance < 0.42) were retained and all other data was discarded [49]. Using intra-cluster pairwise comparisons, phage networks were constructed using Cytoscape (version 3.4.0) [68]. Each node represents a phage genome, and if two nodes exhibit intra-cluster genomic similarity, they are connected by an edge. The length of each edge has no biological meaning. A network therefore represents a group of phages (irrespective of their formal Cluster designation), in which each phage exhibits intra-cluster genomic similarity to at least one other phage within the group and to no phages outside of the group. Within each network, nodes were automatically arranged for clarity using the Prefuse Force Directed Layout algorithm.

### Microbacterium phylogenetics

Actinobacterium phylogenies were constructed using a Neighbor-Joining method with MEGA7 [36–38]. The optimal tree with the sum of branch length = 0.59180754 is shown in Fig 4. The evolutionary distances were computed using the Maximum Composite Likelihood method in the units of the number of base substitutions per site. Codon positions included were 1st+2nd+3rd+Noncoding, and all positions containing gaps and missing data were eliminated. There were a total of 1216 positions in the final dataset.

### Host range analysis

Lysates of phages were serially diluted in phage buffer and 2.5 μl of ten-fold dilutions were spotted on fresh lawns of *M*. *aerolatum* NRRL B-24228, *M*. *foliorum* NRRL B-24224, *M*. *natoriense* ATCC BAA-1032, *M*. *paraoxydans* NRRL B-14843, and *M*. *paraoxydans* NWU1, *M*. *testaceum*, *M*. *terrae*, and *M*. *hominis*. Plates were incubated at 30°C and plaque formation was scored after 2 days of growth.

## Supporting information

S1 Table Average nucleotide identities of *Microbacterium* phage genomes. Pairwise average nucleotide identities were calculated for 115 *Microbacterium* phages using DNA Master. Phage Min1 is not included.
(XLSX)

S2 Table Gene content similarity of *Microbacterium* phage genomes. Pairwise gene content similarity was calculated by identifying the number of phams that are present in both phages, dividing that number by the number of total phams present in each phage, then averaging the two values.
(XLSX)

S1 Fig Genome organization of *Microbacterium* subcluster EA2 phage Eleri. The genome of *Microbacterium* Subcluster EA2 phage Eleri is shown with predicted genes shown as boxes either above or below the genome indicating rightward- and rightward-transcription, respectively. Gene numbers are shown within each gene box. Phamily designations are shown above or below each gene with the numbers of phamily members in parentheses; genes are colored according to the phamily designations. White boxes represent ‘orphams’, genes with no close relatives in this dataset. Phamily assignments were determined using Phamerator [34] and database Actinobacteriophage_2422. Predicted gene functions are indicated.
(TIF)

S2 Fig Genome organization of *Microbacterium* subcluster EA3 phage casey. See S1 Fig for details.
(TIF)

S3 Fig Genome organization of *Microbacterium* Subcluster EA4 golden. See S1 Fig for details.
(TIF)

S4 Fig Genome organization of *Microbacterium* subcluster EA5 neferthena. See S1 Fig for details.
(TIF)

S5 Fig Genome organization of *Microbacterium* subcluster EA6 chepli. See S1 Fig for details.
(TIF)

S6 Fig Genome organization of *Microbacterium* Subcluster EA7 theresita. See S1 Fig for details.
(TIF)

S7 Fig Genome organization of *Microbacterium* subcluster EA8 schubert. See S1 Fig for details.
(TIF)

S8 Fig Genome organizations of *Microbacterium* cluster EB phages. The eleven Cluster EB genomes are shown with genes represented as boxes above or below each genome reflecting leftwards- and rightwards-transcription, respectively; genes are colored according to their phamily assignments. Pairwise nucleotide sequence similarity is displayed by spectrum-coloring between genomes, with violet representing greatest similarity and red the least similar, above a threshold E value of 10^−5^. Maps were generated using Phamerator [34] and database Actinobacteriophage_2422.
(TIF)

S9 Fig Genome organizations of *Microbacterium* Cluster EC phages. See S8 Fig for details.
(TIF)

S10 Fig Repeated sequence motifs in *Microbacterium* phages. **A**. Conserved sequence motifs in Cluster EC phage Quhwah. Each of 12 occurrences of the repeat motifs in the Quhwah genomes are aligned with their coordinates shown to the right. The consensus sequence is shown below with totally conserved residues shown in upper case type and residues present in 9–11 of the repeats are shown lower case type. Each motif is positioned 22–30 bp upstream of the translation start codon of the downstream gene. **B.** Conserved Start Associated Sequence (SAS) motifs in Cluster EF phage AnnaSerena. Ten repeated motifs in phage AnnaSerena are located immediately upstream of translational start codons (underlined) in the position typically located by the ribosome biding site (RBS), although they are much more highly conserved than RBS’s typically are. The consensus sequence is shown below for both the AnnaSerena sites, as well as the consensus for similar SAS sites reported in Cluster K mycobacteriophages [44]. The extreme 3’ end of the 16S rRNA gene is shown, aligned to show complementarity with the AnnaSerena SAS consensus. **C**. Conserved Extended Start Associated Sequence (ESAS) motifs in Cluster EF phage AnnaSerena. Each of the SAS motifs shown in panel B is accompanied by an Extended Start Associated Seuqence (ESAS) positioned immediately upstream of the SAS; two additional ESAS are present upstream of genes *39* and *42* (see Fig 11) which appear to lack SAS motifs. The ESAS sequence is poorly conserved, but is centered around a 5’-GTAGAG sequence that is very well conserved, flanked by more weakly conserved positions. Consensus sequences present in 11–12 of the 12 conserved sequences are shown in upper case type, and those in 7–10 are shown in lower case type. **D**. Conserved repeated sequences in the genome of cluster EG phage Hyperion. The Hyperion genome contains ten repeats of a sequence motif containing two short inverted motifs (yellow) separated by two base pairs. The sequence shown is the bottom strand, and the motifs are all upstream of leftwards-transcribed genes (see Fig 12), positioned 14–21 bp upstream of the translational start site of the downstream gene (with the exception of site #9). The same repeat is found in similar positions in the other two cluster EG phages, OneinaGillian and Squash (not shown), which is notable since OneinaGillan shares little nucleotide or protein sequence similarity with Hyperion in this region. **E**. Repeated sequences in the MementoMori genome. The Cluster EI phages each contain five copies of a repeated sequence motif located in short intergenic regions (see Fig 14). The consensus sequence is shown with bases conversed in all five motifs indicated in upper case type, and those conserved in at least three motifs is shown as lower case type.
(TIF)

S11 Fig Genome organizations of *Microbacterium* cluster ED phages. See S8 Fig for details.
(TIF)

S12 Fig Genome organizations of *Microbacterium* cluster EE phages. See S8 Fig for details.
(TIF)

S13 Fig Genome organizations of *Microbacterium* cluster EG phages. **A.** Alignment of Cluster EG phages Hyperion, Squash, and OneinaGillian. See S8 Fig for details. **B**. Expanded view of the right ends of the three Cluster EG phages showing the positions (vertical arrows) of conserved short inverted repeat motifs; see Figs 12 and S10 for further details. The same sequence (consensus 5’-GATCAACCNNGGTTGATC) is conserved in all three genomes notwithstanding the DNA sequence divergence in these parts of the genomes. In OneinaGillian there is an additional site at 35982..35999 that is not start-associated.
(TIF)

S14 Fig Genome organizations of *Microbacterium* cluster EH phages. See S8 Fig for details.
(TIF)

S15 Fig Genome organizations of *Microbacterium* cluster EI phages. See S8 Fig for details.
(TIF)

S16 Fig Genome organizations of *Microbacterium* cluster EJ phages. See S8 Fig for details.
(TIF)

S17 Fig Relationships between phage genomes of different *Actinobacterium* hosts. Arcadia is a Cluster AM *Arthrobacter* phage, Pepy6 is a *Rhodococcus* Cluster CC phage, Gravy is a Cluster DJ Gordonia phage, RosaAsantewaa is a *Streptomyces* singleton, Bing is a Subcluster BI1 *Streptomyces* phage, and Count and Camille are Cluster EL *Microbacterium* phages. See S8 Fig for details.
(TIF)

S18 Fig Inter-cluster relationships among *Microbacterium* phages. Alignments of genome maps of phages TeddyBear (Subcuster EA1), Theresita (Subcluster EA7), Zorro (Cluster AK), Goodman (Cluster EJ), and Appa (singleton). See S8 Fig for details.
(TIF)

S19 Fig Raw image of Fig 16B. Lanes 5 and 6 as well as the marker lane are shown in Fig 16B.
(TIF)
