# Supplementary figures and images for: Genomic diversity of bacteriophages infecting Microbacterium spp
Source: PLoS One. 2020 Jun 18;15(6):e0234636. doi: 10.1371/journal.pone.0234636 (PMC7302621; doi:10.1371/journal.pone.0234636)

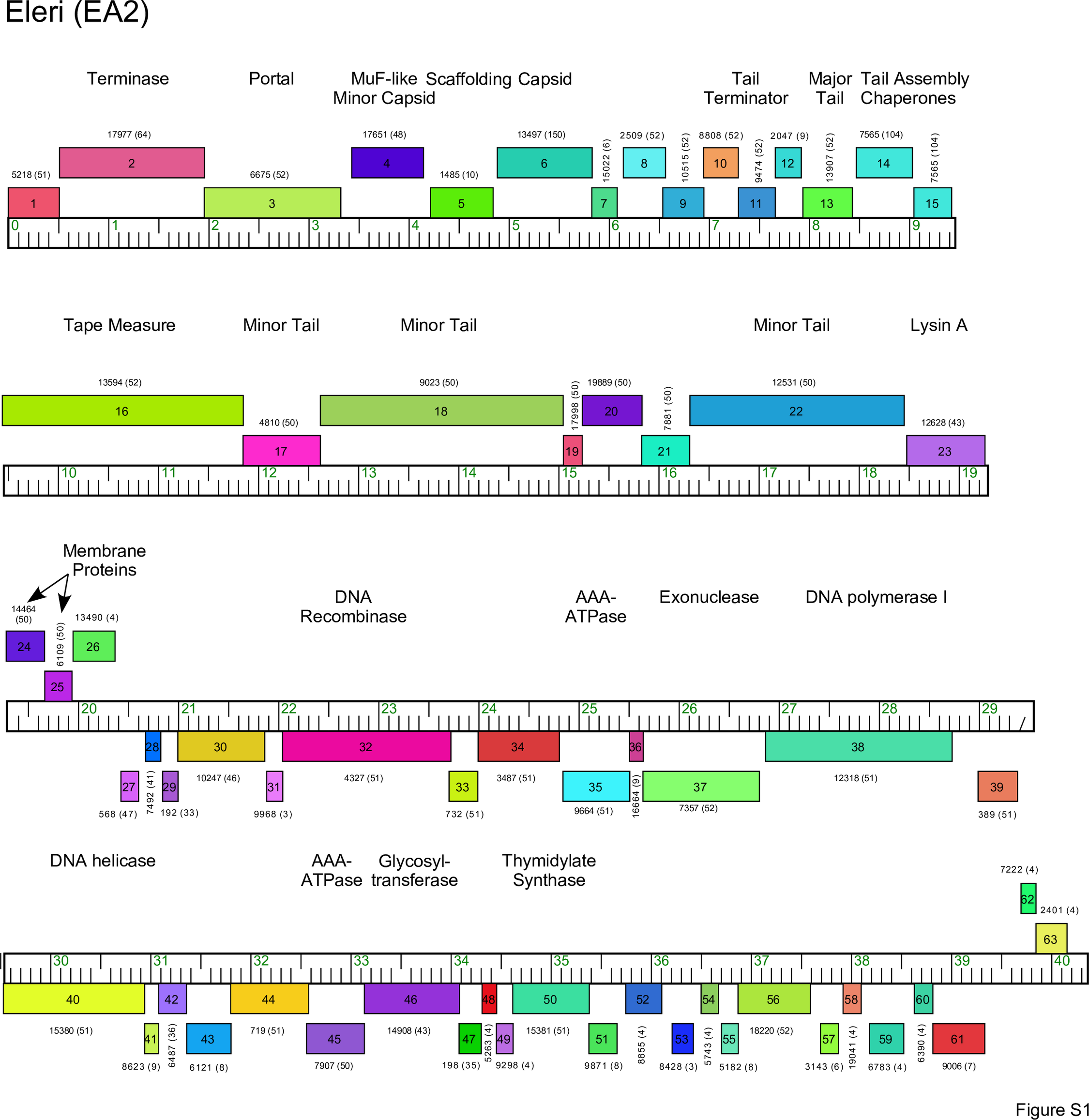

Supplement: S1 Fig — The genome of Microbacterium Subcluster EA2 phage Eleri is shown with predicted genes shown as boxes either above or below the genome indicating rightward- and rightward-transcription, respectively. Gene numbers are shown within each gene box. Phamily designations are shown above or below each gene with the numbers of phamily members in parentheses; genes are colored according to the phamily designations. White boxes represent ‘orphams’, genes with no close relatives in this dataset. Phamily assignments were determined using Phamerator [34] and database Actinobacteriophage_2422. Predicted gene functions are indicated. (TIF) [file pone.0234636.s003.tif]

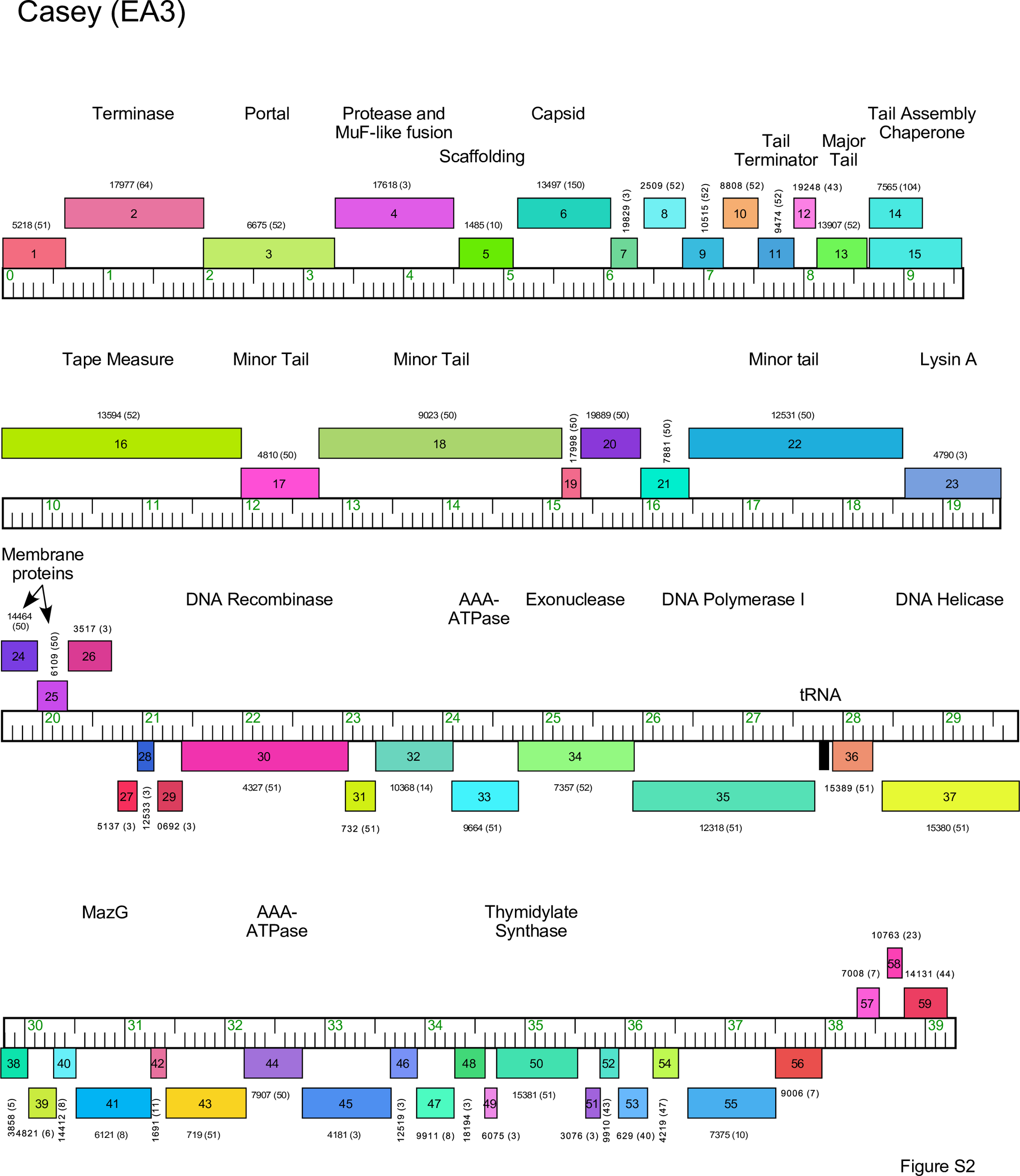

Supplement: S2 Fig — See S1 Fig for details. (TIF) [file pone.0234636.s004.tif]

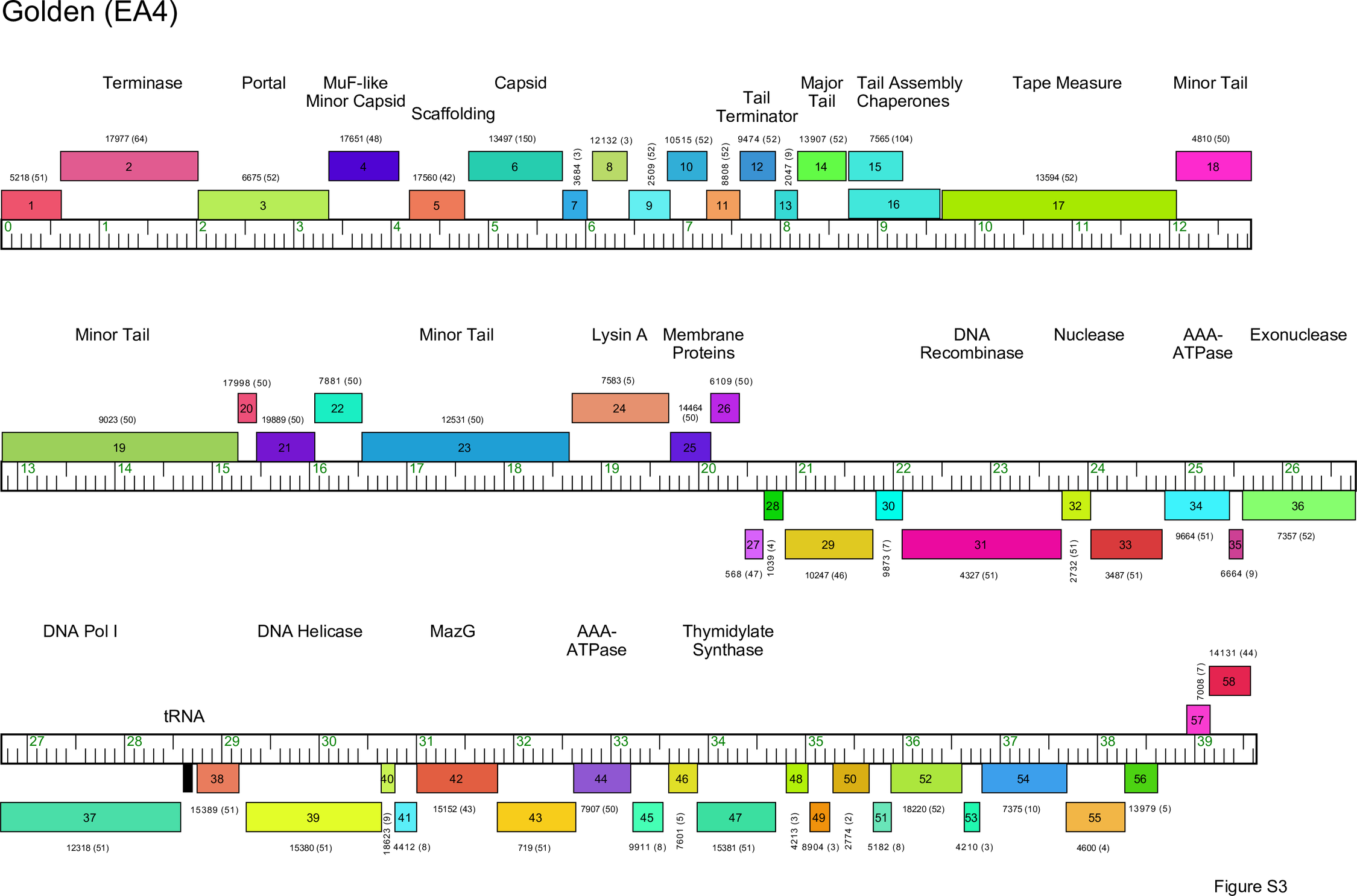

Supplement: S3 Fig — See S1 Fig for details. (TIF) [file pone.0234636.s005.tif]

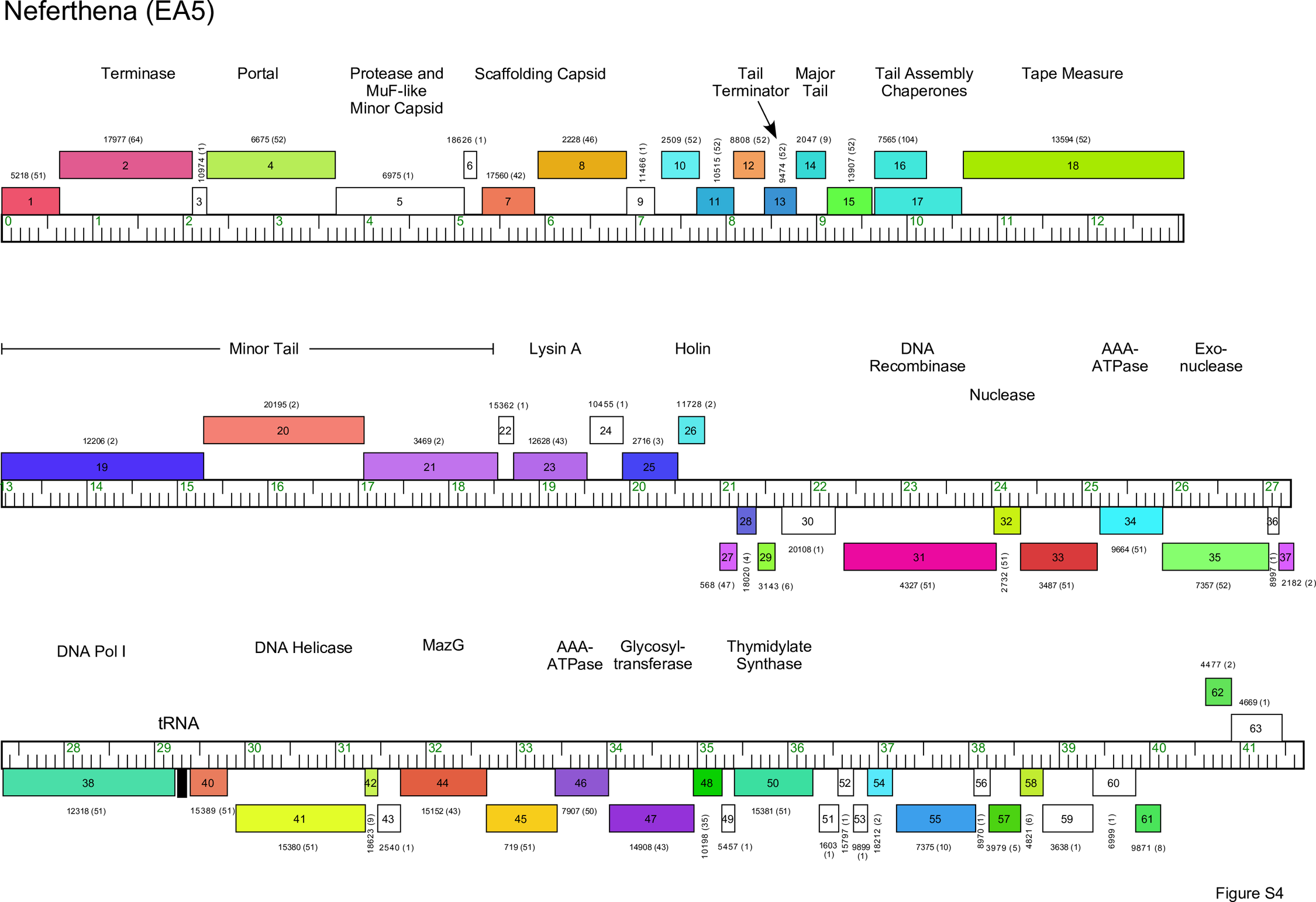

Supplement: S4 Fig — See S1 Fig for details. (TIF) [file pone.0234636.s006.tif]

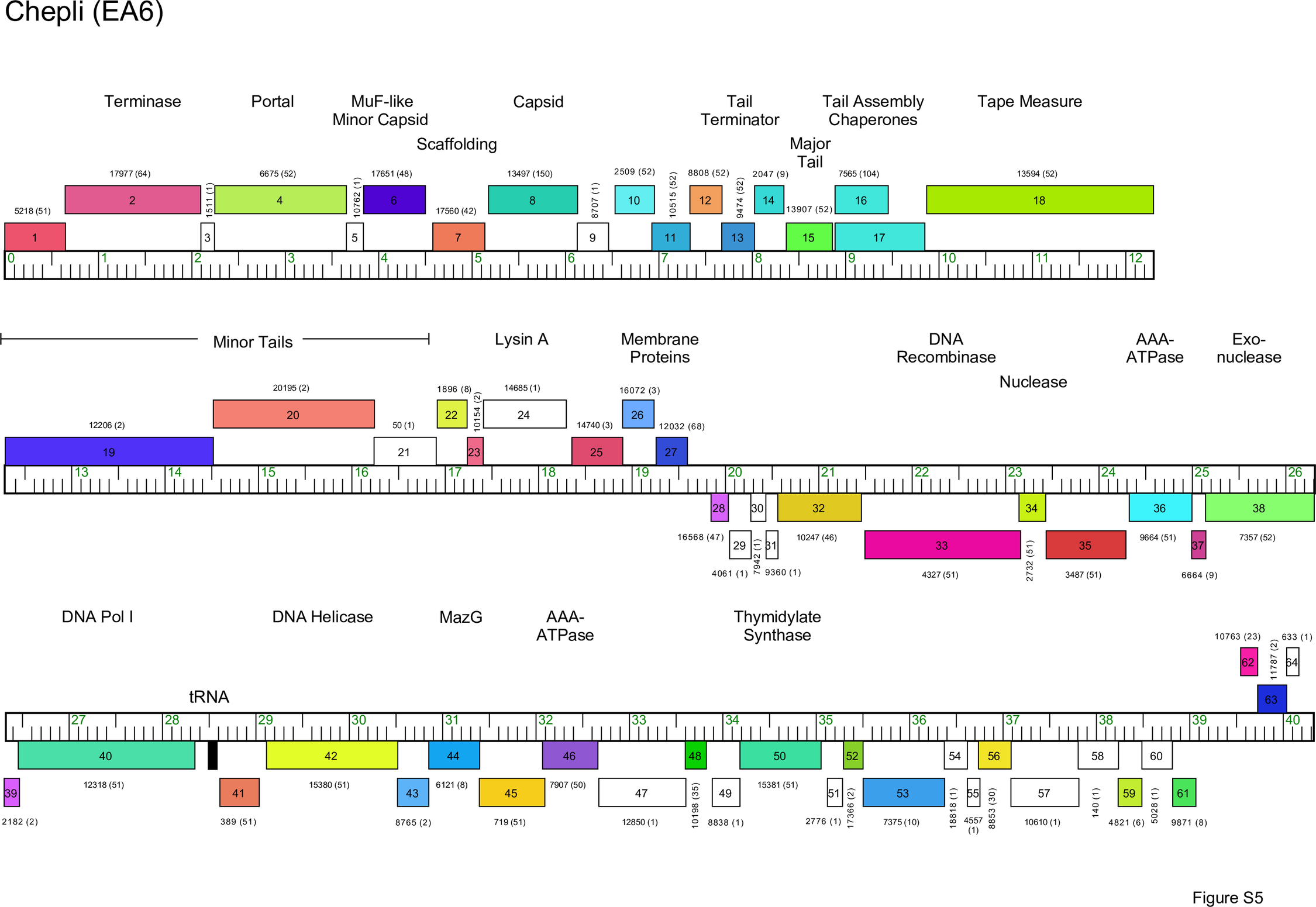

Supplement: S5 Fig — See S1 Fig for details. (TIF) [file pone.0234636.s007.tif]

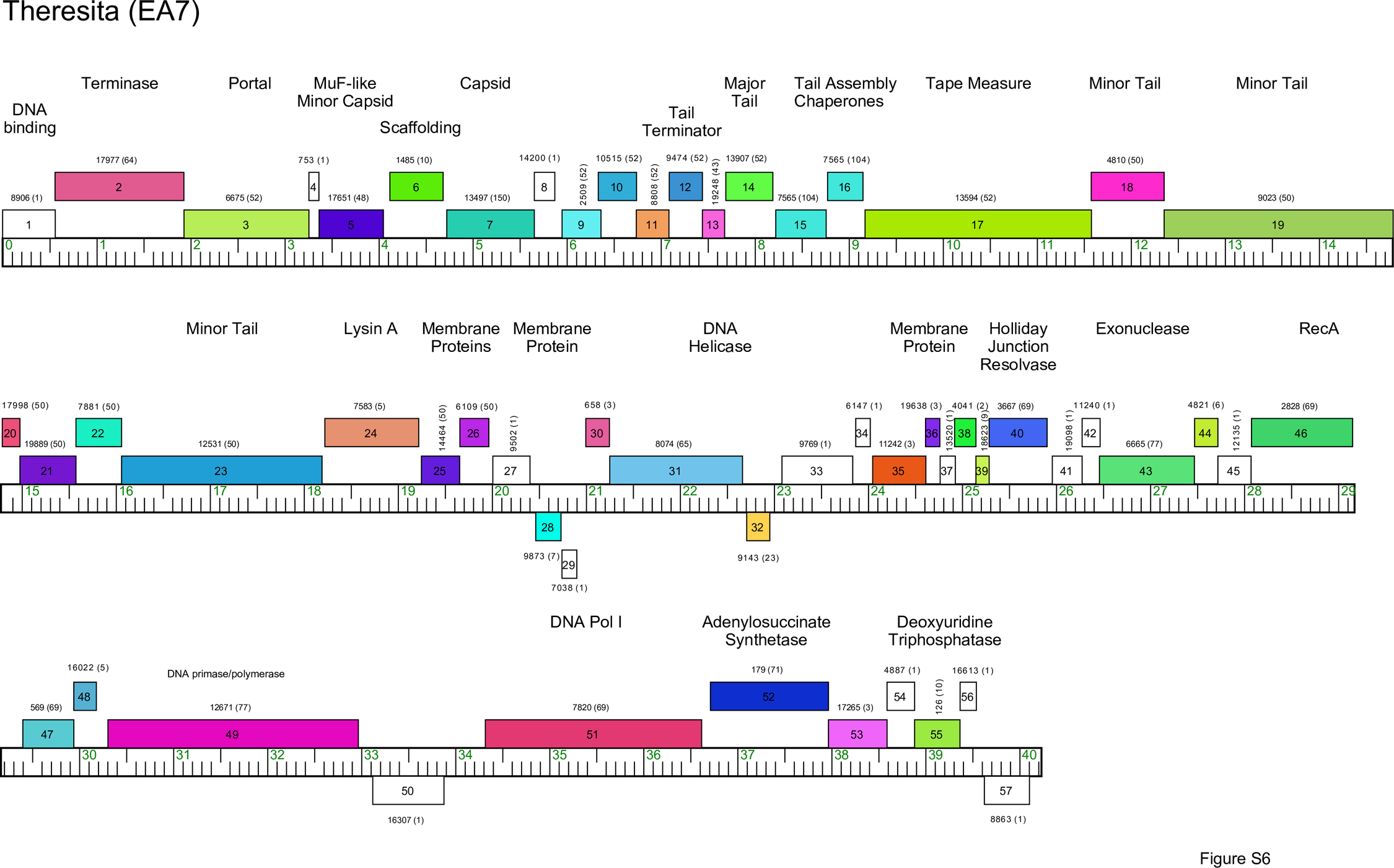

Supplement: S6 Fig — See S1 Fig for details. (TIF) [file pone.0234636.s008.tif]

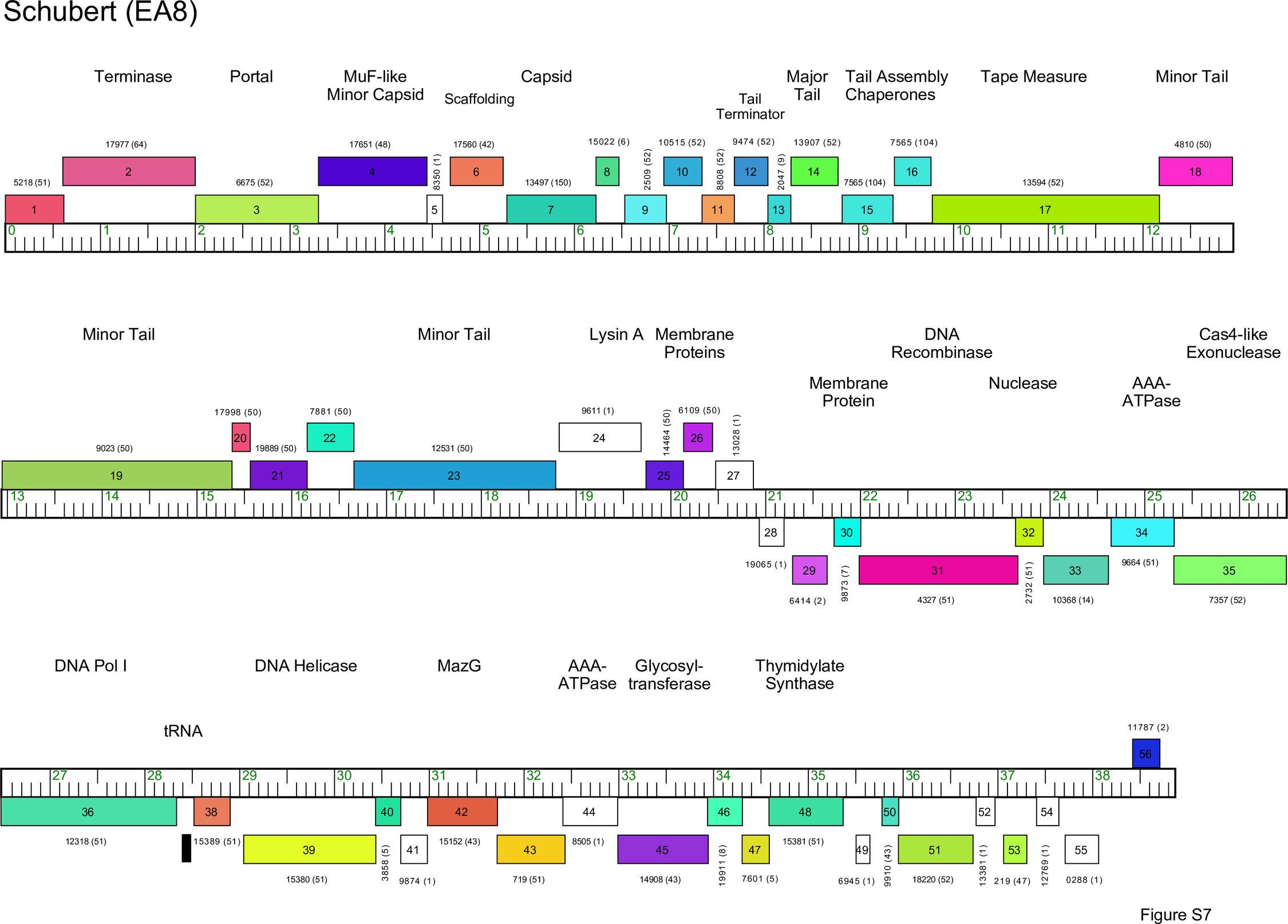

Supplement: S7 Fig — See S1 Fig for details. (TIF) [file pone.0234636.s009.tif]

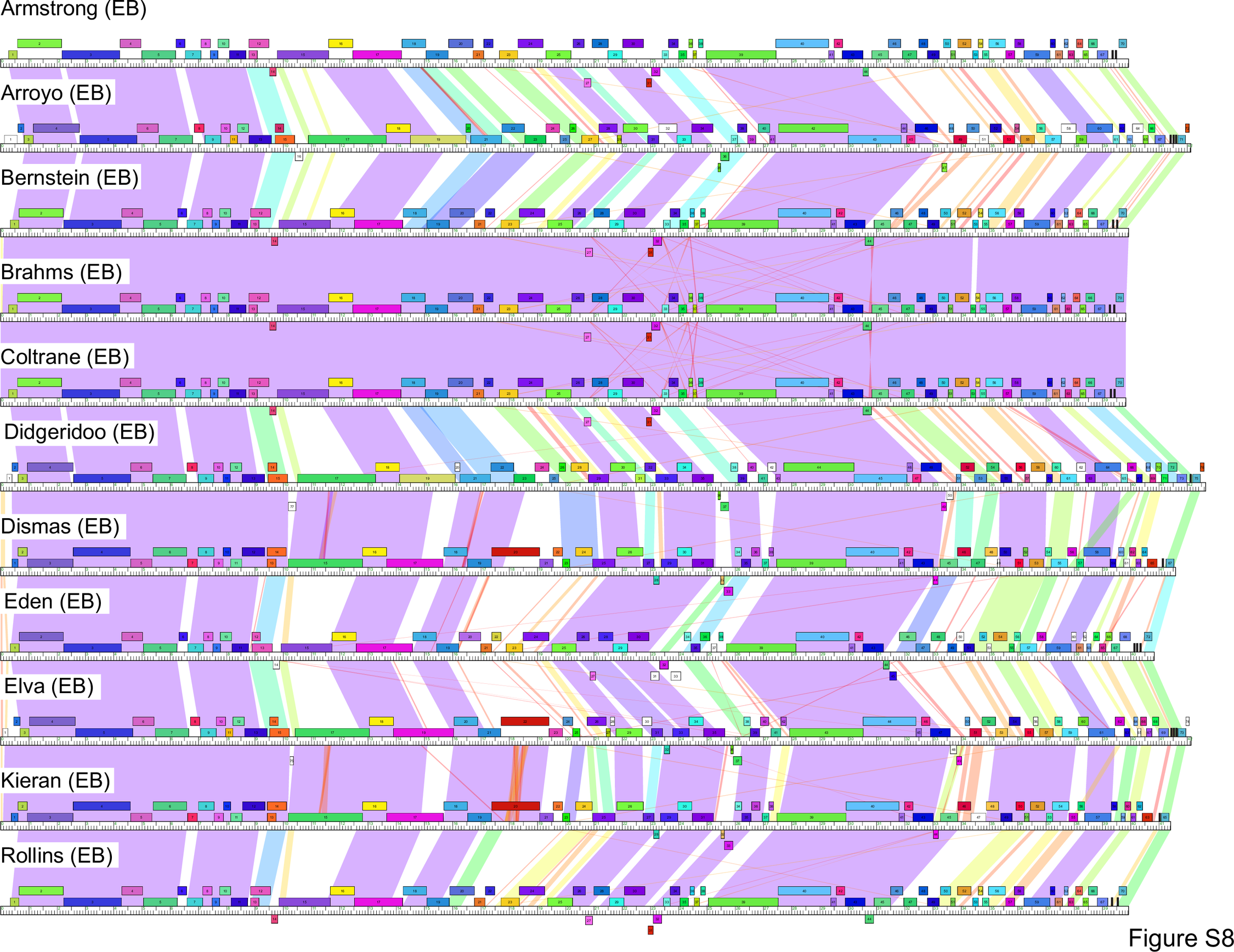

Supplement: S8 Fig — The eleven Cluster EB genomes are shown with genes represented as boxes above or below each genome reflecting leftwards- and rightwards-transcription, respectively; genes are colored according to their phamily assignments. Pairwise nucleotide sequence similarity is displayed by spectrum-coloring between genomes, with violet representing greatest similarity and red the least similar, above a threshold E value of 10−5. Maps were generated using Phamerator [34] and database Actinobacteriophage_2422. (TIF) [file pone.0234636.s010.tif]

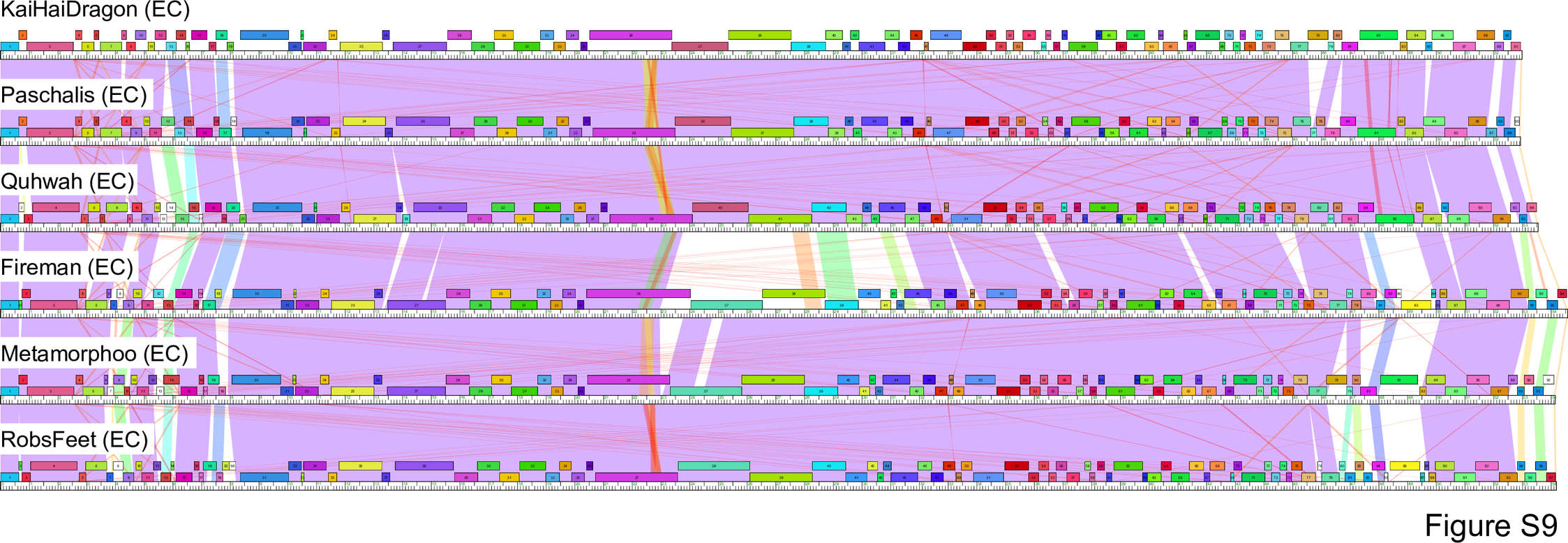

Supplement: S9 Fig — See S8 Fig for details. (TIF) [file pone.0234636.s011.tif]

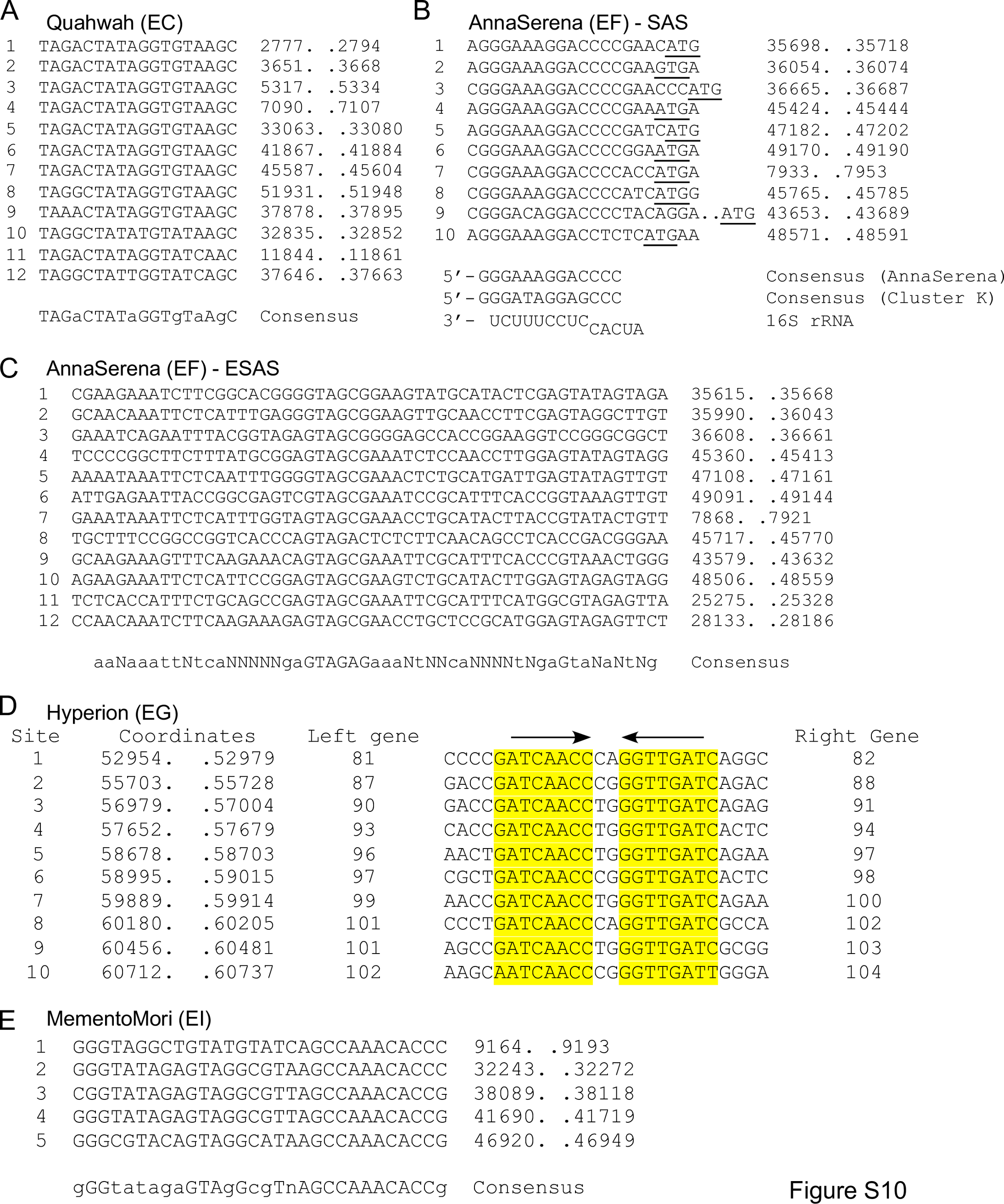

Supplement: S10 Fig — A. Conserved sequence motifs in Cluster EC phage Quhwah. Each of 12 occurrences of the repeat motifs in the Quhwah genomes are aligned with their coordinates shown to the right. The consensus sequence is shown below with totally conserved residues shown in upper case type and residues present in 9–11 of the repeats are shown lower case type. Each motif is positioned 22–30 bp upstream of the translation start codon of the downstream gene. B. Conserved Start Associated Sequence (SAS) motifs in Cluster EF phage AnnaSerena. Ten repeated motifs in phage AnnaSerena are located immediately upstream of translational start codons (underlined) in the position typically located by the ribosome biding site (RBS), although they are much more highly conserved than RBS’s typically are. The consensus sequence is shown below for both the AnnaSerena sites, as well as the consensus for similar SAS sites reported in Cluster K mycobacteriophages [44]. The extreme 3’ end of the 16S rRNA gene is shown, aligned to show complementarity with the AnnaSerena SAS consensus. C. Conserved Extended Start Associated Sequence (ESAS) motifs in Cluster EF phage AnnaSerena. Each of the SAS motifs shown in panel B is accompanied by an Extended Start Associated Seuqence (ESAS) positioned immediately upstream of the SAS; two additional ESAS are present upstream of genes 39 and 42 (see Fig 11) which appear to lack SAS motifs. The ESAS sequence is poorly conserved, but is centered around a 5’-GTAGAG sequence that is very well conserved, flanked by more weakly conserved positions. Consensus sequences present in 11–12 of the 12 conserved sequences are shown in upper case type, and those in 7–10 are shown in lower case type. D. Conserved repeated sequences in the genome of cluster EG phage Hyperion. The Hyperion genome contains ten repeats of a sequence motif containing two short inverted motifs (yellow) separated by two base pairs. The sequence shown is the bottom strand, and the motifs are all upstream of le [file pone.0234636.s012.tif]

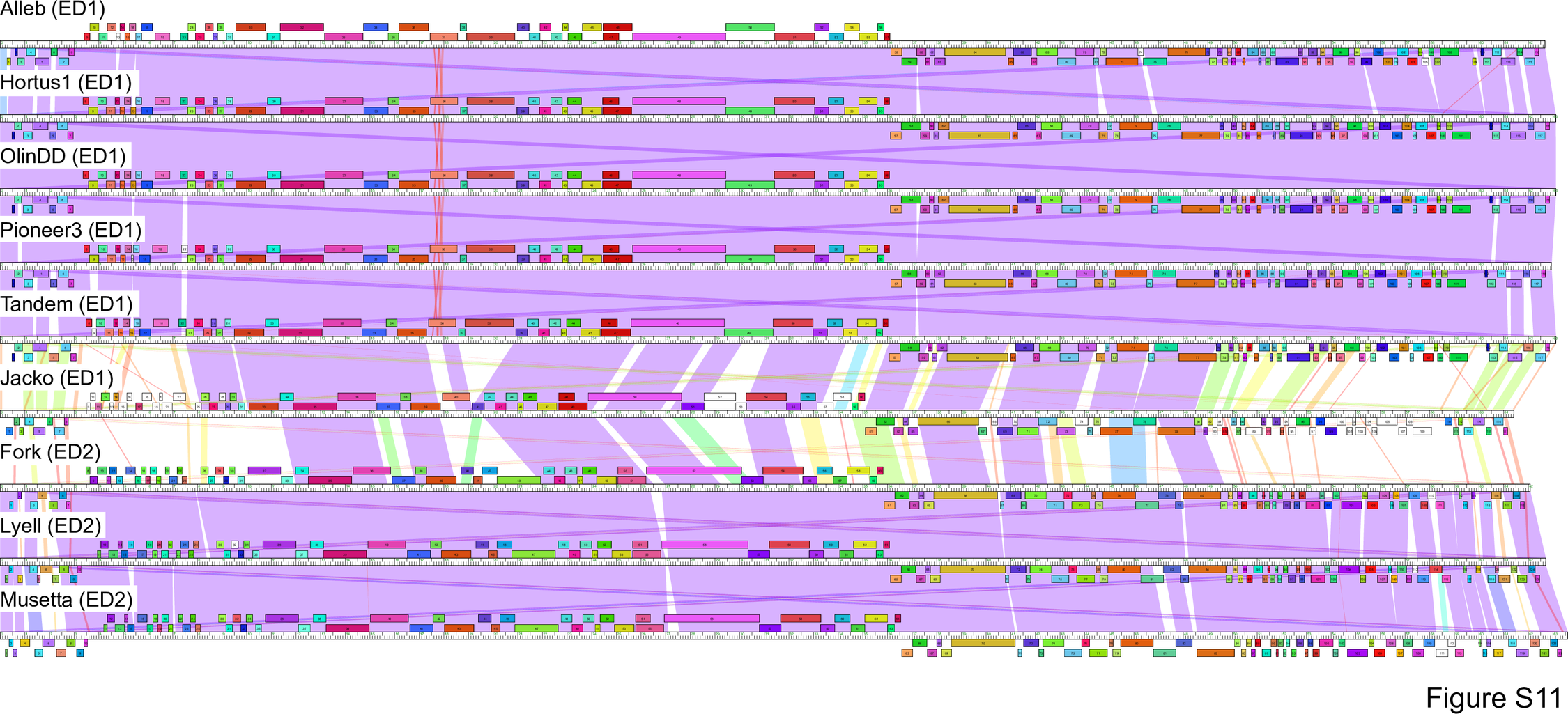

Supplement: S11 Fig — See S8 Fig for details. (TIF) [file pone.0234636.s013.tif]

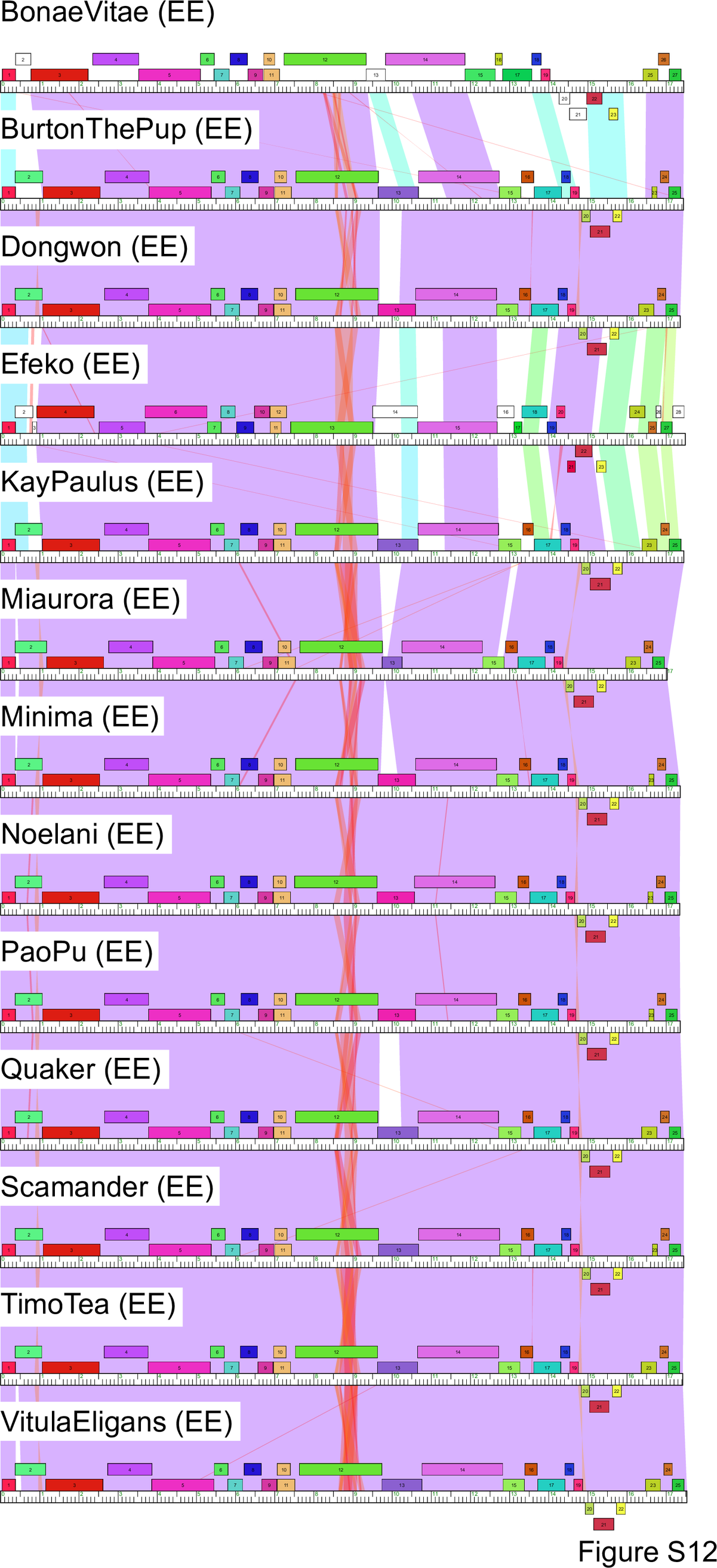

Supplement: S12 Fig — See S8 Fig for details. (TIF) [file pone.0234636.s014.tif]

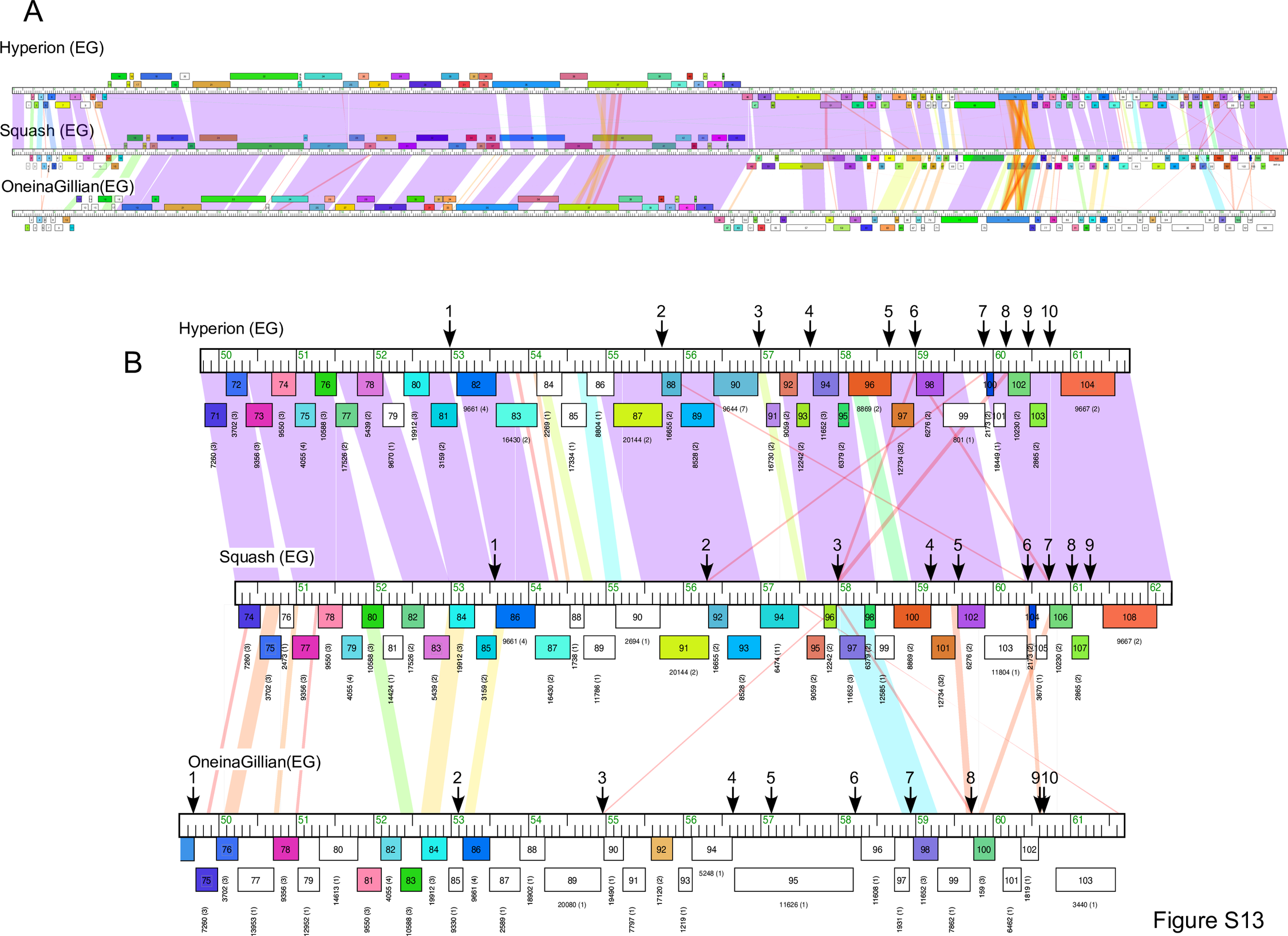

Supplement: S13 Fig — A. Alignment of Cluster EG phages Hyperion, Squash, and OneinaGillian. See S8 Fig for details. B. Expanded view of the right ends of the three Cluster EG phages showing the positions (vertical arrows) of conserved short inverted repeat motifs; see Figs 12 and S10 for further details. The same sequence (consensus 5’-GATCAACCNNGGTTGATC) is conserved in all three genomes notwithstanding the DNA sequence divergence in these parts of the genomes. In OneinaGillian there is an additional site at 35982..35999 that is not start-associated. (TIF) [file pone.0234636.s015.tif]

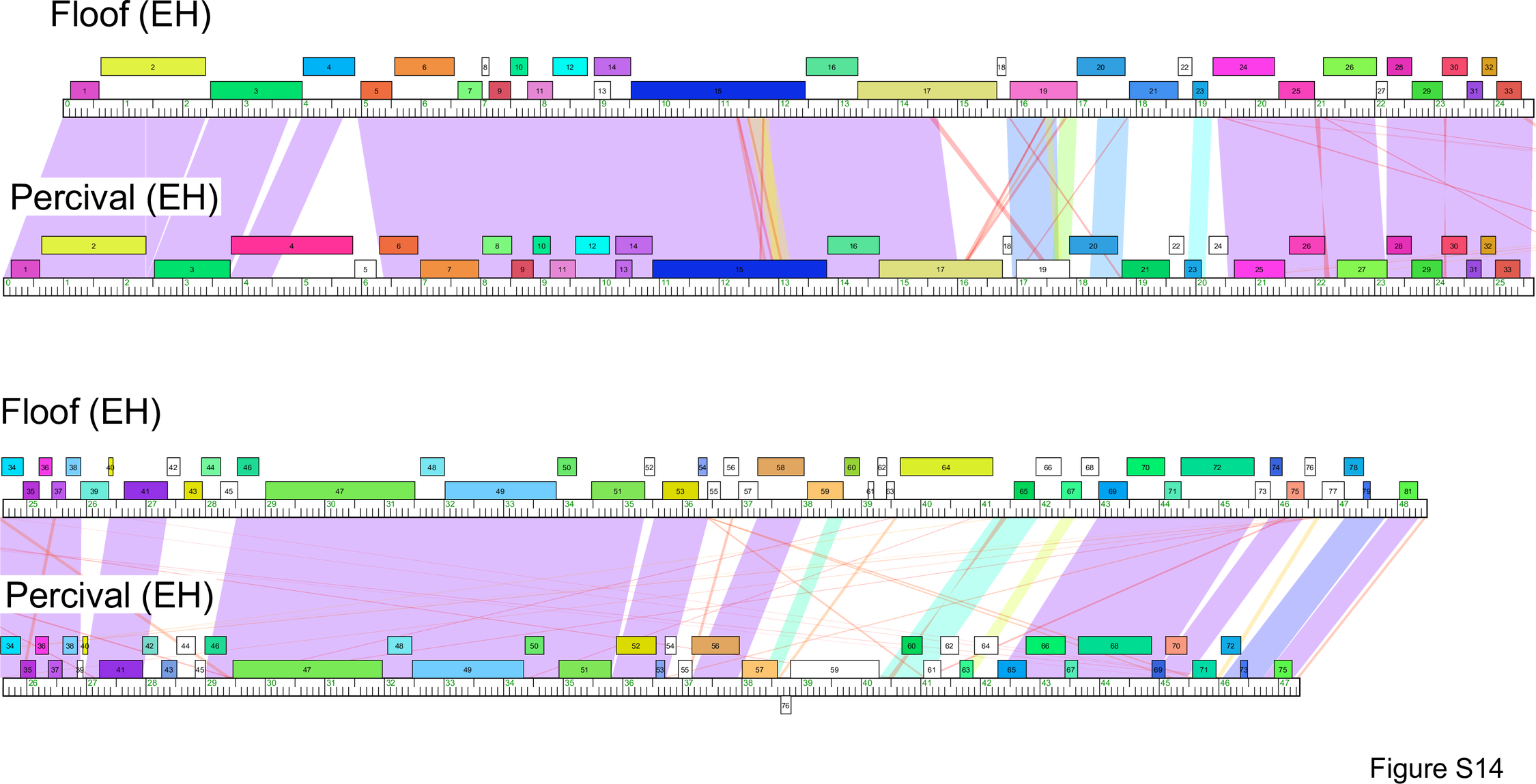

Supplement: S14 Fig — See S8 Fig for details. (TIF) [file pone.0234636.s016.tif]

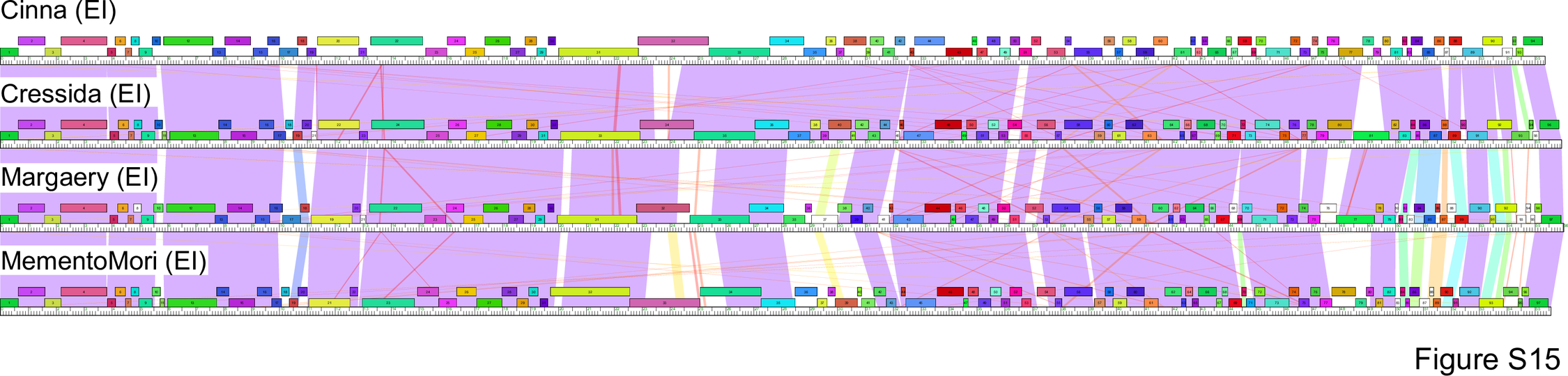

Supplement: S15 Fig — See S8 Fig for details. (TIF) [file pone.0234636.s017.tif]

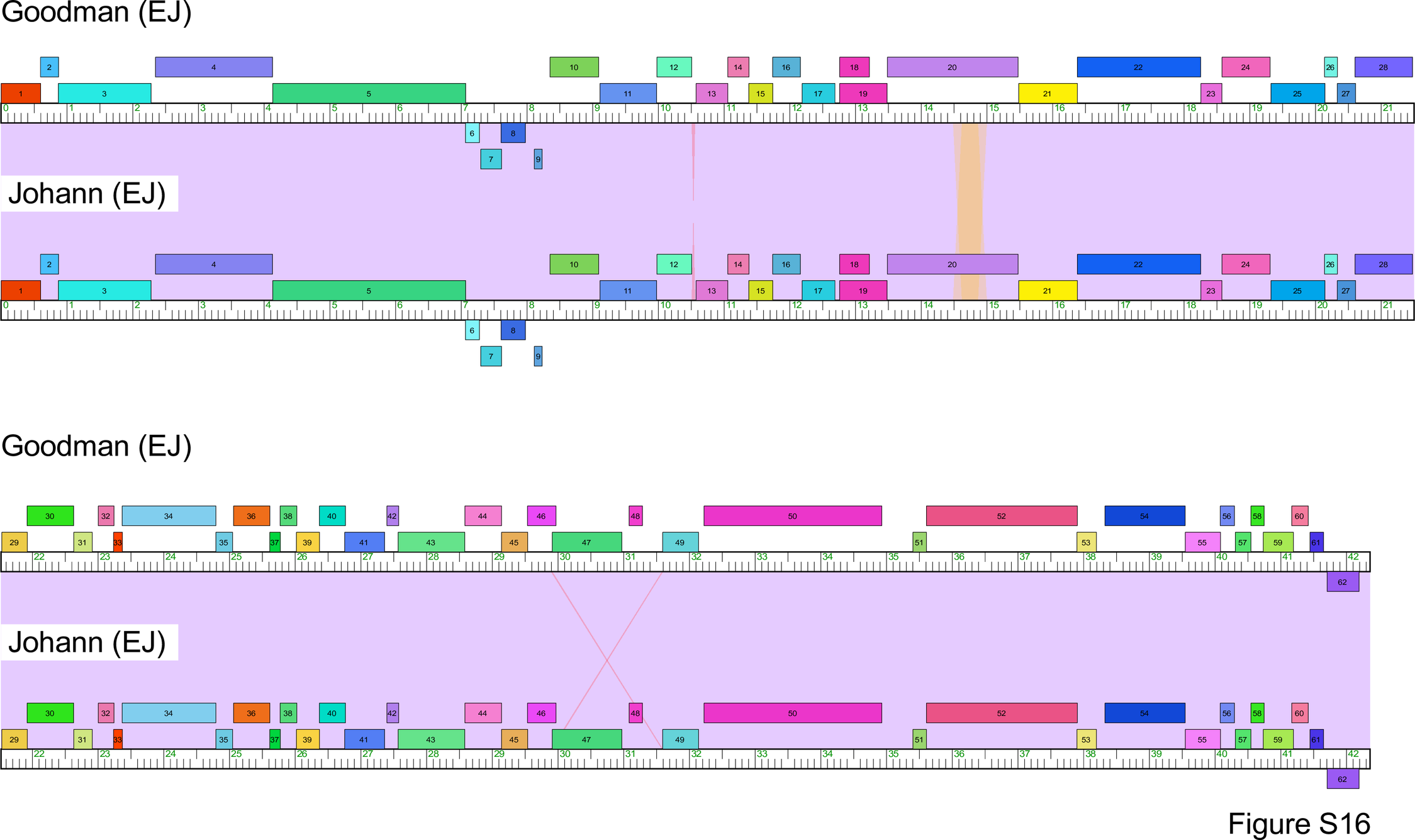

Supplement: S16 Fig — See S8 Fig for details. (TIF) [file pone.0234636.s018.tif]

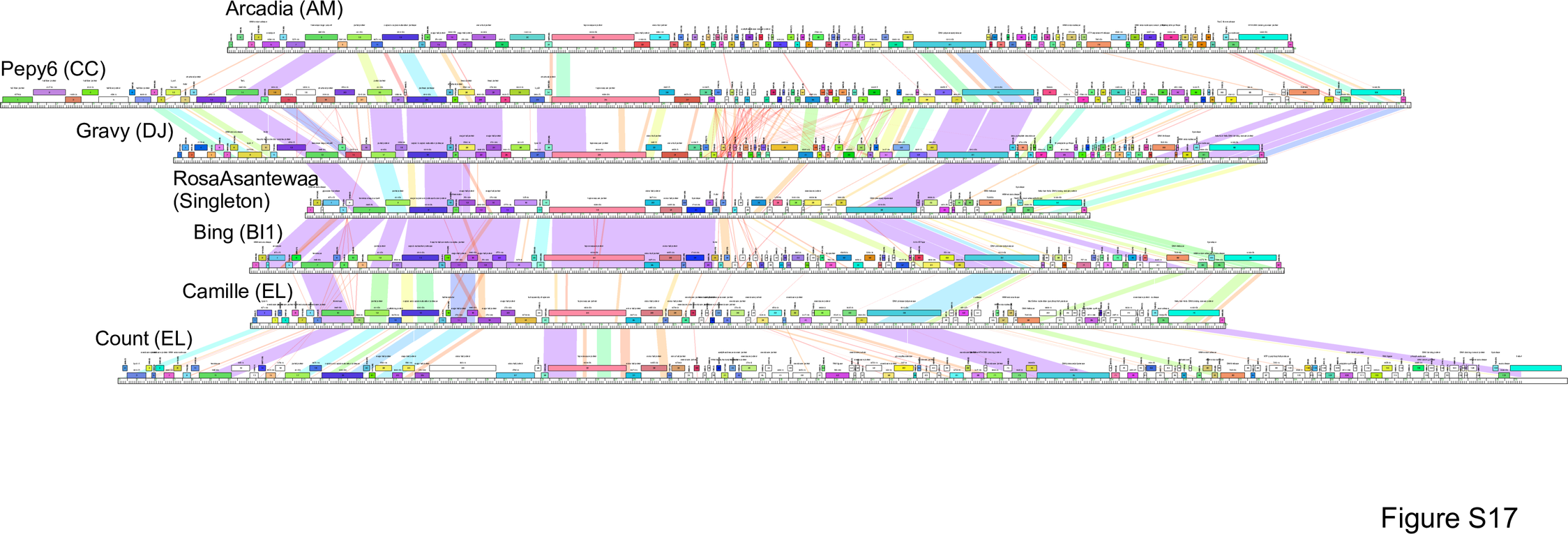

Supplement: S17 Fig — Arcadia is a Cluster AM Arthrobacter phage, Pepy6 is a Rhodococcus Cluster CC phage, Gravy is a Cluster DJ Gordonia phage, RosaAsantewaa is a Streptomyces singleton, Bing is a Subcluster BI1 Streptomyces phage, and Count and Camille are Cluster EL Microbacterium phages. See S8 Fig for details. (TIF) [file pone.0234636.s019.tif]

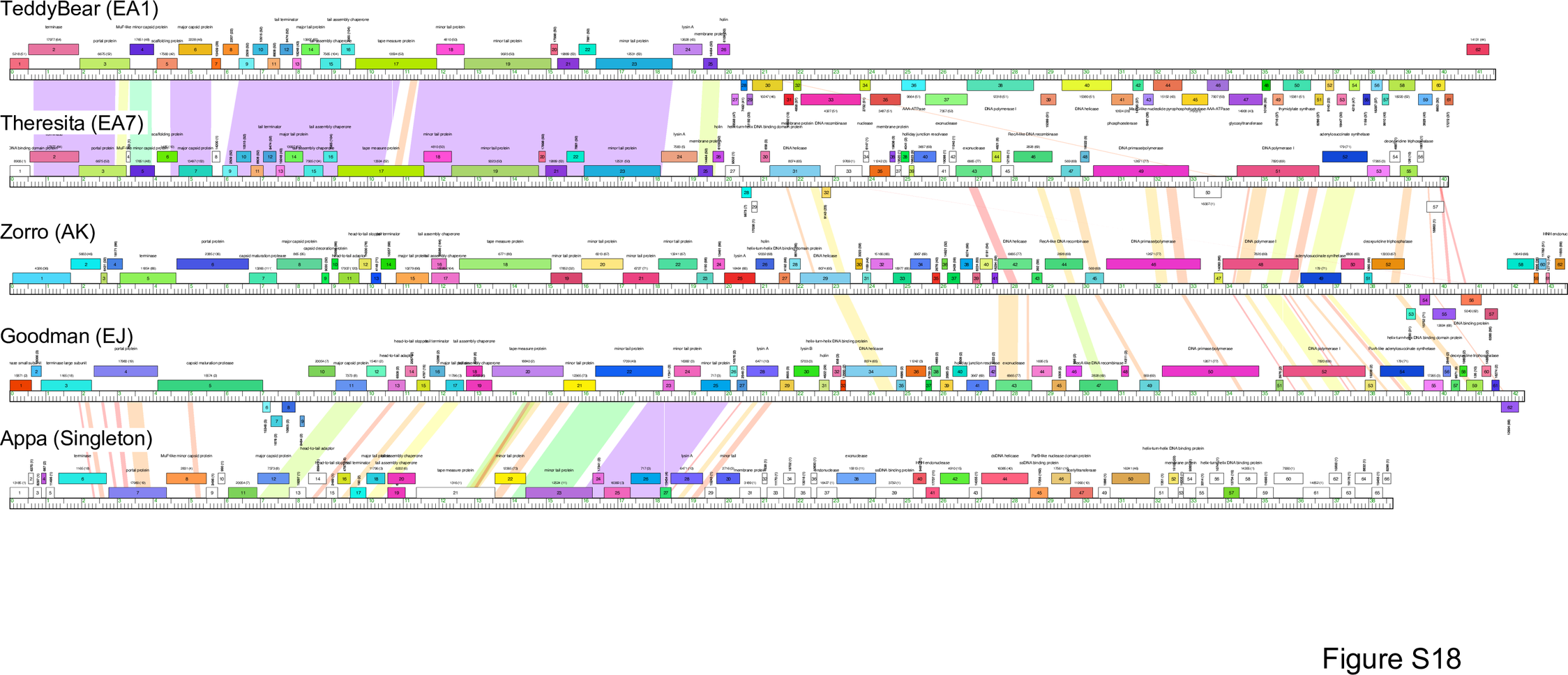

Supplement: S18 Fig — Alignments of genome maps of phages TeddyBear (Subcuster EA1), Theresita (Subcluster EA7), Zorro (Cluster AK), Goodman (Cluster EJ), and Appa (singleton). See S8 Fig for details. (TIF) [file pone.0234636.s020.tif]
